# The evolution of eyespots in skates and rays

**DOI:** 10.1038/s41559-026-03059-5

**Published:** 2026-04-24

**Authors:** Madicken Åkerman, Ana Cristina R. Gomes, David Wheatcroft, Niclas Kolm, Karl Gotthard, John L. Fitzpatrick

**Affiliations:** Department of Zoology, Stockholm University, Stockholm, Sweden

**Keywords:** Evolution, Behavioural ecology

## Abstract

Animals deploy a wide range of anti-predator adaptations, often combining multiple strategies into a ‘defence portfolio’. However, why certain defences evolve together whereas others are mutually exclusive remains unclear. Here we use a phylogenetic approach to examine the evolution of conspicuous visual markings—including eyespots and other conspicuous markings—alongside alternative anti-predator defences in skates and rays (Batoidea), a diverse group of cartilaginous fishes. We compiled data on the presence, type and number of conspicuous markings in 580 species of skates and rays, together with information on robust mechanical and electrical defences (venomous caudal stings and electric organs), adult body size and habitat depth as a proxy for light environment. We show that eyespots evolve stepwise from other conspicuous markings and that evolutionary gains of multiple markings are typically followed by reductions to fewer (often paired) markings. We further find that defence strategies follow alternative evolutionary trajectories: species possessing robust defences rarely evolve eyespots, whereas eyespots are favoured in smaller-bodied species lacking such defences and inhabiting well-lit waters where visual signals are most effective. These results show that accounting for multiple anti-predator defences can resolve why iconic anti-predator defences, such as eyespots, appear in some taxonomic groups but are conspicuously absent in others.

## Abstract (web summary)

A phylogenetic analysis of 580 skate and ray species shows that eyespots evolved stepwise and are favoured in small, shallow-water species lacking robust defences, revealing how trade-offs among anti-predator strategies shape the evolution of visual signals.

## Main

Prey have evolved a vast defence portfolio to avoid predation, including mechanical, visual, chemical and behavioural traits to foil predators^1–6^. With multiple defences available, defence portfolios can be influenced by trade-offs, including functional trade-offs in which investment in one type of defence reduces either the efficiency or need for investing in another^6^. Alternatively, selection can act on combinations of beneficial defences to form a syndrome of co-occurring traits (for example, refs. ^6–10^). How prey partition their defence portfolio is ultimately influenced by the fitness benefits of alternative defence strategies in preventing predation, with these benefits being shaped by ecological conditions experienced by prey^11^. Yet, despite an emerging framework^6^, few studies have examined the evolution of multiple anti-predator defences while simultaneously incorporating ecologically relevant factors at a macroevolutionary scale^10–13^.

**Fig. 1:**
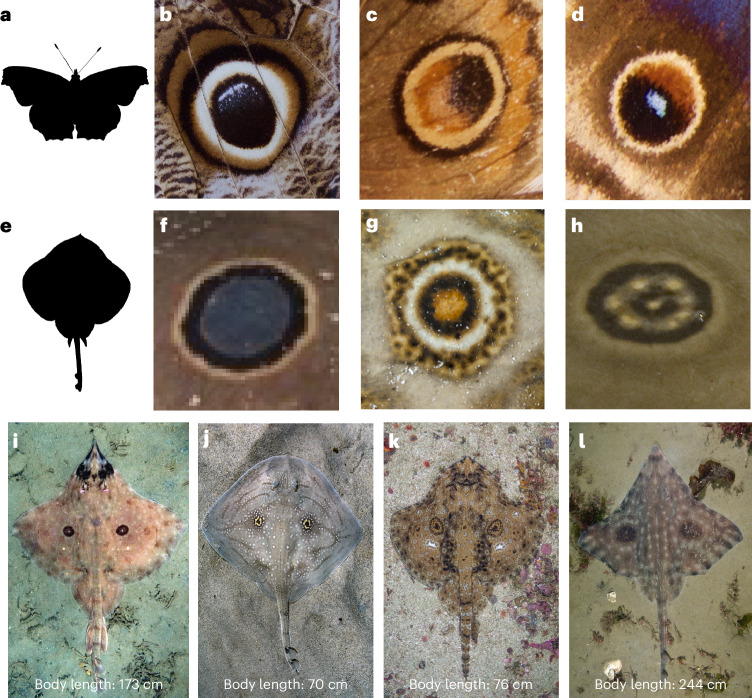
Conspicuous markings in skates and rays. **a**–**h**, Conspicuous markings commonly found in Lepidoptera (**a**), for example, in *Caligo brasiliensis* (**b**), *Junonia coenia* (**c**) and *Salamis temora* (**d**), superficially resemble the conspicuous markings found in skates and rays (**e**), for example, in *R. miraletus* (**f**), *D. ommata* (**g**) and *Rostroraja velezi* (**h**). **i**–**l**, In skates and rays, conspicuous markings are typically presented in pairs located centrally on the pectoral fins, as shown in *Caliraja rhina* (**i**), *Raja radula* (**j**), *Caliraja stellulata* (**k**) and *B. binoculata* (**l**). The average adult body sizes for species shown in **i**–**l** are presented. While conspicuous markings have not previously been studied in skates and rays, in other taxa, these markings are hypothesized to offer anti-predator defences to prey^24^. Silhouettes from PhyloPic under a Creative Commons license: *Inachis*, Gareth Monger (CC BY 3.0); *Psammobatis rudis*, Ignacio Contreras (CC BY 4.0). Photo credit: J.L.F. (**b**–**d**), Amy Rowley (**f**), Andy Murch (**g**–**l**).

Evolutionary coupling of mechanical and chemical defences with conspicuous visual signals (for example, aposematism) is a clear example of a defence portfolio syndrome^6,7^. However, it remains challenging to explain how visual signals co-evolve with other anti-predator defences in a defence portfolio when they do not advertise any danger to predators. A particularly striking example of such visual anti-predator defences are conspicuous markings, including the well-studied and taxonomically widespread circular ‘eyespots’ or ‘spots’^1,2,4^ (Fig. 1). Such conspicuous markings diverge from the animal’s remaining body pattern and are hypothesized to improve prey survival by drawing the attention of predators^14^, making these markings an initially counterintuitive anti-predator adaptation. However, ample experimental evidence shows that conspicuous markings can prevent or delay predator attacks either when presented suddenly (for example, refs. ^2,15,16^) or when displayed constantly (for example, refs. ^17–19^).

Several hypotheses address the anti-predator function(s) of conspicuous markings. Conspicuous markings are hypothesized to intimidate predators by causing an aversive response before an attack, thereby giving the prey an opportunity to flee (the ‘intimidation hypothesis’^2,5,16^). The intimidating function of conspicuous markings could be due to their resemblance to the vertebrate eye (potentially resembling the predator’s enemies, that is, the ‘eye-mimicry hypothesis’^2^) or because conspicuous and contrasting features of these markings startle predators and/or promote avoidance behaviour in predators (the ‘conspicuousness hypothesis’^17^). Conspicuous markings could alternatively deceive predators into perceiving that they have been detected by prey, leading predators to abort an attack that has a reduced likelihood of success (the ‘detection hypothesis’^20^). Finally, small conspicuous markings located on marginal parts of the body are hypothesized to draw attacks to non-vital parts of the prey, preventing lethal strikes by the predator and allowing prey to escape (the ‘deflection hypothesis’^1^). These potential anti-predator functions of conspicuous markings may work independently or synergistically depending on the predator and the point in the predation sequence when they are encountered.

Whether conspicuous markings effectively deter predators depends on a range of factors. First, the efficacy of conspicuous markings is influenced by the visual environment where they are perceived by predators, particularly as visual signals need to be perceived to function^21–23^. Second, the size and number of conspicuous markings can determine their effectiveness. For example, eyespots are more effective at deterring predators during bird–butterfly interactions when they are larger and when fewer markings are displayed^24^, a finding that probably explains the evolutionary trend of reductions in the number of eyespots displayed in some butterfly taxa^25^. Third, the anti-predator effectiveness of conspicuous markings interacts with body size in complex ways. If larger body sizes are associated with reduced predation risk^26^, as is the case when larger individuals are more difficult for predators to capture and/or handle^27^, then conspicuous markings may be more common in smaller species that are more prone to predation. By contrast, if larger prey are more easily detected by predators^23^, more profitable^28^ and/or better able to intimidate or mimic potential threats to predators^23,29^, then conspicuous markings are predicted to evolve in larger-bodied species. Macroevolutionary studies have thus far primarily focused on trying to understand the complex links between conspicuous markings, prey ecology and behavioural strategies for avoiding predation^23,30–33^. Yet, how these complex links evolve in the broader context of other anti-predator defences remains unclear.

Here we examine the co-evolution of conspicuous markings with alternative anti-predator defences and assess their evolutionary drivers in batoids (that is, skates and rays, superorder Batoidea), a diverse group of cartilaginous fishes comprising over 600 species. Anti-predator adaptations are expected to be under strong selection as skates and rays are preyed upon by numerous predators, including other skates and rays, sharks, marine mammals, birds of prey and waterbirds, and teleost fishes^34^. Indeed, skates and rays exhibit a range of divergent anti-predator strategies, making them an ideal model for examining defence portfolios. For example, some species of rays have evolved robust anti-predator defences, such as caudal stings and electric discharges, while other rays and skates have standard anti-predator defences such as mechanical defences (that is, thorns and denticles). Behavioural anti-predator strategies such as concealment (for example, burying in sediment or hiding among reef structures), flight responses or selecting habitats with fewer predators are widespread^35^. Skates and rays also exhibit a variety of conspicuous markings that superficially resemble those of terrestrial animals (Fig. 1a–f). While their evolution has never been formally studied, the presence of conspicuous markings in skates and rays is an open secret. Common names such as ‘eyespot skate’ (*Atlantoraja cyclophora*), ‘twineye skate’ (*Raja miraletus*; Fig. 1d) and ‘ocellated electric ray’ (*Diplobatis ommata*; Fig. 1e) show that conspicuous marking have long shaped the way skates and rays are described. Conspicuous markings are indeed conspicuous, being clearly visible on the dorsal surface in many skates and rays (Fig. 1g–j). In addition, within this group is probably the largest species ever described with conspicuous markings: the big skate (*Beringraja binoculata*), a species with conspicuous markings that reaches an adult total length >2 m (Fig. 1j). Skates and rays also exhibit extensive variation in key variables such as depth profiles (that is, light environments) and body size, making them an excellent model group to examine the evolution of multiple anti-predator defences in an ecologically and phylogenetically relevant framework.

In this study, we first examine hypotheses about the location, number and evolutionary dynamics of conspicuous marking in skates and rays (Table 1a,b). We then test the hypotheses that the evolution of conspicuous markings is shaped by (1) other anti-predator defences, (2) the visual environment and (3) body size (Table 1c,d). Initially, our analyses examine all types of conspicuous markings found in skates and rays, as conspicuous markings such as dots, stripes, rectangles and diamonds can be as effective as eyespots in deterring some types of predators (for example, avian predators^24^). However, because skates and rays exhibit both eyespots and other forms of conspicuous markings (Fig. 1), and hypotheses regarding the evolution of conspicuous markings were developed with eyespots in mind, we also examine the evolutionary drivers of both eyespots and other markings. In so doing, we provide the first macroevolutionary assessments of anti-predator adaptations across one of the oldest extant groups of jawed vertebrates.

**Table 1:** Predictions regarding the evolution of conspicuous markings in skates and rays

Trait	Prediction
**(a) Location and number of conspicuous markings**	
Conspicuous marking location	If conspicuous markings act to deflect predator attacks, then we predict that they would be located on marginal parts of the body^1^. If conspicuous markings intimidate predators or signal to predators that they have been detected, then we predict that they would be located on other (that is, non-marginal) locations on the body^16^,^17^.
Conspicuous marking number	Pairs of conspicuous markings could be more salient anti-predator cues, particularly if their function is to resemble vertebrate eyes (for example, ref. ^44^). A recent meta-analysis found weak (*R*^2^ = 2.46%), but significant, evidence that when a greater number of conspicuous markings are presented, their effectiveness declines^24^. If conspicuous markings have a role in predator deterrence in skates and rays, then we predict that fewer conspicuous markings, and possibly paired conspicuous markings, will be more common.
**(b) Evolutionary transitions in the type and number of conspicuous markings**	
Type of conspicuous marking	If eyespots are as effective as other conspicuous markings (as suggested by ref. ^24^), then transitions from no markings to either eyespots or other conspicuous markings should be equally likely to occur. If eyespots are more effective anti-predator markings than other markings, then we predict that gains in eyespots would be more common than gains in other conspicuous markings. Alternatively, if other markings provide even limited anti-predator benefits and/or are a necessary precursor on the evolutionary path to eyespots, then gains in other markings would precede the evolution of eyespots.
Number of conspicuous markings	Increases in the number of conspicuous markings reduce their anti-predator effectiveness^24^. Therefore, we predict that there would be evolutionary transitions to reduce conspicuous marking number.
**(c) Defence portfolios**	
Evolutionary trade-offs and syndromes	If conspicuous markings and robust anti-predator defences represent alternative anti-predator strategies, then due to either functional or energetics trade-offs^6^, we predict that conspicuous markings would not be as common in robustly defended species. Alternatively, if conspicuous markings advertise robust anti-predator defences in prey (akin to aposematic colouration), then we predict a positive correlation between conspicuous markings and prey defences. As electric organs appear more effective at deterring predation than caudal stings^34,37^, evolutionary associations may be more apparent in electric rays than stingrays and their allies. If eyespots provide greater anti-predator benefits than other markings, then we expect stronger co-evolutionary patterns for eyespots than other markings.
**(d) Evolutionary drivers of conspicuous markings**	
Anti-predator defence mechanisms	As described in (c), conspicuous markings are predicted to be more common in species lacking robust defences under the evolutionary trade-off hypothesis and more common in species with robust defences under the predator advertisement hypothesis. If eyespots provide greater anti-predator benefits than other markings, then we expect that anti-predator defence-mechanism-dependent patterns will be stronger for eyespots than for other markings.
Visual environments	Visual signals need to be perceptible to the intended recipient to function. Therefore, the efficacy of conspicuous markings is typically reduced as light intensity decreases^22,59,60^. In marine environments, the amount of light from the surface decreases with depth, creating depth-dependent visual environments. We predicted that reduced light levels at deeper depths will reduce the effectiveness of visual anti-predator signals such as conspicuous markings. As a result, species inhabiting deeper depths are predicted to have fewer conspicuous markings than species inhabiting shallower depths. If eyespots provide greater anti-predator benefits than other markings, then we expect that depth-dependent patterns will be stronger for eyespots than other markings.
Body size	Mortality rates typically decline with increasing body size in fishes (for example, ref. ^26^), as piscivores are more efficient at consuming smaller prey owing to gape limitation (that is, the physical size of the predator’s mouth) and larger fish can perform better predator-evasion behaviours^61^. Therefore, we predicted that conspicuous markings would be more likely to evolve in smaller skates and rays^62^. If eyespots provide greater anti-predator benefits than other markings, then we expect that body-size-dependent patterns will be stronger for eyespots than other markings.

Characteristics of conspicuous markings can be shaped by a wide range of selective forces. Here we summarize and motivate alternative predictions for the (a) location and number of conspicuous markings, (b) evolutionary transitions in the type and number of conspicuous markings, (c) defence portfolios and (d) evolutionary drivers of conspicuous markings.

## Results

### Conspicuous markings in skates and rays

**Fig. 2:**
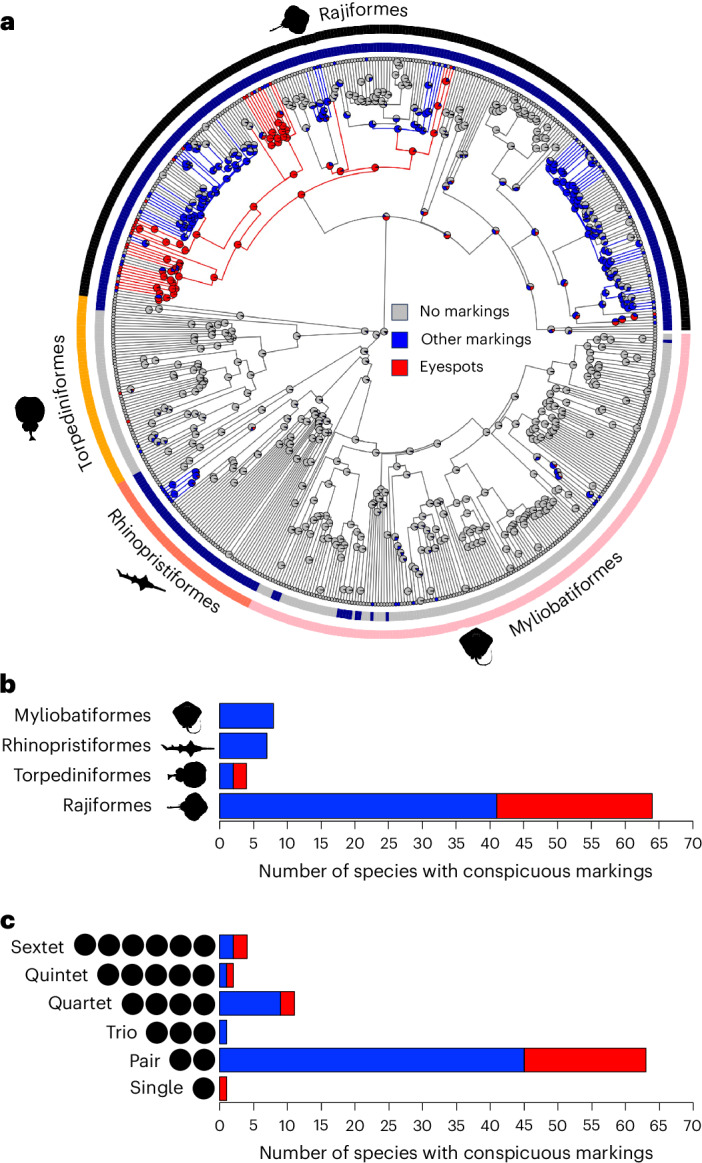
The evolution of conspicuous markings in skates and rays. **a**, Ancestral state reconstruction of conspicuous markings in skates and rays, depicting eyespots (red), other markings (blue) and the absence of conspicuous markings (grey) on the branches of the phylogeny. Pie charts show the character state distributions of nodal values. On the exterior of the phylogeny, the inner ring depicts species that lack robust anti-predator defences (blue) and species with robust anti-predator defences (grey). Note that conspicuous markings (interior plot, red and blue) are commonly associated with the absence of robust anti-predator defences (inner ring, blue). The outer ring labels the four orders of skates and rays, with black depicting the skates (order Rajiformes) and orange, peach and pink depicting the rays (orders Torpediniformes, Rhinopristiformes and Myliobatiformes, respectively). **b**, The number of species with conspicuous markings is presented for each of the four orders of skates and rays. **c**, The number of species displaying between one (single) and six (sextet) conspicuous markings on their ventral surface is also provided. Note that conspicuous markings are primarily found in skates (Rajiformes) and presented as pairs. In **b** and **c**, blue indicates other markings and red indicates eyespots. Silhouettes from PhyloPic under a Creative Commons license: *P.* *rudis*, Ignacio Contreras (CC BY 4.0); *Torpedo marmorata*, Steven Traver (CC0 1.0); *Pristis pristis*, C. Camilo Julián-Caballero (CC BY 3.0); *Neotrygon*, M. Kolmann (CC0 1.0).

We scored the presence and absence of conspicuous markings, defined as body markings that diverge from the remaining body pattern, from the dorsal side of 580 batoids (that is, skates and rays; Fig. 2a; ref. ^36^). This sampling effort represents >90% of extant species from the four batoid orders, including skates (order Rajiformes) and rays (electric rays (order Torpediniformes), shovelnose rays and their allies (order Rhinopristiformes), and stingrays and their allies (order Myliobatiformes)). Conspicuous markings were present in 14% of skates and rays (*n* = 83 of 580 species) and could broadly be subdivided into two categories: ‘eyespots’ and ‘other markings’ (Fig. 2a). Among the 83 species with conspicuous markings, 30% (*n* = 25) had circular markings with concentric marginal ring(s) of contrasting colours, which we refer to hereafter as ‘eyespots’. Among the remaining species (*n* = 58), the conspicuous markings took the form of spots (that is, markings lacking concentric marginal rings) that either were circular or had irregular shapes, which hereafter we collectively refer to as ‘other markings’.

Conspicuous markings were unevenly distributed across the skate and ray phylogeny (Fig. 2a,b). More than three times as many skates (Rajiformes; *n* = 64) had conspicuous markings compared with rays (Torpediniformes, Rhinopristiformes and Myliobatiformes; *n* = 19), despite similar numbers of species in each group (*n* = 279 skates, *n* = 301 rays). Among species with conspicuous markings, 92% (*n* = 23 of 25 species) of species with eyespots were skates (Fig. 2b). Eyespots were found only in skates (Rajiformes) and electric rays (Torpediniformes) and were more common in skates (*n* = 23 species) than in electric rays (*n* = 2 species; Fig. 2b). Nearly three quarters (71%, *n* = 41 of 58 species) of species with other markings were skates. However, other markings were found in all orders of skates and rays, albeit at varying frequencies (Fig. 2b). At the order level, 22.9% of skates (Rajiformes, *n* = 64 of 279 species) had conspicuous markings, while conspicuous markings were found in 12.7% of shovelnose rays and their allies (Rhinopristiformes, *n* = 7 of 55 species), 6.8% of electric rays (Torpediniformes, *n* = 4 of 59 species) and 4.3% of stingrays and their allies (Myliobatiformes, *n* = 8 of 187 species) (Fig. 2b).

Conspicuous markings (both eyespots and other markings) were overwhelmingly located centrally on the pectoral fin (*n* = 74 of 83 species; *n* = 22 with eyespots; *n* = 52 with other markings; Fig. 1i–l). By contrast, conspicuous markings were rarely (in <5% of species) located on the margin of the pectoral fins (*n* = 4 species; *n* = 1 with eyespots; *n* = 3 with other markings). An additional five species had conspicuous markings located both centrally and marginally (*n* = 2 with eyespots; *n* = 3 with other markings).

When present, the number of conspicuous markings ranged from one to six (Fig. 2c). Conspicuous markings were presented as a pair arranged symmetrically around the anterior–posterior midline in 76% of cases (*n* = 63 of 83 species; eyespots: *n* = 18; other markings: *n* = 45; Fig. 2c). In 19% of cases (*n* = 16 of 83 species), conspicuous markings were presented as multiples of two that were arranged symmetrically around the anterior–posterior midline, either as quartets (*n* = 12 species; eyespots: *n* = 3; other markings: *n* = 9) or as sextets (*n* = 4 species; eyespots: *n* = 2; other markings: *n* = 2; Fig. 2c). Thus, most conspicuous markings (~95%; *n* = 79 of 83 species) were displayed in groups of two or multiples of two (Fig. 2c). Uneven numbers of conspicuous markings were rare: only four species (two electric rays and two stingrays) had uneven numbers of conspicuous markings that were presented as a single marking (*n* = 1), a trio (*n* = 1) or quintets (*n* = 2; Fig. 2c).

### Ancestral state reconstructions

We used maximum likelihood ancestral state estimates to reconstruct the most probable root state for discrete traits across the batoid (that is, skates and rays) phylogeny. Ancestral state reconstructions revealed that the oldest common ancestor of skates and rays probably lacked conspicuous markings (Fig. 2a and Supplementary Table 1). Evolutionary losses (transitions (*q*) from the presence (0) to absence (1), *q*_10_ = 0.018) of conspicuous markings were ~9 times more likely than evolutionary gains (transitions (*q*) from the absence (0) to presence (1), *q*_01_ = 0.002) of conspicuous markings. Ancestral skates and rays most probably also lacked robust defences and inhabited shallower (that is, <200 m depth) water (Supplementary Table 1; note that between 0 m and 200 m is considered the ‘sunlight’ zone (that is, euphotic zone) where surface light penetrates water). Root ancestral state estimates were consistent for conspicuous markings and defence mechanism when we assessed skates and rays independently (Supplementary Table 1). However, in skate- or ray-specific analyses, the oldest common ancestor of skates probably inhabited deeper water (that is, >200 m) while the oldest common ancestor of rays probably inhabited shallower water (that is, <200 m; Supplementary Table 1).

### Evolutionary transitions in the type of conspicuous markings

**Fig. 3:**
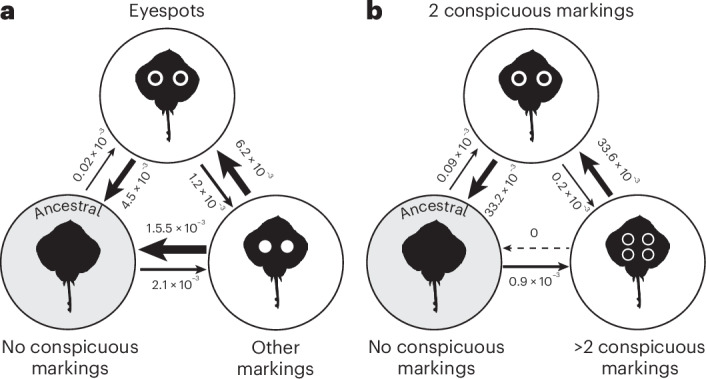
Examining evolutionary transitions in conspicuous marking type and number in skates and rays. **a**,**b**, Evolutionary transition parameters between different types of conspicuous markings (no conspicuous marking versus other markings versus eyespots) (**a**) and numbers of conspicuous markings (no conspicuous markings, 2 conspicuous markings and >2 conspicuous markings) (**b**), following an ARD model of transitions among character states. The arrows represent evolutionary transitions between character states, with the thicker arrows reflecting more likely evolutionary transition parameters. Evolutionary transition parameters are presented beside each transition arrow. The dashed arrow indicates a transition parameter value of zero. The ancestral character state is labelled and presented with a grey background. The image used for the >2 conspicuous markings character state is illustrative only; species with >2 conspicuous markings could have between 3 and 6 conspicuous markings. *P. rudis* silhouette by Ignacio Contreras from PhyloPic under a Creative Commons license (CC BY 4.0).

We reconstructed the most probable evolutionary path to and from different types of conspicuous markings (that is, no conspicuous markings, other markings or eyespots) in skates and rays using an ‘all rates different’ (ARD) model (Extended Data Table 1). From the ancestral state of having no conspicuous markings, evolutionary gains in other markings were ~100 times more likely to occur (that is, no markings → other markings) than evolutionary gains in eyespots (that is, no markings → eyespots; Fig. 3a). In addition, gaining eyespots from other markings (that is, other markings → eyespots) was ~300 times more likely than gaining eyespots from no conspicuous markings (that is, no markings → eyespots; Fig. 3a). Thus, gains in eyespots are most likely only after the evolution of other markings. Eyespots were then 3.75 times more likely to be lost (that is, eyespots → no markings) than to transition to other markings (that is, eyespots → other markings; Fig. 3a). Transition parameters indicating evolutionary losses of both eyespots and other markings were strong (that is, eyespots or other markings → no markings), suggesting that both forms of conspicuous markings are easier to lose than to gain (Fig. 3a).

**Extended Data Fig. 1:**
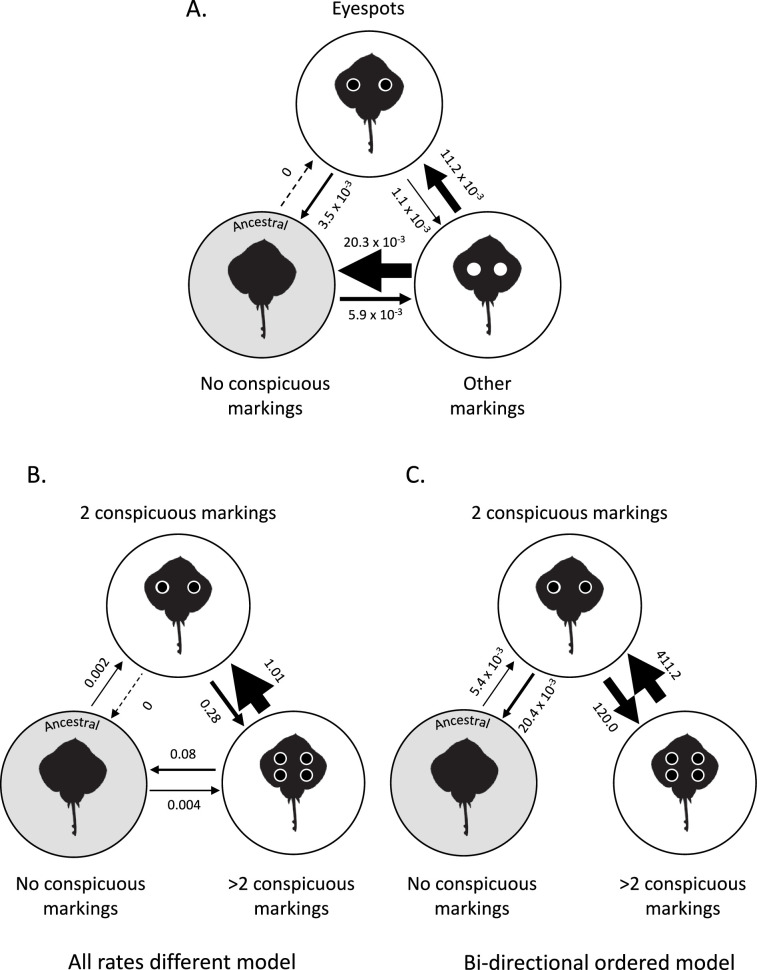
Examining evolutionary transitions in conspicuous marking type and number in skates. Evolutionary transitions parameters between different **A**. types of conspicuous markings (no conspicuous marking vs. other markings vs. eyespots) following an all-rates-different (ARD) model of transitions among character states and **B**-**C**. numbers of conspicuous markings (no conspicuous markings, 2 conspicuous markings and > 2 conspicuous markings), following **B**. an all-rates-different (ARD) model of transitions among character states and **C**. a bi-directional ordered model of transitions among character states. The arrows represent evolutionary transitions between character states, with the thicker arrows reflecting more likely evolutionary transition parameters. Evolutionary transition parameters are presented beside each transition arrow. Dashed arrows had transition parameter values of zero. The ancestral character state is labelled and presented with a grey background. The image used for the > 2 conspicuous markings character state is illustrative only; species with > 2 conspicuous markings could have between 3-6 conspicuous markings. *P. rudis* silhouettes by Ignacio Contreras from PhyloPic under a Creative Commons license (CC BY 4.0).

We next examined the most probable evolutionary path to and from different types of conspicuous markings using an ARD model (Extended Data Table 1) in skates (Rajiformes), in which conspicuous markings are most common (Fig. 2a,b). The evolutionary pattern of transitions among different types of conspicuous markings was qualitatively similar to the pattern observed among batoids (that is, skates and rays; Extended Data Fig. 1a).

### Evolutionary transitions in the number of conspicuous markings

We reconstructed the most probable evolutionary path to and from different numbers of conspicuous markings using an ARD model (Extended Data Table 1), including no (0), a pair (2) or several (>2) conspicuous markings in skates and rays (note that given the distribution of the number of conspicuous markings (Fig. 3b), we combined species with >2 into a single category and the one species with a single conspicuous marking was removed from this analysis). From the ancestral state of no conspicuous markings, evolutionary gains of >2 conspicuous markings (that is, 0 → >2) were 10 times more likely than evolutionary gains of a pair of conspicuous markings (that is, 0 → 2; Fig. 3b). Following evolutionary gains of >2 conspicuous markings, transition to a pair of conspicuous markings was strong (that is, >2 → 2), while back transitions to no conspicuous makings were virtually absent (Fig. 3b). Pairs of conspicuous markings were over 150 times more likely to transition to no conspicuous markings (that is, 2 → 0) than to >2 conspicuous markings (that is, 2 → >2; Fig. 3b). Thus, the most likely evolutionary route to gaining a pair of conspicuous markings was through first gaining >2 conspicuous markings.

When we assess the evolutionary dynamics of conspicuous marking numbers in skates, two evolutionary models were indistinguishable in our analyses of the most probable evolutionary path to and from different numbers of conspicuous markings (Extended Data Table 1). Compared with the analysis across batoids (that is, skates and rays), an analysis considering only skates (Rajiformes) using an ARD model revealed a qualitatively similar evolutionary pattern (Extended Data Fig. 1b). By contrast, a different evolutionary pattern among skates (Rajiformes) emerged when using a ‘bidirectional ordered’ model (Extended Data Fig. 1c), in which the only route to gaining conspicuous markings was from transitions from no conspicuous markings to pairs of conspicuous markings (that is, 0 → 2; Extended Data Fig. 1c).

### Evolutionary drivers of conspicuous markings

**Fig. 4:**
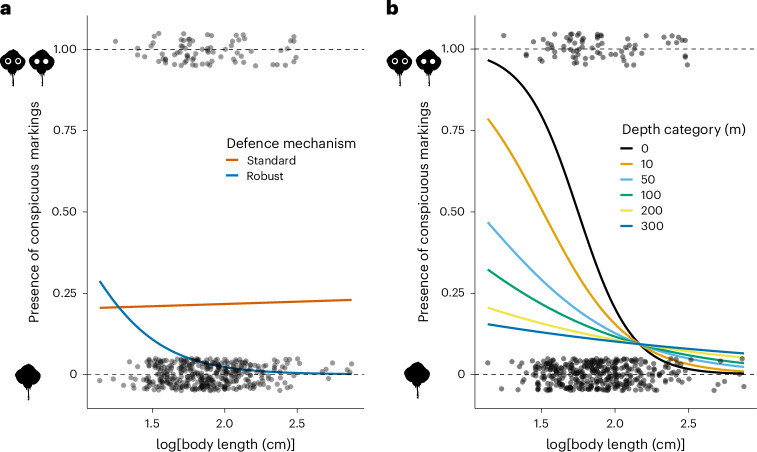
Predictors of the presence of conspicuous markings in skates and rays. Plots of the significant interaction terms from a phylogenetic multiple logistic regression across batoids (skates and rays). In both plots, points jittered around values of 0 and 1 on the *y*-axis indicate the absence (silhouette of a skate without conspicuous marking) and presence (silhouettes of skates with conspicuous markings, that is, eyespots or other markings) of conspicuous markings, respectively. **a**, The presence and absence of conspicuous markings in batoids depend on the interaction between adult body length and defence mechanism (standard versus robust, indicated by vermillion and blue lines, respectively). Across most of the body length distribution, species with standard defence mechanisms are more likely to have conspicuous markings. However, species with smaller adult body lengths are more likely to have conspicuous markings when they also have robust anti-predator defence. **b**, The presence and absence of conspicuous markings in batoids depend on the interaction between adult body length and depth. The different lines are fit at increasing depth categories (0–300 m) to illustrate the relationship between body length and depth. As depth categories increase, the relationship between body size and the presence of conspicuous markings becomes weaker. *P. rudis* silhouettes by Ignacio Contreras from PhyloPic under a Creative Commons license (CC BY 4.0).

We used phylogenetic logistic regression models to assess whether the presence of conspicuous markings was predicted by anti-predator defences (standard versus robust), adult body size, depth (that is, the midpoint in each species depth range profile) and the interactions between these factors across skates and rays. The presence of conspicuous markings was influenced by interactions between adult body length and the presence of robust defences and between adult body length and depth (Table 2a; note that all other interactions were non-significant). Conspicuous markings were more likely in species lacking robust defences across most adult body lengths (Fig. 4a)—this finding is consistent with the observation that conspicuous markings are most common in skates, an order that lacks robust defences (Fig. 2a,b). However, at the lowest end of the adult body size distribution, robustly defended species were more likely to have conspicuous markings than species lacking robust defences (Fig. 4a). Interrogation of the interaction between adult body size and depth revealed that species with smaller adult body sizes were more likely to have conspicuous markings, but only if they inhabited shallower depths (Fig. 4b).

**Table 2:** Phylogenetic logistic multiple regression models assessing whether the presence and absence of conspicuous markings are predicted by the defence mechanism (standard or robust), adult body length and depth in skates and rays

Predictor	Conspicuous markings				Estimate	*t*	*P*
	*n*	*n*_present_	*n*_absent_	*r*^2^_lik_			
**(a) Conspicuous markings**							
Defence mechanism	463	76	387	0.26	−4.31	1.51	0.13
Adult body length					−4.33	−1.97	**0.049**
Depth					−4.30	−2.42	**0.02**
Adult body length × defence mechanism					−3.40	−2.05	**0.04**
Adult body length × depth					1.98	2.19	**0.03**
**(b) Eyespots**							
Defence mechanism	411	24	387	0.30	−3.33	−2.57	**0.01**
Adult body length					−13.11	−2.82	**0.005**
Depth					−9.83	−2.83	**0.005**
Adult body length × depth					4.86	2.77	**0.006**
**(c) Other markings**							
Defence mechanism	439	52	387	0.13	6.19	3.19	**0.001**
Adult body length					−0.06	−0.16	0.88
Depth					−0.28	−1.22	0.22
Adult body length × defence mechanism					−4.44	−4.06	**0.00005**

Models compare species with (a) all conspicuous markings (that is, eyespots and other markings), (b) eyespots and (c) other markings against species lacking conspicuous markings. For each model, all possible interactions among predictors were included in the initial models. When interaction terms had *P* values > 0.10, they were removed from the model. Simplified models are presented in all cases, retaining only significant interaction terms. The sample size (*n*) reflects the number of species where data on all of the predictors were available. The number of species where conspicuous markings are present (*n*_present_) and absent (*n*_absent_), the correlation coefficient based on the likelihood of the fitted model (*r*^2^_lik_), the estimate, the *t* value and the *P* value are presented for each model. All models were two tailed and statistical inferences were derived from each model separately. Significant *P* values are presented in bold text.

**Extended Data Fig. 2:**
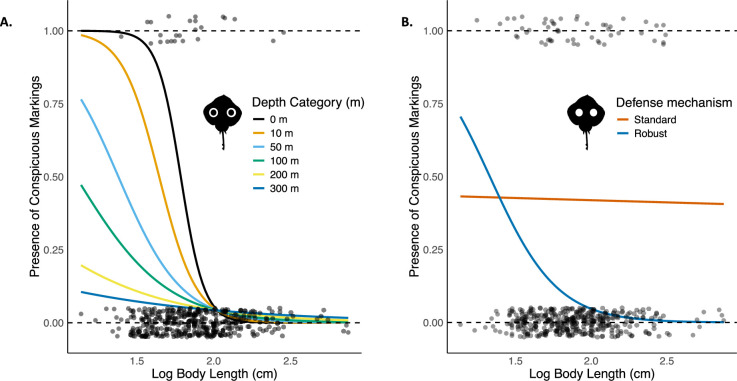
Predictors of the presence of eyespots and other markings in skates and rays. Plots of the significant interaction terms from a phylogenetic multiple logistic regression across Batoids (skates and rays) examining **A**. eyespots and **B**. other markings. In both plots, points jittered around values of 0 and 1 on the y-axis indicate the absence (values at 0) and presence (values at 1) of either **A**. eyespots or **B**. other markings, respectively. **A**. The presence/absence of eyespots in batoids depends on an interaction between adult body length and depth. The different lines are fit at increasing depth categories (from 0-300 m) to illustrate the relationship between body length and depth. As depth categories increase the relationship between body size and the presence of conspicuous markings becomes weaker. **B**. The presence/absence of other markings in batoids depends on an interaction between adult body length and defence mechanism (standard vs. robust, indicated by vermilion and blue lines, respectively). Across most of the body leng distribution, species with standard defence mechanisms are more likely to have conspicuous markings. However, species with smaller adult body lengths are more likely to have conspicuous markings when they also have robust anti-predator defence. *P. rudis* silhouettes by Ignacio Contreras from PhyloPic under a Creative Commons license (CC BY 4.0).

We next performed phylogenetic logistic regressions (as above) in which we modelled the evolution of eyespots or other markings separately. Eyespots were more common in species that lacked robust anti-predator defences (that is, primarily skates), and an interaction between adult body size and depth indicates that eyespots were more common in species with small adult body sizes provided they lived at shallower depths (Table 2b and Extended Data Fig. 2a). By contrast, other markings were more likely to be found in species with smaller adult body sizes, but only if they had robust anti-predator defences (Table 2c and Extended Data Fig. 2b).

We then performed a series of order-specific analyses and explored the impact of alternative depth measures on our findings. Broadly, these sensitivity analyses confirm that conspicuous markings are more common in smaller species that inhabit shallower waters (Supplementary Information) and are robust to alternative measures of depth (Supplementary Table 2).

**Extended Data Fig. 3:**
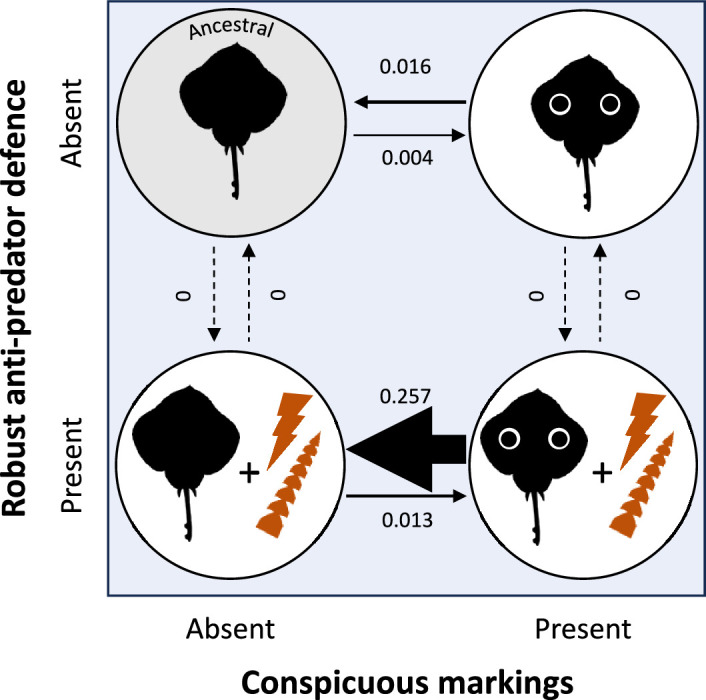
Evolutionary transition diagrams showing the most probable co-evolutionary pathways between robust anti-predator defences (presence/absence) and conspicuous markings (presence/absence). Robust anti-predator defences are illustrated in orange as either a lightning bolt, which represents the electric organ, or a serrated spine, which represents the caudal sting. The arrows represent evolutionary transitions between character states, with the thickness of the arrows reflecting the relative strength of the evolutionary transition parameter, which are indicated in the numbers beside each arrow. The ancestral character state is labelled and presented with a grey background. *P. rudis* silhouettes by Ignacio Contreras from PhyloPic under a Creative Commons license (CC BY 4.0).

**Extended Data Fig. 4:**
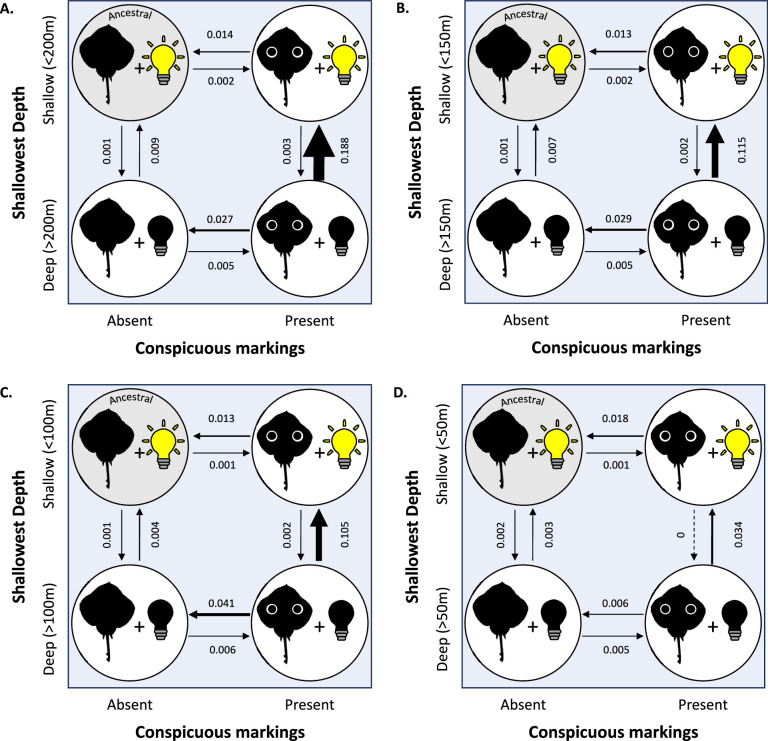
Evolutionary transition diagrams showing the most probable co-evolutionary pathways between conspicuous markings (presence/absence) and shallowest depth (shallow/deep). The depth to classify a species as either a shallow or deep species varies among the plots, with species considered as inhabiting a deep environment when their shallowest depth is **A**. <200 m, **B**. >150 m, **C**. >100 m, and **D**. >50 m. The yellow light bulb denotes a high light visual environment found at shallower depths and the black light bulb represents a low light visual environment found at deeper depths. The arrows represent evolutionary transitions between character states, with the thickness of the arrows reflecting the relative strength of the evolutionary transition parameter, which are indicated in the numbers beside each arrow. The results of these alternative cut-off values between shallow/deep species are qualitatively similar among themselves and with the data presented in the main text. The ancestral character state is labelled and presented with a grey background. *P. rudis* silhouettes by Ignacio Contreras from PhyloPic under a Creative Commons license (CC BY 4.0).

Finally, using discrete evolutionary models, we confirm that the presence of conspicuous markings is evolutionarily coupled with the absence of robust anti-predator defences and inhabiting shallower depths (Supplementary Table 3 and Extended Data Figs. 3 and 4). These findings were consistent in batoids (that is, skates and rays) and when focusing only on Rajiformes (that is, skates, in which conspicuous markings are most common; Supplementary Table 3). These additional analyses show that the combinations of conspicuous markings with robust anti-predator defences and conspicuous markings at deep depths are unstable evolutionarily states (Extended Data Figs. 3 and 4).

## Discussion

Our findings provide robust evidence that the evolution of conspicuous markings—and eyespots in particular—in skates and rays is shaped by alternative anti-predator defences and interactions between body size and the visual environment. Conspicuous markings are more commonly found in skate and ray species lacking robust anti-predator defences. Indeed, the majority of conspicuous markings were found in skates (Rajiformes), a group lacking robust anti-predator defences. Conspicuous markings, and eyespots in particular, were more likely to evolve in smaller species that lack robust defences, but only when they inhabit shallower depths (that is, brighter visual environments). By contrast, in robustly defended rays, conspicuous markings were more likely in species with small adult body sizes. Provided the risk of predation is stronger in smaller species and conspicuous markings have an anti-predator function in skates and rays, as in terrestrial systems, then our results suggest that conspicuous markings in species lacking robust defences probably evolve from their role in deterring predators^2,5,16,17,20^. By contrast, the caudal spines and electric discharges used by rays with robust anti-predator defences may be sufficient to deter and/or injure predators during close interactions and facilitate escape^35,37–39^, particularly among larger species. Together, these findings suggest that conspicuous markings are visual anti-predator signals in skates and rays that most probably provide protection to small species that may be more prone to predation, particularly in bright visual environments. Skates and rays therefore appear to deploy their defence portfolios in distinct evolutionary pathways that are mediated by size-dependent predation risk and the visual environment.

The prevalence of conspicuous markings in species lacking robust defences highlights the potential for functional trade-offs in anti-predator defences in skates and rays (sensu ref. ^6^). In the absence of robust anti-predator defences, conspicuous markings could offer a measure of anti-predator defence that these species otherwise lack^16,17,20^. Such an evolutionary trade-off appears to be robust in skates and rays, as we found that the combination of conspicuous markings and robust defences was an evolutionarily unstable state. Yet such an evolutionary trade-off can be counterintuitive. Drawing parallels from aposematic species, why would species that lack robust defences (that is, primarily skates) call attention to themselves with conspicuous markings? And why would robustly defended species (that is, most rays) not advertise their defences with conspicuous markings? We suggest that the negative co-evolutionary relationship in these defensive traits may be mediated by the relative costs and benefits of crypsis versus conspicuousness when skates and rays are avoiding predators^40^. Smaller species are often more prone to predation in marine environments^26,27^. If small skates rely on conspicuous markings to reduce their predation risk, then the anti-predator benefits of conspicuous markings would have to outweigh the costs of increasing conspicuousness. The costs and benefits of conspicuous markings may also interact with body-size-dependent changes in diet, as smaller skates predominantly feed on small crustaceans, while larger skates consume large prey (for example, bony and cartilaginous fishes^41^). Such size-dependent diet shifts may expose smaller skates to different predation pressures than larger species, particularly if smaller species spend more time foraging for smaller prey and less time hiding in the substrate. Conversely, robustly defended rays may rely on crypsis to initially avoid predator detection and attempt to deter predators only after they are detected with their caudal stings and electric organs (albeit with varying efficiency^34,37,39^). Validating these alternative explanations will require detailed field observations of both feeding and predation events in skates and rays, which can be challenging data to collect.

Eyespots in particular probably have a visual role in anti-predator defences in skate and ray species lacking robust defences. The low levels or absence of light at deeper depths probably blunts the fitness gains associated with investing in conspicuous markings. Indeed, eyespots were rarely found in skates and rays living at deeper depths. In line with this argument, tropical reef fish are thought to use eyespots against vision-based predators, which is enabled by the high visibility in reef waters, while some families of nocturnal fishes (for example, Holocentridae) completely lack eyespots^32^. Similarly, in our study, the only family of skates that lacked conspicuous markings (the smooth skates, Anacanthobatidae) is a deep-dwelling family typically found at depths >200 m where light does not penetrate. Reductions in light levels associated with nocturnality in mammals are also associated with reduced selection for visual-based anti-predator behaviours and instead selects for investment in noxious chemical defences^11^. Similarly, our findings suggest that the effectiveness of visual signals to deter predators is determined by the visual environment skates and rays inhabit, and as such, these signals are unlikely to evolve in darker visual environments.

We provide an examination of the evolutionary dynamics of conspicuous markings in skates and rays. Our analyses reveal that the earliest common ancestor of skates and rays most probably lacked eyespots, a pattern that is consistent with other marine fishes^32,33^. Gaining conspicuous markings in skates and rays is relatively rare but occurred multiple times independently, similar to the pattern observed in Lepidoptera^42,43^ and butterflyfishes^33^. The most probable evolutionary path for gaining conspicuous markings in skates and rays was by first gaining several (that is, >2) conspicuous markings that were most probably other markings (that is, not eyespots), followed by reductions in the number of markings and transitions to eyespots. This pattern is consistent with evidence in butterflies of an evolutionary trend of decreasing numbers of eyespots over evolutionary time^25^ and that displaying fewer eyespots offers greater anti-predator protection^24^. These evolutionary dynamics also suggest that other markings are a necessary precursor required for the evolution of eyespots, suggesting that in skates and rays, conspicuous markings are refined over time to resemble the classical eyespots found in terrestrial animals. In butterfly–bird interactions, eyespots are as effective in preventing predation as a wide range of other conspicuous markings^24^. Yet in skates and rays, the strong transitions from other markings to eyespots suggest that eyespots may be more effective in deterring predators than other markings. Crucially, our analyses also show that conspicuous markings are frequently lost, which matches eyespot evolution in terrestrial systems^19,42,43^. We suggest that the frequent gain and loss of conspicuous markings in skates and rays represent a balance between the relative benefits of avoiding detection (that is, crypsis) and deterring predators^40^. These anti-predator benefits are in turn probably shaped by body size and the visual environment, generating a dynamic selective landscape acting on conspicuous markings in skates and rays.

Testing the functional role of conspicuous markings during predation events would help to clarify mechanism(s) underlying their evolution. Because nearly all conspicuous markings in skates and rays are located centrally on the body, it is unlikely that conspicuous markings are used to deflect attacks to non-vital body parts^1^. Similarly, eyespots are frequently found on vital parts of the body (that is, close to the real eye) in coral reef fishes that inhabit the benthic parts of reefs^32^. One possibility is that skates and rays use conspicuous markings to intimidate or startle predators^5,16,17^. Skates and rays often bury themselves under the substrate to avoid detection^35^. For species lacking robust anti-predator defences, the sudden appearance of conspicuous markings as skates and rays emerge from the substrate could promote hesitation or avoidance behaviour at the final stages of the predation sequence. Alternatively, when not buried under the substrate, conspicuous markings could be used by skates and rays to signal to predators that they have been detected before an attack begins^4,20^. In this scenario, visual signals present on the dorsal side of skates and rays may be particularly effective as most skates and rays closely associate with the substrate, exposing their dorsal side to potential predators. Such (false) vigilance or perceptual advertisements to predators can be particularly effective against ambush predators, who are less likely to initiate attacks on prey if they perceive that they have been detected^20^.

The preponderance of pairs of conspicuous markings in skates and rays also suggests that pairs are more effective at deterring predators and/or that developmental constraints may limit conspicuous markings to being bilaterally symmetrical. In support of the former possibility, pairs of eyespots are more effective at deterring avian predators than control stimuli lacking any marking^44^. Experimentally comparing the efficacy of different numbers of conspicuous markings in deterring marine predators and trade-offs between conspicuous marking size and number are key next steps needed to shed light on their functional role as anti-predator visual signals. In addition, future research would benefit by also exploring the potential anti-predator function of other visually prominent features (for example, complex patterns and stripes) found in skates and rays^45^.

Despite a fascination that has lasted decades^1,2^, a key outstanding question is why conspicuous markings evolve in some taxa but are conspicuously absent in others. We provide macroevolutionary evidence that resolving this evolutionary puzzle requires an approach that considers the evolution of alternative anti-predator defences within a defence portfolio. Here we argue that functional trade-offs in the efficacy of alternative anti-predator defences are key to understanding the evolution of conspicuous markings in skates and rays. In other systems, resource trade-offs may be important. Therefore, assessing the co-evolution of conspicuous markings and alternative anti-predator defences (for example, chemical or mechanical defences) in both terrestrial and aquatic systems is an important next step to validate the general importance of trade-offs in shaping anti-predator defence portfolios.

## Methods

We scored the presence and absence of conspicuous marking of all skate and ray species in Last et al.^45^. Last et al.^45^ summarizes 633 skate and ray species, each species represented by a separate drawing, all illustrated by a single natural history artist. For each species, we assessed the dorsal side (the ventral side of skates and rays is typically white and not patterned). Species were categorized as having conspicuous markings when they had markings that diverge from the remaining body pattern. We then recorded whether conspicuous markings were located marginally (that is, on the marginal ~10–20% of the pectoral fin) or centrally (that is, on the midline, shoulder and remaining parts of the pectoral fin) and counted the number of conspicuous markings displayed. In cases in which markings covered large portions of the dorsal surface of the skate or ray (for example, as is often the case in freshwater Neotropical stingrays, family Potamotrygonidae), we classified these species as not having conspicuous markings, as their pattern of markings meant that they did not diverge from the remaining body pattern. To validate the accuracy of the drawing in Last et al.^45^, conspicuous markings were also scored from photographs whenever possible (Supplementary Tables 4 and 5). As there was a strong consistency between the classification of conspicuous markings from drawings and photographs (97% match; see Supplementary Information for details on method validation), we used the data obtained from Last et al.^45^ in all analyses as this allowed for a greater number of species to be considered in our analyses. Of the 633 species initially considered, we focused all subsequent analyses on the 580 species that were present in the Elasmobranch-wide phylogeny available from the Chondrichthyan Tree of Life (http://sharksrays.org/).

For each of the 580 skate and ray species in our dataset^36^, we compiled information on their type of anti-predator defence. Species were categorized as having robust anti-predator defences (sensu ref. ^13^) when they possessed caudal stings (in stingrays and allies) or electric organs (in electric rays) capable of producing electric discharges (*n* = 229 of 580 species) that can be used to defend against predators^35^ or as having standard anti-predator defences when these robust defences were absent (*n* = 351 of 580 species). We compiled data on maximum adult body length (*n* = 566). We also collected data on hatching and birth length, using midpoint values when a range of values was given. However, because adult body length and hatching and birth length were correlated (*r* = 0.65, *P* < 0.001, *n* = 265), and the available sample size for adult body length (*n* = 566) was more than double the sample size available for birth and hatching length (*n* = 265), we considered only adult body length in our analyses. For body length, adult total length and disc width (for ray species with elongated tails) were used interchangeably as a measure of body size as these measures are tightly correlated (*r* = 0.84, *P* < 0.01, from the *n* = 92 species in Last et al.^45^ in which data on body size were available for both measures). Importantly, in order-specific analyses, a standard body size measure is used in all cases except when considering the stingrays and their allies (order Myliobatiformes; Supplementary Table 2). However, additional sensitivity analyses revealed that removing species with only disc width data available did not qualitatively change our main findings (Supplementary Information). We also compiled data on the shallowest and deepest depths in which the species was recorded, and calculated the midpoint between the shallowest and deepest recorded depths (*n* = 472 (ref. ^36^)). Depth is usually reported as a range, with both shallowest and deepest recorded depths specified, with these two depth measures being correlated (*r* = 0.76, *P* < 0.01, *n* = 472). When assessing depth, we used the midpoint depth values in our main analyses. However, to examine how alternative depth values influence our results, we also examine shallowest depth values in sensitivity analyses (Supplementary Table 2). Conspicuous markings can have a role in sexual selection and can therefore be sexually dimorphic^46,47^. However, we were unable to test for a sexually selected function of conspicuous markings as there is no obvious sexual dimorphism in these markings in skates or rays^45^. Data were primarily sourced from Fishbase^48^. Additional data were provided by Last et al.^45^ or from the literature^36^.

### Batoid phylogeny

All analyses were performed in RStudio v 2024.04.1+748 (ref. ^49^). To control for non-independence data due to a shared ancestry, we used a recent phylogeny for batoids^50^ by extracting a distribution of 10,000 Elasmobranchii phylogenies (that is, 1,192 species of sharks, batoids and chimaeras) from the Chondrichthyan Tree of Life (http://sharksrays.org/). TreeAnnotator (v.1.10.4) in BEAST^51^ was used to summarize the information from this sample of trees into a single consensus tree (BurnIn: 1,000; posterior probability limit: 0.5; target tree type: maximum clade; node heights: posterior median). The tree was trimmed in R using the ‘drop.tip’ function in the ‘geiger’ package^52^ to include only the superorder Batoidea, including four orders: skates (Rajiformes), electric rays (order Torpediniformes), shovelnose rays and their allies (order Rhinopristiformes), and stingrays and their allies (order Myliobatiformes). The resulting phylogeny consisted of 23 families, 86 genera and 639 species. We then resolved species names between the phylogeny and names listed in Last et al.^45^ to account for updates in taxonomy. A total of 52 species present either in the phylogeny or in our dataset did not match and were therefore removed and a further 7 species were listed in Last et al.^45^ but not included in the phylogeny, reducing the total sample size to 580 species.

### Ancestral state reconstruction

We determined ancestral states for a series of discrete character traits in skates and rays. First, we used the ‘fitMK’ function in the ‘phytools’ package^53^ to identify which evolutionary model best explains transition rates between and among different character states. In all cases, we specified that the previous distribution of different character states at the root node was equal. For traits with binary discrete character states, we compared the model fits of equal rate (ER) and ARD models. Traits with binary discrete character states included conspicuous markings (absent: *n* = 497; present: *n* = 83), defence mechanism (standard: *n* = 351; robust: *n* = 229) and depth (based on a 200-m cut-off: shallow: *n* = 351; deep: *n* = 121). For traits with three discrete character states, we compared the model fits of ER, ARD and symmetrical (SYM) models. Traits with three discrete character states included conspicuous marking type (no conspicuous marking: *n* = 497; other conspicuous marking: *n* = 58; eyespot: *n* = 25) and number of conspicuous markings (no conspicuous markings (0): *n* = 497; a pair of conspicuous markings (2): *n* = 63; or more than two conspicuous markings (>2): *n* = 19; note that we grouped species with more than two conspicuous markings into a single category as this accounts for the relative paucity of species with between 3 and 6 conspicuous markings (Fig. 2c). The model that best explained the discrete trait’s evolution was determined by comparing log likelihood and Akaike information criterion (AIC) values to compare model fits. An ARD model of evolution best fits the observed transition rates in conspicuous markings, conspicuous marking types and the number of conspicuous markings, and in most cases for depth (Supplementary Table 1). By contrast, an ARD and ER model were indistinguishable for defence mechanisms and some depth analyses (Supplementary Table 1). Specifying the best-fitting model (or both models when necessary), we then used Markov models of character evolution using the corHmm package^54^. We then determined the marginal ancestral state reconstruction value at the root for each character by extracting the marginal posterior probabilities of each character state as the root node (Supplementary Table 1). Analyses were initially performed across skates and rays (batoids), followed by analyses considering only skates (Rajiformes), in which conspicuous markings are more common, and analyses considering only rays (Myliobatiformes, Torpediniformes and Rhinopristiformes).

### Modelling the evolution of the type and number of conspicuous markings

We fitted models of discrete character evolution to calculate rates of evolutionary transition among different types and numbers of conspicuous markings. To do this, we categorized species as having different types of conspicuous markings (no conspicuous marking: *n* = 497; other conspicuous marking: *n* = 58; eyespot: *n* = 25) and different numbers of conspicuous markings (no conspicuous markings (0): *n* = 497; a pair of conspicuous markings (2): *n* = 63; or more than two conspicuous markings (>2): *n* = 19; note that as above, we grouped species with more than two conspicuous markings into a single category). We then determined the transition matrix that best explained state transitions in our data by fitting Markov models for for K discrete character states (that is, MK models) with equal rates (ERs; all transition rates occur at equal rates), symmetric rates (SYM; symmetrical transition rates occur at equal rates), ARD (all transition rates occur at different estimated rates) and bidirectional ordered rates of state transitions (transition rates occur at different estimated rates and only among ordered categories: 0←→2←→>2), and comparing them by the obtained log likelihood and AIC values. The best-fitting model (ARD) indicated that all rates of gains and losses of conspicuous markings differed between character states (Extended Data Table 1). MK models were fitted in the ‘phytools’ package^53^ using the ‘fitMk’ function, implementing the FitzJohn et al.^55^ previous distribution at the root node. For some models, we found that optimization could not converge; thus, following the guidance provided by Revell and Harmon^56^, we replicated the same models 100 times and repeated models with the same transition matrixes using the ‘fitDiscrete’ function available in the ‘geiger’ package^52^ to check for consistency in the best-fitting model (Extended Data Table 1).

### Phylogenetic logistic regressions

To test how a range of predictors influence the evolution of conspicuous markings in skates and rays, we performed phylogenetic multiple logistic regression models using the ‘phyloglm’ function in the ‘phylolm’ package^57^. We first treated the presence and absence of conspicuous markings (that is, including eyespots and other markings) as a binary response variable and ran models assessing whether conspicuous markings were predicted by defence mechanism (standard versus robust), adult body length and midpoint depth and included all possible interactions. Continuous variables (body length and depth) were log_10_ transformed before analysis. After fitting this full model, we removed interaction terms where *P* > 0.10 and presented only the simplified models. We then repeated this modelling approach in two subsequent models that considered only the presence and absence of eyespots or the presence and absence of other markings (note that given the model structure, it was not possible to fit a single model examining three states of conspicuous markings). Finally, as the frequency of conspicuous markings differed among batoid orders (Fig. 2b), we used the same model structure to examine order-specific drivers of conspicuous marking evolution (the only exception being that defence mechanisms were not considered as there is little or no within-order variation in this trait; Supplementary Table 2).

### Correlated evolution of conspicuous markings in skates and rays

We tested whether the evolution of the presence and absence of conspicuous markings was correlated with either anti-predator defences (standard versus robust) or depth (shallow, <200 m versus deep, >200 m) in skates and rays. While light levels undoubtedly decrease with depth, we used a strict cut-off of 200 m for assigning species as shallow versus deep because little or no light reaches beyond this depth in the ocean. We then used the ‘fitPagel’ function in the ‘phytools’ package^53^ to test for correlated evolution between these discrete characters. This function implements Pagel’s method^58^ for comparing an independent model of character evolution, in which character states change independently from one another, with a dependent model of character evolution, in which the rate of change in one character is dependent on the other. For each model, we compared three alternative dependent models: (1) a model in which transitions in conspicuous markings depend on anti-predator defences and depth, but not the reverse; (2) a model in which transitions in anti-predator defences and depth depend on conspicuous markings, but not the reverse; and (3) a full model in which conspicuous markings depend on anti-predator defences and depth and vice versa. The best fitting of the alternative dependent models was determined by comparing AIC values (Supplementary Table 3). The independent and best-fitting dependent model was then compared using likelihood ratio tests. If the dependent model differed significantly from the independent model, then we assessed the strength of the evolutionary transition parameters to determine the most probable evolutionary transitions among character states.

### Reporting summary

Further information on research design is available in the Nature Portfolio Reporting Summary linked to this article.

## Supplementary information

Supplementary Information Supplementary Tables 1–5, methods and results.

Reporting Summary

## Figures and Tables

**Extended Data Table 1:**
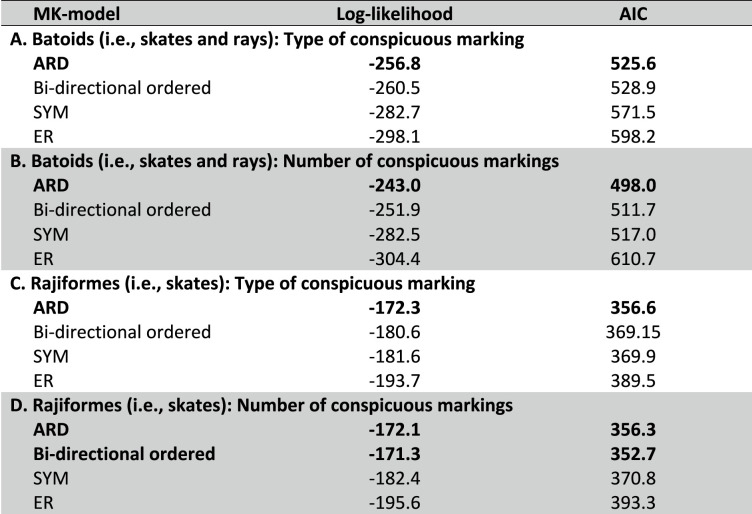
Comparing evolutionary models for the A. type and B. number of conspicuous markings in Batoids (that is, skates and rays) and the C. type and D. number of conspicuous markings in Rajiformes (that is, skates)

To identify the appropriate evolutionary model when modelling trait evolution we first compared the fit of alternative evolutionary models using MK-models. We compared the model fits from MK-models of equal rates (ER; all transition rates occur at equal rates), symmetric rates (SYM; symmetrical transition rates occur at equal rates), all rates different (ARD; all transition rates occur at different estimated rates), and bi-directional ordered rates of state transitions (transition rates occur at different estimated rates and only among ordered categories: 0←→2←→ > 2). For each model the log-likelihood and AIC values are presented. The best fitting models that were used in the ancestral state reconstructions are presented in bold. Models where AIC values were > 2 units greater than AIC values from the best fitting model were considered poorer fits. We confirming our model fits using geiger (see Methods), ARD was consistently the best fitting model (ARD: log-likelihood = −242.9, AIC = 497.9; bi-directional ordered: log-likelihood = −251.0, AIC = 510.0; SYM: log-likelihood = −282.5, AIC = 571.0; ER: log-likelihood = −304.4, AIC = 610.7), and phytools replication values were non-overlaping between the different models.

## Data Availability

All data (ref. ^36^) are available via the Open Science Framework at https://osf.io/k6cy9/overview?view_only=8d3769f32fbf4a1ba4ffb0f4c26e2c67.

## Code availability

The code used in the analyses (ref. ^36^) is available via the Open Science Framework at https://osf.io/k6cy9/overview?view_only=8d3769f32fbf4a1ba4ffb0f4c26e2c67.
