# Supplementary material for: The evolution of eyespots in skates and rays
Source: Nat Ecol Evol. 2026 Apr 24;10(6):1184–94. doi: 10.1038/s41559-026-03059-5 (PMC13253327; doi:10.1038/s41559-026-03059-5)
Supplement: Supplementary file 1 — Supplementary Tables 1–5, methods and results. [file 41559_2026_3059_MOESM1_ESM.pdf]

nature ecology & evolution

Supplementary information

https://doi.org/10.1038/s41559-026-03059-5

The evolution of eyespots in skates and rays

In the format provided by the authors and unedited

Supplementary Information

Validating the use of drawings to classify distinct markings with photographs

We scored the presence/absence of conspicuous markings from illustrations produced by a single natural history artist that were presented in Last et al. (*48*). When species are illustrated, the ambiguities from photos (such as lighting conditions) can be diminished. However, a single illustration does not reflect possible variation within the species, including intra-specific differences among individuals and life stage. Therefore, to validate the accuracy of the drawing in Last et al. (*48*), conspicuous markings were also scored from photographs for all available species using the same approach we applied to the drawings from Last et al. (*48*). To ensure that the photographs displayed the correct species, we only used scientific resources validated by experts such as museums, universities, and governmental agencies (Supplementary Table 4). Both scientific names, and possible synonym names from older classifications, were used as search terms. All non-duplicate photographs where the body pattern could be observed, and where at least half of the dorsal area (including one of the pectoral fins) was visible, were used. As body patterns can be difficult to observe after preservation, we excluded photographs of specimens that were obviously dehydrated. Care was taken to avoid using photographs where the same animal was presented in different arrangements. We focused our examination of photographs on the 580 skate and ray species that were included in our analyses. Intra-specific variation in conspicuous markings has been observed in some skates and rays (e.g. *Rhynchobatus australiae*, *67*). Therefore, we scored species as having conspicuous markings if they were present in any of the available photographs.

In total, 1602 photographs were collected, distributed over 408 species (mean number of photographs/species ± SE = 3.76 ± 0.09, with a range of 1-18 photographs per species), of which 63 species had been scored with conspicuous markings (Supplementary Table 4,5). When scoring for the presence/absence of conspicuous markings from these photographs, 97% (n = 394) of the species scored matched the scoring derived from the drawings in Last et al. (*48,* Supplementary Table 5). Conspicuous markings were present in photographs for

all of the 63 species scored as having conspicuous markings based on drawings in Last et al. (*48,* Supplementary Table 5). For species scored as not having conspicuous markings based on drawings in Last et al. (*48*), in 14 cases we found some photographs where these species would be classified as having conspicuous markings (Supplementary Table 5). All of the 14 species where there was disagreement between the drawings from Last et al. (*48*) and the photographs were from the order Rajiformes (skates). In our analyses, we used a conservative approach and continued to classify these species as lacking conspicuous markings.

Comparing alternative measures of body size

Body size for most species was reported as the total length, measured as the length from the snout to the end of the tail. However, in some ray families Stingrays (*Dasyatidae*), Butterfly rays (*Gymnuridae*), Neotropical stingrays (*Potamotrygonidae*), Eagle rays (*Myliobatidae*), Pelagic eagle rays (*Aetobatidae*), Cownose rays (*Rhinopteridae*), and Devilrays (*Mobulidae*), the tail extends long beyond the body, which can dramatically inflate the total length. In addition, the tail easily breaks in species from these families during capture, potentially causing inaccurate measurements of total length. Therefore, the disc width (the length between the ends of the pectoral fins) is more commonly used to quantify body size for these families. In Last et al. (*48*) where data on body size was available for both measures, total length and disc width were tightly correlated ($r = 0.84$, $p<0.01$, $n = 92$). Therefore, to increase the sample size, we used total length and disc width interchangeably as a measure of body size.

Nevertheless, we explored if the inclusion of disc width as a body size measure influenced the results of our phylogenetic logistic regression models. To do this, we removed families where disc width is used to measure body size and re-ran our phylogenetic logistic regression models without these species. These additional analyses revealed qualitatively similar patterns as detected in our main analysis (n_{total}=240, r^2_{lik}=0.28): namely, across Batoids the presence of conspicuous markings was influenced by defence mechanisms (conspicuous markings are more common in species lacking robust defences, estimate = -2.19, t = -3.08, p = 0.02), and while adult body length (estimate = -4.51, t = -1.91, p = 0.06) and depth (estimate = -4.37, t = -2.23, p = 0.03) showed either suggestive or clear evidence

of an association with conspicuous markings, we focus on the interaction between adult body length and a species mid-point depth value (estimate = 1.99, t = 1.99, p = 0.046) detected in this model, which indicates that smaller species are more likely to have conspicuous markings when they inhabit shallower depths.

Order-specific phylogenetic logistic regressions

In skates (Rajiformes), where eyespots are most common, conspicuous markings were more likely to evolve in species living in shallower waters (Supplementary Tables 2A). However, eyespots in skates were more likely to evolve in smaller species that inhabited shallower waters, while other markings in skates were more likely to evolve in species inhabiting shallower depth regardless of body size (Supplementary Tables 2B,C). Among rays, conspicuous markings were more likely in species with smaller adult body lengths in stingrays and their allies (Myliobatiformes, Supplementary Tables 2D). In electric rays (Torpediniformes), a statistical trend suggested that species with smaller adult body sizes were more likely to have conspicuous markings if they inhabited shallower waters (Supplementary Tables 2E). Finally, in shovelnose rays and their allies (Rhinopristiformes), an order that lacks robust defences, there was no association between either adult body length or depth and the presence of conspicuous markings (Supplementary Tables 2F). The results were broadly consistent when considering shallowest depth measures as an alternative metric of a species depth value (Supplementary Tables 2G-O).

Co-evolution between alternative anti-predator defences

We used discrete evolutionary models to test for evolutionary correlations between alternative anti-predator defences. Conspicuous markings were more likely to evolve in skates and rays with standard anti-predator defences and less likely to evolve in species with robust anti-predator defences (Extended Data Figure S3, Supplementary Table 3A). The best fitting discrete model revealed that the conspicuous markings were dependent on anti-predator defence mechanisms, but not vice-versa (Supplementary Table 3A). Examination of evolutionary transition parameters revealed that losses of conspicuous markings were 16 times more likely in species with robust anti-predator defences than in species with standard defences (Extended Data Figure S3). The lack of transitions between the presence

and absence of robust anti-predator defences is due to the basal splits between those Orders that have robust anti-predator defences (i.e., most Myliobatiformes and Torpediniformes) and those that do not have robust anti-predator defences (i.e., Rajiformes and Rhinopristiformes, see Figure 2A).

The visual environment and the evolution of conspicuous markings

To test the hypothesis that the visual environment influences the evolution of conspicuous markings, we compared species based on their depth profiles. We created discrete categories based on the physical properties associated with light transmission through water. Specifically, species were classified as either inhabiting *shallow* depths if they were found in the 'sunlight' zone between 0-200m (i.e., the euphotic zone), where surface light penetrates the water. Alternatively, species were classified as inhabiting *deep* depths if they were found in the 'twilight' or 'midnight' zones >200m where light rarely extends (i.e., the dysphotic or aphotic zones, Extended Data Figure S4, Supplementary Table 3). We also explored how alternative classifications of shallow and deep depths influenced our results in a series of sensitivity analyses focused on depth cut-offs of 150m, 100m and 50 m (Extended Data Figure 4, Supplementary Table 3A). The presence/absence of conspicuous markings was evolutionary coupled with species depth profiles (Supplementary Table 3). Examining the evolutionary transitions among character states revealed that conspicuous markings were evolutionarily unstable in species inhabiting deep depths: there were evolutionary transitions driving either the presence of conspicuous marking in shallow environments or alternatively the loss of conspicuous markings in deep environments in all of the alternative depth cut offs (Extended Data Figure S4). These findings were robust to alternative classifications of shallow vs. deep species (Extended Data Figure 4, Supplementary Table 3) and remained consistent when we focused our analyses on Rajiformes (i.e., skates, where conspicuous markings are most common, Supplementary Table 3B).

Supplementary Table 1. Ancestral state reconstructions at the root for discrete character traits in A. Batoids (i.e., skates and rays), B. Rajiformes, and C. Rhinopristiformes, Torpediniformes, and Myliobatiformes (i.e., rays). To identify the appropriate evolutionary model for the ancestral state reconstruction we first compared the fit of alternative evolutionary models using MK-models. For binary discrete character states, we compared the model fits of equal rate (ER) and all rates different (ARD) models. For traits with three discrete character states, we compared the model fits of ER, ARD and symmetrical (SYM) models. For each evolutionary model the log-likelihood [Log(L)], Akaike Information Criterion (AIC) and AIC weight (w_i) are presented. We selected the best fitting dependent model by comparing AIC values. The best fitting models that were used in the ancestral state reconstructions are presented in bold. Models where AIC values were >2 units greater than AIC values from the best fitting model were considered poorer fits. In cases where AIC values from multiple models were within 2 units we performed ancestral state reconstructions for each multiple model. The ancestral state probability at the root is presented for each trait state, with the sample size (N) and state probability provided. Ancestral states for defence mechanisms were examined for A. Batoids and C. Rhinopristiformes, Torpediniformes, and Myliobatiformes as there is variation in this trait in these taxonomic groups. In contrast, since there is no variation in defence mechanisms in **B.** Rajiformes this analysis was omitted for this Order.

Traits and discrete character states	Evolutionary Model				Ancestral State at the Root		
	Model	Log(L)	AIC	w_i	Trait State	N	State Probability
**A. Batoids (i.e., skates and rays)**							
Conspicuous markings	**ARD**	**-209.6**	**423.1**	**1**	Absent	497	0.91
(absent/present)	ER	-233.7	469.4.7	0	Present	83	0.09
Conspicuous marking type	**ARD**	**-243.2**	**498.4**	**1**	No marking	497	0.89
(no marking/other marking/eyespot)	SYM	-282.7	571.5	0	Other marking	58	0.06
	ER	-298.1	598.2	0	Eyespot	25	0.05
Number of conspicuous markings	**ARD**	**-242.9**	**497.9**	**1**	None	497	0.93
(none, pair, >2)	SYM	-282.5	573.0	0	Pair	63	0.03
	ER	-304.4	610.7	0	>2	19	0.04
Defence mechanism	**ER**	**-50.6**	**103.1**	**0.56**	Standard	351	ER: 0.9996 / ARD: 0.999
(standard/robust)	**ARD**	**-49.8**	**103.7**	**0.44**	Robust	229	ER: 0.0004 / ARD: 0.001
Mid-point Depth (200m cut-off)	**ARD**	**-268.7**	**541.3**	**1**	Shallow	351	0.85
(shallow/deep)	ER	-327.2	656.3	0	Deep	121	0.15
Mid-point Depth (150m cut-off)	**ARD**	**-286.1**	**576.2**	**1**	Shallow	339	0.85
(shallow/deep)	ER	-327.2	656.3	0	Deep	139	0.15
Mid-point Depth (100m cut-off)	**ARD**	**-307.3**	**618.6**	**1**	Shallow	304	0.87
(shallow/deep)	ER	-327.2	656.3	0	Deep	168	0.13
Mid-point Depth (50m cut-off)	**ARD**	**-225.7**	**455.3**	**1**	Shallow	251	0.88
(shallow/deep)	ER	-327.5	656.9	0	Deep	221	0.12
Shallowest Depth (200m cut-off)	**ARD**	**-268.7**	**541.3**	**1**	Shallow	351	0.85
(shallow/deep)	ER	-327.2	656.3	0	Deep	121	0.15
Shallowest Depth (150m cut-off)	**ARD**	**-286.1**	**576.2**	**1**	Shallow	339	0.85
(shallow/deep)	ER	-327.2	656.3	0	Deep	139	0.15
Shallowest Depth (100m cut-off)	**ARD**	**-307.3**	**618.6**	**1**	Shallow	304	0.87
(shallow/deep)	ER	-327.2	656.3	0	Deep	168	0.13
Shallowest Depth (50m cut-off)	**ARD**	**-225.7**	**455.3**	**1**	Shallow	251	0.88
(shallow/deep)	ER	-327.5	656.9	0	Deep	221	0.12

**Traits and discrete character states**	**Evolutionary Model**				**Ancestral State at the Root**		
	**Model**	**Log(L)**	**AIC**	w_i	**Trait State**	**N**	**State Probability**
**B. Rajiformes (i.e., skates)**							
Conspicuous markings	**ARD**	**-138.7**	**281.3**	**0.98**	Absent	215	0.80
(present/absent)	ER	-143.7	289.3	0.02	Present	64	0.20
Conspicuous marking types	**ARD**	**-172.3**	**356.6**	**1**	No marking	215	0.64
(no marking/other marking/eyespot)	SYM	-181.9	369.9	0	Other marking	41	0.22
	ER	-193.7	389.5	0	Eyespot	23	0.14
Number of conspicuous markings	**ARD**	**-172.1**	**356.3**	**1**	None	250	0.80
(none, pair, >2)	SYM	-182.4	370.8	0	Pair	50	0.16
	ER	-195.6	393.3	0	>2	14	0.04
Mid-point Depth (200m cut-off)	**ARD**	**-177.9**	**359.8**	**0.65**	Shallow	144	0.32
(shallow/deep)	ER	-179.5	361.1	0.35	Deep	115	0.68
Mid-point Depth (150m cut-off)	**ARD**	**-179.5**	**361.1**	**0.73**	Shallow	130	ER: 0.50 / ARD: 0.50
(shallow/deep)	ER	**-179.5**	**363.0**	**0.26**	Deep	129	ER: 0.50 / ARD: 0.50
Mid-point Depth (100m cut-off)	**ARD**	**-175.2**	**354.5**	**0.96**	Shallow	106	0.07
(shallow/deep)	ER	-179.5	361.1	0.04	Deep	153	0.93
Mid-point Depth (50m cut-off)	**ARD**	**-136.5**	**277.1**	**1**	Shallow	70	0.31
(shallow/deep)	ER	-179.5	361.1	0	Deep	189	0.69
Shallowest Depth (200m cut-off)	**ARD**	**-177.9**	**359.8**	**0.65**	Shallow	144	0.32
(shallow/deep)	ER	-179.5	361.1	0.35	Deep	115	0.68
Shallowest Depth (150m cut-off)	**ER**	**-179.5**	**361.1**	**0.73**	Shallow	130	ER: 0.50 / ARD: 0.50
(shallow/deep)	**ARD**	**-179.5**	**363.0**	**0.26**	Deep	129	ER: 0.50 / ARD: 0.50
Shallowest Depth (100m cut-off)	**ARD**	**-175.2**	**354.5**	**0.96**	Shallow	106	0.41
(shallow/deep)	ER	-179.5	361.1	0.04	Deep	153	0.59
Shallowest Depth (50m cut-off)	**ARD**	**-136.5**	**277.1**	**1**	Shallow	70	0.30
(shallow/deep)	ER	-179.5	361.1	0	Deep	189	0.69

**Traits and discrete character states**	**Evolutionary Model**				**Ancestral State at the Root**		
	**Model**	**Log(L)**	**AIC**	***w_i***	**Trait State**	**N**	**State Probability**
**C. Rhinopristiformes, Torpediniformes, and Myliobatiformes (i.e., rays)**							
Conspicuous markings	**ARD**	**-59.9**	**123.8**	**0.93**	Absent	282	0.98
(present/absent)	ER	-63.5	129.0	0.07	Present	19	0.02
Conspicuous marking types	**ARD**	-63.0	138.0	0.95	No marking	282	0.99
(no marking/other marking/eyespot)	SYM	-69.1	144.3	0.04	Other marking	17	0.01
	ER	-72.7	147.4	0.01	Eyespot	2	0.007
Number of conspicuous markings	**ARD**	-65.5	143.0	0.91	None	282	0.98
(none, pair, >2)	SYM	-70.8	147.6	0.09	Pair	13	0.01
	ER	-77.2	156.5	0.001	>2	5	0.009
Defence mechanism	**ER**	**-46.6**	**95.2**	**0.72**	Standard	72	ER: 0.98 / ARD: 0.98
(standard/robust)	**ARD**	**-46.5**	**97.1**	**0.28**	Robust	228	ER: 0.02 / ARD: 0.02
Mid-point Depth (200m cut-off)	**ARD**	**-25.5**	**55.0**	**0.75**	Shallow	207	0.98
(shallow/deep)	ER	-27.6	57.2	0.25	Deep	6	0.02
Mid-point Depth (150m cut-off)	**ER**	-35.5	73.0	0.73	Shallow	203	ER: 1.0 / ARD: 0.97
(shallow/deep)	**ARD**	-35.5	75.0	0.27	Deep	10	ER: 0.0 / ARD: 0.03
Mid-point Depth (100m cut-off)	**ARD**	-49.0	102.0	0.93	Shallow	198	0.91
(shallow/deep)	ER	-52.5	107.1	0.07	Deep	15	0.09
Mid-point Depth (50m cut-off)	**ARD**	-78.5	161.1	0.92	Shallow	181	0.90
(shallow/deep)	ER	-81.9	165.8	0.08	Deep	32	0.10
Shallowest Depth (200m cut-off)	**ARD**	**-25.5**	**55.0**	**0.75**	Shallow	207	0.98
(shallow/deep)	ER	-27.6	57.2	0.25	Deep	6	0.02
Shallowest Depth (150m cut-off)	**ER**	-35.48	73.0	0.73	Shallow	203	ER: 1.0 / ARD: 0.97
(shallow/deep)	**ARD**	-35.47	75.0	0.27	Deep	10	ER: 0.0 / ARD: 0.03
Shallowest Depth (100m cut-off)	**ARD**	-49.0	102.0	0.93	Shallow	198	0.91
(shallow/deep)	ER	-52.5	107.1	0.07	Deep	15	0.09
Shallowest Depth (50m cut-off)	**ARD**	-78.5	161.1	0.92	Shallow	181	0.90
(shallow/deep)	ER	-81.9	165.8	0.08	Deep	32	0.10

Supplementary Table 2. **Phylogenetic logistic multiple regression models assessing if the presence/absence of conspicuous markings is predicted by defence mechanism (standard/robust), adult body length, and depth values in skates and rays.** Order-specific analyses using mid-point depth values are presented in **A-F**, while Batoid and Order-specific models using shallowest depth values are presented in **G-O**. Models compare Rajiformes (skates) with **A.** conspicuous markings (i.e., eyespots and other markings), **B.** eyespots, and **C.** other markings, **D.** Myliobatiformes (stingrays and their allies) with other markings (note that eyespots were not present in stingrays), and **E.** Torpediniformes (electric rays) with conspicuous markings (note that we combined eyespots and other markings as there were too few from each category for independent analyses), and **F.** Rhinopristiformes (shovelnose rays and their allies) with other markigns (note that eyespots were not present in shovelnose rays) against species lacking conspicuous markings. Similarly, models using shallowest depth values compare Batoids (skates and rays) with **G.** conspicuous markings (i.e., eyespots and other markings), **H.** eyespots, and **I.** other markings, Rajiformes (skates) with **J.** conspicuous markings (i.e., eyespots and other markings), **K.** eyespots, and **L.** other markings, **M.** Myliobatiformes (stingrays and their allies) with other markings (note that eyespots were not present in stingrays), and **N.** Torpediniformes (electric rays) with conspicuous markings (note that we combined eyespots and other markings as there were too few from each category for independent analyses), and **O.** Rhinopristiformes (shovelnose rays and their allies) with other markigns (note that eyespots were not present in shovelnose rays) against species lacking conspicuous markings. For each model, all possible interactions among predictors were included in initial models, When interaction terms had p values >0.10 they were removed from the model. Simplified models are presented in all cases, retaining only significant interaction terms. The sample size (n) reflects the number of species where data on all of the predictors was available. The number of species where conspicuous markings are present ($n_{present}$) and absent (n_{absent}), the correlation coefficient based on the likelihood of the fitted model (r^2_{lik}), the estimate, t-value and p-value are presented for each model.

Predictor	n_{total}	$n_{present}$	n_{absent}	r^2_{lik}	Estimate	z	p
			**Conspicuous markings**				
**A. Rajiformes (i.e., skates): conspicuous markings**							
Adult body length	250	58	192	0.30	-5.99	-1.23	0.22
Depth					-7.82	-2.17	**0.03**
Adult body length* Depth					3.11	1.64	*0.10*
**B. Rajiformes (i.e., skates): eyespots**							
Adult body length	214	22	192	0.43	-11.34	-3.19	**0.001**
Depth					-9.29	-3.47	**0.0005**
Adult body length* Depth					4.38	3.29	**0.001**
**C. Rajiformes (i.e., skates): other markings**							
Adult body length	228	36	192	0.19	0.89	1.62	0.30
Depth					-2.43	-4.83	**0.000001**
**D. Myliobatiformes (i.e., stingrays and their allies): other markings**							
Adult body length	116	7	109	0.17	-4.19	-2.38	**0.02**
Depth					-0.15	-0.17	0.86
**E. Torpediniformes (i.e., electric rays): conspicuous markings**							
Adult body length	51	4	47	-0.19	-8.93	-1.90	*0.06*
Depth					-5.44	-1.73	*0.08*
Adult body length* Depth					3.38	1.76	*0.07*
**F. Rhinopristiformes (i.e., shovelnose rays and their allies): other markings**							
Adult body length	46	7	39	0.77	-0.03	-0.03	0.98
Depth					0.04	0.07	0.95

			Conspicuous markings				
**Predictor**	n_{total}	$n_{present}$	n_{absent}	r^2_{lik}	**Estimate**	**t**	**p**
**G. Batoids (i.e., skates and rays): conspicuous markings**							
Defence mechanism	463	76	387	0.28	4.13	1.40	0.16
Adult body length					-2.17	-2.08	**0.04**
Shallowest Depth					-2.35	-2.15	**0.03**
Adult body length * Shallowest Depth					1.00	1.76	*0.08*
Adult body length * Defence mechanism					-3.57	-2.05	**0.04**
**H. Batoids (i.e., skates and rays): eyespots**							
Defence mechanism	411	24	387	0.30	-3.60	-2.46	**0.01**
Adult body length					-5.79	-2.64	**0.01**
Shallowest Depth					-5.62	-2.55	**0.01**
Body length * Shallowest Depth					2.68	2.47	**0.01**
**I. Batoids (i.e., skates and rays): other markings**							
Defence mechanism	439	52	387	0.16	-2.50	-3.39	**0.0007**
Adult body length					-0.67	-1.16	0.25
Shallowest Depth					-0.35	-1.40	0.16
**J. Rajiformes (i.e., skates): conspicuous markings**							
Adult body length	250	58	192	0.33	-3.86	-2.57	**0.01**
Shallowest Depth					-4.64	-3.09	**0.002**
Adult body length* Shallowest Depth					1.94	2.75	**0.006**
**K. Rajiformes (i.e., skates): eyespots**							
Adult body length	214	22	192	0.40	-4.15	-1.780	*0.08*
Shallowest Depth					-4.87	-2.157	**0.03**
Adult body length* Shallowest Depth					2.03	1.816	*0.07*
**L. Rajiformes (i.e., skates): other markings**							
Adult body length	228	36	192	0.23	-2.64	-2.33	**0.02**
Shallowest Depth					-3.13	-2.72	**0.007**
Adult body length* Shallowest Depth					1.31	2.47	**0.01**

**M. Myliobatiformes (i.e., stingrays and their allies): other markings**							
Adult body length	116	7	109	0.18	-4.31	-2.37	**0.02**
Shallowest Depth					-0.12	-0.22	0.83
**N. Torpediniformes (i.e., electric rays): conspicuous markings**							
Adult body length	51	4	47	-0.04	-3.74	-1.88	*0.06*
Shallowest Depth					-0.38	-0.95	0.34
**O. Rhinopristiformes (i.e., shovelnose rays and their allies): other markings**							
Adult body length	46	7	39	0.76	0.0005	0.001	1.0
Shallowest Depth					-0.0001	-0.001	1.0

Supplementary Table 3. Comparisons of model fit for discrete analysis assessing correlated evolution of the presence/absence of conspicuous markings with anti-predator defences (robust/not robust) and alternative depth cut-offs (shallow/deep) based on the mid-point depth where a species is found for A. Batoids (i.e., skates and rays) and B. Rajiformes (i.e., skates). In the independent model character states changed independently from one another. Three alternative dependent models were compared where the order of evolutionary transitions depending on one of the discrete characters, the other discrete character or on both discrete characters. We selected the best fitting dependent model by comparing AIC values. Models where AIC values were >2 units greater than AIC values from the best fitting dependent model were considered poorer fits. In cases where AIC values from multiple dependent models were within 2 units we compared multiple dependent models against the independent model. For each of the models the likelihood [Log(L)], degrees of freedom (df), Akaike Information Criterion (AIC), difference in AIC value between the best fitting model (Δ_i) and the AIC weight (w_i) are presented. We compared the Log(L) value from the independent model against the Log(L) value from the best fitting dependent model(s) and used likelihood ratio tests (LRT) to determine if the dependent model was a better fit than the independent model. The p value (p) for each model comparison is provided.

	Alternative Models					Model Comparison	
**Model**	**Log(L)**	**df**	**AIC**	**Δ_i**	**w_i**	**LRT**	**p**
**A. Batoids (i.e., skates and rays)**							
**i. Conspicuous markings vs. Anti-predator defence**							
Independent	-258.7	4	525.4	9.8	0		
Dependent: Conspicuous markings depend on anti-predator defences, but not vice versa	**-251.8**	**6**	**515.6**	**0**	**0.84**	**13.8**	**0.001**
Dependent: Anti-predator defences depend on conspicuous markings, but not vice versa	-257.9	6	527.8	12.2	0		
Dependent: Conspicuous markings depend on anti-predator defences, and vice versa	-251.5	8	519.0	3.4	0.15		
**ii. Eyespots vs. Anti-predator defence**							
Independent	-104.4	4	216.7	0.7	0.36		
Dependent: Eyespots depend on anti-predator defences, but not vice versa	-102.0	6	216.0	0	0.50	*4.70*	*0.095*
Dependent: Anti-predator defences depend on eyespots, but not vice versa	-104.1	6	220.3	4.3	0.06		
Dependent: Eyespots depend on anti-predator defences, and vice versa	-101.8	8	219.7	3.7	0.08		
**iii. Other markings vs. Anti-predator defence**							
Independent	-197.0	4	402.0	6.6	0.03		
Dependent: Conspicuous markings depend on anti-predator defences, but not vice versa	**-191.7**	**6**	**395.4**	**0**	**0.84**	**10.58**	**0.005**
Dependent: Anti-predator defences depend on conspicuous markings, but not vice versa	-196.4	6	404.8	9.4	0.008		
Dependent: Conspicuous markings depend on anti-predator defences, and vice versa	-191.6	8	399.3	3.9	0.12		
**ii. Conspicuous markings vs. Depth (200m cut-off)**							
Independent	-378.4	4	764.9	21.9	0		

Dependent: Conspicuous markings depend on depth, but not vice versa	-371.8	6	755.5	12.6	0		
Dependent: Depth depend on conspicuous markings, but not vice versa	-366.1	6	744.1	1.2	0.36		
Dependent: Conspicuous markings depend on depth, and vice versa	**-363.5**	**8**	**742.9**	**0**	**0.64**	**29.9**	**0.000005**
**iii. Conspicuous markings vs. Depth (150m cut-off)**							
Independent	-391.2	4	790.3	28.9	0		
Dependent: Conspicuous markings depend on depth, but not vice versa	-383.4	6	778.7	17.3	0		
Dependent: Depth depend on conspicuous markings, but not vice versa	-375.9	6	763.8	2.3	0.24		
Dependent: Conspicuous markings depend on depth, and vice versa	**-372.7**	**8**	**761.4**	**0**	**0.76**	**36.9**	**0.0000002**
**iv. Conspicuous markings vs. Depth (100m cut-off)**							
Independent	-404.0	4	816.1	36.1	0		
Dependent: Conspicuous markings depend on depth, but not vice versa	-394.7	6	801.4	21.4	0		
Dependent: Depth depend on conspicuous markings, but not vice versa	-386.3	6	784.5	4.5	0.09		
Dependent: Conspicuous markings depend on depth, and vice versa	**-382.0**	**8**	**780.0**	**0**	**0.91**	**44.09**	**0.000000006**
**v. Conspicuous markings vs. Depth (50m cut-off)**							
Independent	-412.8	4	833.6	27.5	0		
Dependent: Conspicuous markings depend on depth, but not vice versa	-406.5	6	825.0	18.9	0		
Dependent: Depth depend on conspicuous markings, but not vice versa	-402.4	6	816.8	10.7	0		
Dependent: Conspicuous markings depend on depth, and vice versa	**-395.1**	**8**	**806.1**	**0**	**1.00**	**35.5**	**0.0000004**

	Alternative Models					Model Comparison	
**Model**	**Log(L)**	**df**	**AIC**	**Δ_i**	**w_i**	**Log(L)**	**p**
**B. Rajiformes (skates)**							
**i. Conspicuous markings vs. Depth (200m cut-off)**							
Independent	-286.2	4	580.4	44.5	0		
Dependent: Conspicuous markings depend on depth, but not vice versa	-262.6	6	537.1	1.3	0.34	**47.3**	**0.00000000001**
Dependent: Depth depend on conspicuous markings, but not vice versa	-265.8	6	543.5	7.7	0.01		
Dependent: Conspicuous markings depend on depth, and vice versa	**-259.9**	**6**	**535.8**	**0**	**0.65**	**52.5**	**0.00000000001**
**ii. Conspicuous markings vs. Depth (150m cut-off)**							
Independent	-288.8	4	585.7	49.1	0		
Dependent: Conspicuous markings depend on depth, but not vice versa	**-262.3**	**6**	**536.5**	**0**	**0.61**	**53.1**	**0.000000000003**
Dependent: Depth depend on conspicuous markings, but not vice versa	-265.3	6	542.6	6.07	0.03		
Dependent: Conspicuous markings depend on depth, and vice versa	-260.8	8	537.5	1.01	0.37	**56.1**	**0.00000000002**
**iii. Conspicuous markings vs. Depth (100m cut-off)**							
Independent	-284.7	4	577.4	51.2	0		
Dependent: Conspicuous markings depend on depth, but not vice versa	-257.2	6	526.4	0.2	0.37	**55.0**	**0.000000000001**
Dependent: Depth depend on conspicuous markings, but not vice versa	-257.8	6	527.5	1.3	0.21	**53.9**	**0.000000000002**
Dependent: Conspicuous markings depend on depth, and vice versa	**-255.1**	**8**	**526.2**	**0**	**0.42**	**59.2**	**0.000000000004**
**iv. Conspicuous markings vs. Depth (50m cut-off)**							
Independent	-261.9	4	531.8	22.8	0		

Dependent: Conspicuous markings depend on depth, but not vice versa	**-248.5**	**6**	**509.0**	**0**	**0.46**	**26.8**	**0.000002**
Dependent: Depth depend on conspicuous markings, but not vice versa	-248.6	6	509.2	0.2	0.41	**26.6**	**0.000002**
Dependent: Conspicuous markings depend on depth, and vice versa	-247.7	8	511.4	2.44	0.14		

Supplemental Table 4. List of websites used to find images of skates and rays to compare the presence/absence of conspicuous markings from photographs to those obtained from the drawing in Last et al. (2016)*. Species names, hyperlinks to websites where photographs were assessed and the date when the website was access are provided.

* Last, P. R., White, W.T., de Carvalho, M.R., Séret, B., Stehmann, M.F.W., and Naylor, G.J.P. (2016). *Rays of the World*. CSIRO Publishing.

**Species**	**Hyperlink to photo source**	**Date when accessed**
Pseudobatos_lentiginosus	http://shark-references.com/images/species/R.blochii.jpg	2025-06-11
Acroteriobatus_salalah	http://fishbase.se/images/species/Rhsal_u0.jpg	2025-06-11
Acroteriobatus_variegatus	http://fishbase.se/images/species/Rhlio_u0.jpg	2025-06-11
Aetobatus_flagellum	http://www.fishbase.se/images/species/Aefla_u1.jpg	2025-06-11
Aetobatus_flagellum	http://www.fishbase.se/photos/workimagethumb.php?s=http://www.fishbase.se/tools/UploadPhoto/uploads/1376020909_182.178.44.103.jpg&w=600	2025-06-11
Aetobatus_flagellum	http://www.fishbase.se/photos/workimagethumb.php?s=http://www.fishbase.se/tools/UploadPhoto/uploads/1379835334_182.178.17.12.jpg&w=600	2025-06-11
Aetobatus_laticeps	https://www.fishbase.se/images/species/Aenar_u5.jpg	2025-06-11
Aetomylaeus_milvus	https://www.inaturalist.org/photos/119950567	2025-06-11
Aetobatus_narinari	http://www.fishbase.se/images/species/Aenar_u1.jpg	2025-06-11
Aetobatus_narinari	http://www.fishbase.se/images/species/Aenar_u5.jpg	2025-06-11
Aetobatus_narinari	http://www.fishbase.se/images/species/Aenar_u7.jpg	2025-06-11
Aetobatus_narinari	http://www.fishbase.se/images/species/Aenar_u8.jpg	2025-06-11
Aetobatus_narinari	https://www.fishbase.se/images/species/Aenar_u4.jpg	2025-06-11
Aetobatus_narinari	https://static.inaturalist.org/photos/6612557/medium.jpeg?1489799299	2025-06-11
Aetobatus_narutobiei	https://www.fishbase.se/photos/PicturesSummary.php?resultPage=2&ID=67276&what=species	2025-06-11
Aetobatus_narutobiei	https://www.fishbase.se/photos/PicturesSummary.php?resultPage=1&ID=67276&what=species	2025-06-11
Aetobatus_ocellatus	https://shark-references.com/images/species/A_ocellatus_1.jpg	2025-06-11
Aetobatus_ocellatus	https://www.fishbase.se/images/species/Aenar_u1.jpg	2025-06-11
Aetobatus_ocellatus	https://www.fishbase.se/images/species/Aenar_u7.jpg	2025-06-11
Aetobatus_ocellatus	https://www.fishbase.se/images/species/Aenar_u3.jpg	2025-06-11
Aetomylaeus_bovinus	http://www.fishbase.se/images/species/Myaqu_u0.jpg	2025-06-11
Aetomylaeus_bovinus	http://www.fishbase.se/images/species/Ptbov_u1.jpg	2025-06-11
Aetomylaeus_bovinus	http://www.fishbase.se/tools/UploadPhoto/uploads/pterom_bov.jpg	2025-06-11
Aetomylaeus_caeruleofasciatus	http://shark-references.com/images/species/Aetomylaeus_caeruleofasciatus_first.jpg	2025-06-11
Aetomylaeus_caeruleofasciatus	http://shark-references.com/images/species/Aetomylaeus_caeruleofasciatus_fresh.jpg	2025-06-11
Aetomylaeus_caeruleofasciatus	http://shark-references.com/images/species/Aetomylaeus_caeruleofasciatus_main.jpg	2025-06-11
Aetomylaeus_nichofii	http://www.boldsystems.org/pics/SABF/0025_Aetomylaeus_nichofii%2B1333309126.jpg	2025-06-11
Aetomylaeus_nichofii	http://www.boldsystems.org/pics/SABF/0133_Aetomylaeus_nichofii%2B1339610384.JPG	2025-06-11
Aetomylaeus_nichofii	http://www.fishbase.se/images/species/Aenic_u0.jpg	2025-06-11
Aetomylaeus_nichofii	http://www.fishbase.se/images/species/Aenic_u1.jpg	2025-06-11

Aetomylaeus_nichofii	http://www.fishbase.se/images/species/Aenic_u3.jpg	2025-06-11
Aetomylaeus_nichofii	http://www.fishbase.se/photos/workimagethumb.php?s=http://www.fishbase.se/tools/UploadPhoto/uploads/1430327266_39.44.187.174.jpg&w=600	2025-06-11
Aetomylaeus_nichofii	http://www.fishbase.se/photos/workimagethumb.php?s=http://www.fishbase.se/tools/UploadPhoto/uploads/1430327634_39.44.187.174.jpg&w=600	2025-06-11
Aetomylaeus_nichofii	http://www.fishbase.se/tools/UploadPhoto/uploads/1365693725_182.178.97.108.jpg	2025-06-11
Aetomylaeus_vespertilio	http://fishesofaustralia.net.au/Images/Image/AetomylaeusVespertilioCSIRO.jpg	2025-06-11
Aetomylaeus_vespertilio	http://fishesofaustralia.net.au/images/image/AetomylaeusVespertilioWW.jpg	2025-06-11
Aetomylaeus_vespertilio	http://www.fishbase.se/FishWatcher/uploads/images/DSC00910%20for%20fishbase%20Aetomylaeus%20vespertilio.jpg	2025-06-11
Aetomylaeus_vespertilio	http://www.fishbase.se/tools/UploadPhoto/uploads/Aetomylaeus_vespertilio.jpg	2025-06-11
Aetomylaeus_vespertilio	http://www.fishbase.se/tools/UploadPhoto/uploads/Photo0043.jpg	2025-06-11
Amblyraja_doellojuradoi	http://www.boldsystems.org/pics/FARG/INIDEP-DI_0395_1%2B1196764906.jpg	2025-06-11
Amblyraja_doellojuradoi	http://www.boldsystems.org/pics/FARG/INIDEP-DI_0396_1%2B1196764530.jpg	2025-06-11
Amblyraja_doellojuradoi	http://www.boldsystems.org/pics/FARG/INIDEP-DI_0496%2B1196252122.JPG	2025-06-11
Amblyraja_doellojuradoi	http://www.boldsystems.org/pics/FARG/INIDEP-T_0130%2B1139951786.JPG	2025-06-11
Amblyraja_doellojuradoi	http://www.boldsystems.org/pics/FARG/INIDEP-T_0171%2B1141039710.JPG	2025-06-11
Amblyraja_doellojuradoi	http://www.boldsystems.org/pics/FARG/INIDEP-T_0186%2B1141058624.JPG	2025-06-11
Amblyraja_doellojuradoi	http://www.boldsystems.org/pics/FARG/INIDEP-T_0242%2B1139505654.jpg	2025-06-11
Amblyraja_doellojuradoi	http://www.fishbase.se/images/species/Amdoe_f0.jpg	2025-06-11
Amblyraja_doellojuradoi	http://www.fishbase.se/images/species/Amdoe_m1.jpg	2025-06-11
Amblyraja_frerichsi	http://shark-references.com/images/species/Amblyraja_frerichsi.jpg	2025-06-11
Amblyraja_frerichsi	http://www.fishbase.se/images/species/Amfre_ua.jpg	2025-06-11
Amblyraja_georgiana	http://www.boldsystems.org/pics/FARAN/UNMDP_DI_3279%2B1415647814.JPG	2025-06-11
Amblyraja_georgiana	http://www.boldsystems.org/pics/FARAN/UNMDP_DI_3280%2B1415647838.JPG	2025-06-11
Amblyraja_georgiana	http://www.fishbase.se/images/species/Amgeo_u1.jpg	2025-06-11
Amblyraja_georgiana	http://www.fishbase.se/tools/UploadPhoto/uploads/amblyrajageorgiana.jpg	2025-06-11
Amblyraja_hyperborea	http://fishesofaustralia.net.au/images/image/AmblyrajaCSIRO.jpg	2025-06-11
Amblyraja_hyperborea	http://www.boldsystems.org/pics/RNEZ/RNEZ178D%2B1265229796.jpg	2025-06-11
Amblyraja_hyperborea	http://www.boldsystems.org/pics/SCAFB/23PA8a_Arctic__M_running_%5B80cm%5D%2B1168972306.JPG	2025-06-11
Amblyraja_hyperborea	http://www.boldsystems.org/pics/SCAFB/23PA8b_Arctic_M_juvenile_%5B57cm%5D%2B1168963992.JPG	2025-06-11
Amblyraja_hyperborea	http://www.boldsystems.org/pics/SCAFB/23PA8e_Arctic__M_adolescent_%5B56cm%5D%2B1168971054.JPG	2025-06-11
Amblyraja_hyperborea	http://www.boldsystems.org/pics/SCAFB/25PA8a_Arctic_M_juvenile_%5B42cm%5D%2B1168533788.JPG	2025-06-11
Amblyraja_hyperborea	http://www.fishbase.se/images/species/Amhyp_u1.jpg	2025-06-11
Amblyraja_hyperborea	http://www.fishbase.se/tools/UploadPhoto/uploads/82132_2a.jpg	2025-06-11
Amblyraja_jenseni	http://www.boldsystems.org/pics/SCFAC/OBS386_Jensens_M_running_%5B105cm%5D%2B1160146486.JPG	2025-06-11
Amblyraja_jenseni	http://www.fishbase.se/images/species/Amjen_f0.jpg	2025-06-11
Amblyraja_jenseni	http://www.fishbase.se/images/species/Amjen_j0.jpg	2025-06-11
Amblyraja_jenseni	http://www.fishbase.se/images/species/Amjen_m1.jpg	2025-06-11
Amblyraja_jenseni	http://www.fishbase.se/images/species/Amjen_u0.jpg	2025-06-11
Amblyraja_radiata	http://shark-references.com/images/species/thumbnail/BKT80007_6a.jpg	2025-06-11
Amblyraja_radiata	http://shark-references.com/images/species/thumbnail/BKT80029_4a.jpg	2025-06-11
Amblyraja_radiata	http://www.boldsystems.org/pics/BNSF/MT02934%2B1326224566.jpg	2025-06-11
Amblyraja_radiata	http://www.boldsystems.org/pics/BNSF/MT03003%2B1326994816.jpg	2025-06-11
Amblyraja_radiata	http://www.boldsystems.org/pics/SCAFB/06-1078%2B1182260300.JPG	2025-06-11
Amblyraja_radiata	http://www.fishbase.se/images/species/Amrad_u0.jpg	2025-06-11

Amblyraja_radiata	http://www.fishbase.se/images/species/Amrad_u1.jpg	2025-06-11
Amblyraja_radiata	http://www.fishbase.se/tools/UploadPhoto/uploads/STARRYRAY.jpg	2025-06-11
Anoxypristis_cuspidata	http://3.bp.blogspot.com/-ouD1hT7ceHw/VI9ULKl-8TI/AAAAAAAAAG4/pUFqAYSNnLw/s1600/Capture.PNG	2025-06-11
Anoxypristis_cuspidata	http://fishesofaustralia.net.au/images/image/AnoxypristisCuspidatCSIRO.jpg	2025-06-11
Aptychotrema_rostrata	http://fishesofaustralia.net.au/images/image/AptychotRostratErikSchlogl.jpg	2025-06-11
Aptychotrema_rostrata	http://shark-references.com/images/species/Aptychotrema_rostrata_sydney.jpg	2025-06-11
Aptychotrema_rostrata	http://www.fishbase.se/images/species/Apros_f0.jpg	2025-06-11
Aptychotrema_rostrata	http://www.fishbase.se/images/species/Apros_j0.jpg	2025-06-11
Aptychotrema_rostrata	http://www.fishbase.se/images/species/Apros_u1.jpg	2025-06-11
Aptychotrema_rostrata	http://www.fishbase.se/tools/UploadPhoto/uploads/DSC00686.JPG	2025-06-11
Aptychotrema_timorensis	http://fishesofaustralia.net.au/images/image/Aptychotrematimorensis.jpg	2025-06-11
Aptychotrema_vincentiana	http://fishesofaustralia.net.au/images/image/Aptychotrema-vincentiana_RKhero.jpg	2025-06-11
Atlantoraja_castelnaui	https://www.fishbase.se/images/species/Atcas_u3.jpg	2025-06-11
Atlantoraja_castelnaui	http://www.boldsystems.org/pics/FARG/INIDEP-DI_0406%2B1195143796.jpg	2025-06-11
Atlantoraja_castelnaui	http://www.boldsystems.org/pics/FARGB/UNMDP_DI_0195%2B1305843202.jpg	2025-06-11
Atlantoraja_castelnaui	http://www.boldsystems.org/pics/FARGB/UNMDP_DI_0196%2B1305843210.jpg	2025-06-11
Atlantoraja_castelnaui	http://www.boldsystems.org/pics/RAJBR/movi2051-d%2B1190304948.jpg	2025-06-11
Atlantoraja_castelnaui	http://www.boldsystems.org/pics/RAJBR/movi2386-d%2B1190047358.jpg	2025-06-11
Atlantoraja_castelnaui	http://www.boldsystems.org/pics/RAJBR/rni049-d%2B1191193830.jpg	2025-06-11
Atlantoraja_castelnaui	http://www.fishbase.se/images/species/Atcas_f0.jpg	2025-06-11
Atlantoraja_castelnaui	http://www.fishbase.se/images/species/Atcas_u0.jpg	2025-06-11
Atlantoraja_castelnaui	http://www.fishbase.se/images/species/Atcas_u1.jpg	2025-06-11
Atlantoraja_castelnaui	http://www.fishbase.se/images/species/Atcas_u3.jpg	2025-06-11
Atlantoraja_castelnaui	http://www.fishbase.se/images/species/Atcas_u4.jpg	2025-06-11
Atlantoraja_castelnaui	http://www.fishbase.se/images/species/Atcas_u5.jpg	2025-06-11
Atlantoraja_cyclophora	http://shark-references.com/images/species/Atlantoraja_cyclophora-main.jpg	2025-06-11
Atlantoraja_cyclophora	http://www.boldsystems.org/pics/FARG/INIDEP-DI_0471%2B1196161206.JPG	2025-06-11
Atlantoraja_cyclophora	http://www.boldsystems.org/pics/FARG/INIDEP-DI_0474%2B1196161628.JPG	2025-06-11
Atlantoraja_cyclophora	http://www.boldsystems.org/pics/FARGB/UNMDP-DI_001%2B1312408236.JPG	2025-06-11
Atlantoraja_cyclophora	http://www.boldsystems.org/pics/RAJBR/movi2010-d%2B1189876882.jpg	2025-06-11
Atlantoraja_cyclophora	https://www.fishbase.se/images/species/Atcyc_u3.jpg	2025-06-11
Atlantoraja_cyclophora	http://www.boldsystems.org/pics/RAJBR/movi2166-d%2B1190417636.jpg	2025-06-11
Atlantoraja_cyclophora	http://www.fishbase.se/images/species/Atcyc_j0.jpg	2025-06-11
Atlantoraja_cyclophora	http://www.fishbase.se/images/species/Atcyc_u0.jpg	2025-06-11
Atlantoraja_cyclophora	http://www.fishbase.se/images/species/Atcyc_u1.jpg	2025-06-11
Atlantoraja_cyclophora	http://www.fishbase.se/images/species/Atcyc_u3.jpg	2025-06-11
Atlantoraja_cyclophora	http://www.fishbase.se/tools/UploadPhoto/uploads/CEPSUL_At_cyc.jpg	2025-06-11
Atlantoraja_platana	http://shark-references.com/images/species/Atlantoraja_platana_by_Coller_Nidia_Marina_Argentina.jpg	2025-06-11
Atlantoraja_platana	http://www.boldsystems.org/pics/FARG/INIDEP-DI_0425%2B1195565558.jpg	2025-06-11
Atlantoraja_platana	http://www.boldsystems.org/pics/FARG/INIDEP-DI_0426%2B1195726370.jpg	2025-06-11
Atlantoraja_platana	http://www.boldsystems.org/pics/RAJBR/movi2002-d%2B1189958232.jpg	2025-06-11
Atlantoraja_platana	http://www.boldsystems.org/pics/RAJBR/movi2014-d%2B1189881120.jpg	2025-06-11
Atlantoraja_platana	http://www.boldsystems.org/pics/RAJBR/movi2111-d%2B1226895116.jpg	2025-06-11

Atlantoraja_platana	http://www.boldsystems.org/pics/RAJBR/movi2113-d%2B1190305760.jpg	2025-06-11
Atlantoraja_platana	http://www.boldsystems.org/pics/RAJBR/movi2175-d%2B1226894900.jpg	2025-06-11
Atlantoraja_platana	http://www.fishbase.se/images/species/Atpla_f0.jpg	2025-06-11
Atlantoraja_platana	http://www.fishbase.se/images/species/Atpla_j0.jpg	2025-06-11
Bathyraja_abyssicola	http://www.boldsystems.org/pics/TZFPB/Bathyraja_abyssicola_-_deepsea_skate_Tow_14_sample_311_004.JPG	2025-06-11
Bathyraja_abyssicola	http://www.boldsystems.org/pics/TZFPB/Bathyraja_abyssicola_-_deepsea_skate_Tow_29_sample_421_022.JPG	2025-06-11
Bathyraja_albomaculata	http://shark-references.com/images/species/B%20albomaculata.jpg	2025-06-11
Bathyraja_albomaculata	http://www.boldsystems.org/pics/FARG/INIDEP-T_0101%2B1140297094.jpg	2025-06-11
Bathyraja_albomaculata	http://www.boldsystems.org/pics/FARG/INIDEP-T_0141%2B1140355852.jpg	2025-06-11
Bathyraja_albomaculata	http://www.boldsystems.org/pics/FARG/INIDEP-T_0143%2B1140347224.jpg	2025-06-11
Bathyraja_albomaculata	http://www.boldsystems.org/pics/FARG/INIDEP-T_0145%2B1139506080.jpg	2025-06-11
Bathyraja_albomaculata	http://www.boldsystems.org/pics/FARGB/UNMDP_DI_0192%2B1305843176.jpg	2025-06-11
Bathyraja_aleutica	http://www.fishbase.se/images/species/Baale_u0.jpg	2025-06-11
Bathyraja_aleutica	https://inaturalist-open-data.s3.amazonaws.com/photos/1651138/medium.jpg	2025-06-11
Bathyraja_brachyurops	http://www.boldsystems.org/pics/CEGAR/CEGAR_DI_107b%2B1357763494.JPG	2025-06-11
Bathyraja_brachyurops	http://www.boldsystems.org/pics/FARG/INIDEP-T_0235%2B1139504536.jpg	2025-06-11
Bathyraja_brachyurops	http://www.boldsystems.org/pics/FARG/INIDEP-T_0244%2B1139506210.jpg	2025-06-11
Bathyraja_brachyurops	http://www.boldsystems.org/pics/FARG/INIDEP-T_0245%2B1139506392.jpg	2025-06-11
Bathyraja_brachyurops	https://inaturalist-open-data.s3.amazonaws.com/photos/11424063/medium.jpeg	2025-06-11
Bathyraja_brachyurops	http://www.fishbase.se/images/species/Babra_m0.jpg	2025-06-11
Bathyraja_cousseauae	http://www.boldsystems.org/pics/FARG/INIDEP-T_0172%2B1140983796.jpg	2025-06-11
Bathyraja_cousseauae	http://www.boldsystems.org/pics/FARG/INIDEP-T_0181%2B1141126056.jpg	2025-06-11
Bathyraja_cousseauae	http://www.boldsystems.org/pics/FARGB/UNMDP_DI_4519%2B1459339638.JPG	2025-06-11
Bathyraja_cousseauae	http://www.fishbase.se/images/species/Bacou_f0.jpg	2025-06-11
Bathyraja_cousseauae	http://www.fishbase.se/images/species/Bacou_m0.jpg	2025-06-11
Bathyraja_eatonii	http://www.boldsystems.org/pics/FARAN/UNMDP_DI_3274%2B1415647706.JPG	2025-06-11
Bathyraja_eatonii	http://www.boldsystems.org/pics/FARAN/UNMDP_DI_3278%2B1415647770.JPG	2025-06-11
Bathyraja_griseocauda	http://shark-references.com/images/species/DSC00073.jpg	2025-06-11
Bathyraja_griseocauda	http://www.boldsystems.org/pics/FARG/INIDEP-T_0153%2B1140997142.JPG	2025-06-11
Bathyraja_griseocauda	http://www.boldsystems.org/pics/FARG/INIDEP-T_0155%2B1140968240.JPG	2025-06-11
Bathyraja_griseocauda	http://www.boldsystems.org/pics/FARG/INIDEP-T_0156%2B1140968390.JPG	2025-06-11
Bathyraja_griseocauda	http://www.boldsystems.org/pics/FARG/INIDEP-T_0161%2B1141034194.JPG	2025-06-11
Bathyraja_interrupta	http://media.eol.org/content/2011/12/14/01/67209_580_360.jpg	2025-06-11
Bathyraja_interrupta	http://www.boldsystems.org/pics/FMV/UW047705%2B1052154964.JPG	2025-06-11
Bathyraja_interrupta	http://www.boldsystems.org/pics/FMV/UW047720%2B1052154968.JPG	2025-06-11
Bathyraja_interrupta	http://www.boldsystems.org/pics/TZFPB/Bathyraja_interrupta_-_sandpaper_skate_Tow_10_sample_242_012.JPG	2025-06-11
Bathyraja_interrupta	http://www.boldsystems.org/pics/TZFPB/Bathyraja_interrupta_-_sandpaper_sole_Tow_15_sample_320_002.JPG	2025-06-11
Bathyraja_interrupta	http://www.boldsystems.org/pics/TZFPB/Bathyraja_interrupta_-_sandpaper_sole_Tow_15_sample_320_002.JPG	2025-06-11
Bathyraja_interrupta	http://www.boldsystems.org/pics/TZFPB/IMGP1011%2B1060292348.JPG	2025-06-11
Bathyraja_interrupta	http://www.boldsystems.org/pics/TZFPB/IMGP1013%2B1060292634.JPG	2025-06-11
Bathyraja_interrupta	http://www.boldsystems.org/pics/TZFPB/IMGP1015%2B1060292808.JPG	2025-06-11
Bathyraja_interrupta	http://www.fishbase.se/images/species/Baint_u1.jpg	2025-06-11
Bathyraja_isotrachys	http://www.fishbase.se/images/species/Baiso_u0.jpg	2025-06-11

Bathyraja_leucomelanos	http://www.fishbase.se/images/species/Baleu_u2.jpg	2025-06-11
Bathyraja_maccaini	http://www.boldsystems.org/pics/FWFA/UNMDP_DI_2996.2%2B1372159678.JPG	2025-06-11
Bathyraja_maccaini	http://www.fishbase.se/images/species/Bamac_j0.jpg	2025-06-11
Bathyraja_macloviana	https://www.fishbase.se/images/species/Bamac_m0.jpg	2025-06-11
Bathyraja_maculata	https://fishbase.se/images/species/Bamac_u6.jpg	2025-06-11
Bathyraja_magellanica	https://v3.boldsystems.org/pics/_w300/FARG/INIDEP-T_0148%2B1140625590.jpg	2025-06-11
Bathyraja_magellanica	https://v3.boldsystems.org/pics/_w300/FARG/INIDEP-T_0129%2B1140009128.jpg	2025-06-11
Bathyraja_magellanica	https://v3.boldsystems.org/pics/_w300/FARG/INIDEP-T_0147%2B1140453648.jpg	2025-06-11
Bathyraja_mariposa	https://apps-afsc.fisheries.noaa.gov/Quarterly/jfm2005/images/skateFig4.jpg	2025-06-11
Bathyraja_matsubarai	https://www.fishbase.se/images/species/Bamat_u0.jpg	2025-06-11
Bathyraja_meridionalis	https://www.fishbase.se/images/species/Bamer_m0.jpg	2025-06-11
Bathyraja_meridionalis	https://www.fishbase.se/images/species/Bamer_m1.jpg	2025-06-11
Bathyraja_meridionalis	https://www.fishbase.se/images/species/Bamer_u4.jpg	2025-06-11
Bathyraja_meridionalis	https://www.fishbase.se/images/species/Bamer_u5.jpg	2025-06-11
Bathyraja_microtrachys	https://media.fisheries.noaa.gov/dam-migration-miss/1280_ADti5stnQX98.jpg?1564685071	2025-06-11
Bathyraja_microtrachys	https://biogeodb.stri.si.edu/sftep/resources/img/images/species/5962_7967.jpg	2025-06-11
Bathyraja_minispinosa	https://www.fishbase.se/images/species/Bamin_u0.jpg	2025-06-11
Bathyraja_multispinis	https://fishbase.se/images/species/Bamul_j0.jpg	2025-06-11
Bathyraja_multispinis	https://fishbase.se/images/species/Bamul_m0.jpg	2025-06-11
Bathyraja_multispinis	https://v3.boldsystems.org/pics/FARG/INIDEP-T_0154%2B1140997456.jpg	2025-06-11
Bathyraja_multispinis	https://v3.boldsystems.org/pics/_w300/FARG/INIDEP-T_0154%2B1140997456.jpg	2025-06-11
Bathyraja_multispinis	https://v3.boldsystems.org/pics/_w300/FARG/INIDEP-T_0150%2B1140909648.jpg	2025-06-11
Bathyraja_multispinis	https://v3.boldsystems.org/pics/_w300/FARG/INIDEP-DI_0479%2B1196159604.JPG	2025-06-11
Bathyraja_murrayi	https://inaturalist-open-data.s3.amazonaws.com/photos/6797071/medium.jpeg	2025-06-11
Bathyraja_murrayi	https://inaturalist-open-data.s3.amazonaws.com/photos/6796992/medium.jpeg	2025-06-11
Bathyraja_murrayi	https://inaturalist-open-data.s3.amazonaws.com/photos/6796917/large.jpeg	2025-06-11
Bathyraja_murrayi	https://inaturalist-open-data.s3.amazonaws.com/photos/6796825/large.jpeg	2025-06-11
Bathyraja_murrayi	https://inaturalist-open-data.s3.amazonaws.com/photos/6797071/medium.jpeg	2025-06-11
Bathyraja_pacifica	http://shark-references.com/images/species/Bathyraja_pacifica_holotyp.jpg	2025-06-11
Bathyraja_pallida	http://www.fishbase.se/images/species/Bapal_u6.jpg	2025-06-11
Bathyraja_papilionifera	http://www.boldsystems.org/pics/FARG/INIDEP-DI_0500%2B1198677452.jpg	2025-06-11
Bathyraja_parmifera	http://www.boldsystems.org/pics/DSFAL/P7241070%2B1224268444.JPG	2025-06-11
Bathyraja_parmifera	http://www.fishbase.se/images/species/Bapar_u0.jpg	2025-06-11
Bathyraja_peruana	http://www.fishbase.se/images/species/Baper_m0.jpg	2025-06-11
Bathyraja_peruana	http://www.fishbase.se/images/species/Baper_m2.jpg	2025-06-11
Bathyraja_richardsoni	http://fishesofaustralia.net.au/Images/Image/BathyrajaRichardsoniCSIRO.jpg	2025-06-11
Bathyraja_richardsoni	http://fishesofaustralia.net.au/images/image/BathyrajRichards2CSIRO.jpg	2025-06-11
Bathyraja_richardsoni	http://fishesofaustralia.net.au/Images/Image/BathyrajRichardsSERPENT.jpg	2025-06-11
Bathyraja_richardsoni	http://www.fishbase.se/images/species/Baric_u1.jpg	2025-06-11
Bathyraja_scaphiops	http://www.boldsystems.org/pics/FARG/INIDEP-DI_0466%2B1196158834.JPG	2025-06-11
Bathyraja_scaphiops	http://www.boldsystems.org/pics/FARG/INIDEP-T_0097%2B1139505392.jpg	2025-06-11
Bathyraja_scaphiops	http://www.boldsystems.org/pics/FARG/INIDEP-T_0098%2B1139505276.jpg	2025-06-11
Bathyraja_scaphiops	http://www.boldsystems.org/pics/FARG/INIDEP-T_0099%2B1139505202.jpg	2025-06-11

Bathyraja_scaphiops	http://www.boldsystems.org/pics/FARG/INIDEP-T_0114%2B1139570886.jpg	2025-06-11
Bathyraja_scaphiops	http://www.boldsystems.org/pics/FARGB/UNMDP_DI_0191%2B1305843168.jpg	2025-06-11
Bathyraja_scaphiops	http://www.fishbase.se/images/species/Basca_f0.jpg	2025-06-11
Bathyraja_scaphiops	http://www.fishbase.se/images/species/Basca_f1.jpg	2025-06-11
Bathyraja_schroederi	http://www.fishbase.se/images/species/Basch_f0.jpg	2025-06-11
Bathyraja_smithii	http://www.boldsystems.org/pics/HVDB/IMG_1311%2B324668050.JPG	2025-06-11
Bathyraja_smithii	http://www.fishbase.se/images/species/Basmi_u0.jpg	2025-06-11
Bathyraja_smithii	http://www.fishbase.se/images/species/Basmi_u2.jpg	2025-06-11
Bathyraja_spinicauda	http://shark-references.com/images/species/82128_1a.jpg	2025-06-11
Bathyraja_spinicauda	http://shark-references.com/images/species/BKT80030_4.jpg	2025-06-11
Bathyraja_spinicauda	http://shark-references.com/images/species/BKT80030_4a.jpg	2025-06-11
Bathyraja_spinicauda	http://www.boldsystems.org/pics/RNEZ/RNEZ134D%2B1265400706.jpg	2025-06-11
Bathyraja_spinicauda	http://www.boldsystems.org/pics/RNEZ/RNEZ135D%2B1265138890.jpg	2025-06-11
Bathyraja_spinicauda	http://www.boldsystems.org/pics/RNEZ/RNEZ139D%2B1265401074.jpg	2025-06-11
Bathyraja_spinicauda	http://www.boldsystems.org/pics/RNEZ/RNEZ255D%2B1265150512.jpg	2025-06-11
Bathyraja_spinicauda	http://www.fishbase.se/images/species/Baspi_u0.jpg	2025-06-11
Bathyraja_spinosissima	http://www.boldsystems.org/pics/ELAME/d000835%2B1260411082.JPG	2025-06-11
Bathyraja_spinosissima	http://www.boldsystems.org/pics/ELAME/d000836%2B1260411078.JPG	2025-06-11
Bathyraja_spinosissima	http://www.boldsystems.org/pics/ELAME/d000837%2B1260411082.JPG	2025-06-11
Bathyraja_spinosissima	http://www.boldsystems.org/pics/ELAME/d000838%2B1260411096.JPG	2025-06-11
Bathyraja_taranetzi	http://www.fishbase.se/images/species/Rhtar_u0.jpg	2025-06-11
Bathyraja_trachura	http://www.boldsystems.org/pics/FMV/UW_151521%2B1380226986.JPG	2025-06-11
Bathyraja_trachura	http://www.boldsystems.org/pics/TZFPB/Bathyraja_trachura_-_roughtail_skate_Tow_11_sample_270_042.JPG	2025-06-11
Bathyraja_trachura	http://www.boldsystems.org/pics/TZFPB/Bathyraja_trachura_-_roughtail_skate_Tow_13_sample_303_076.JPG	2025-06-11
Bathyraja_trachura	http://www.boldsystems.org/pics/TZFPB/Bathyraja_trachura_-_roughtail_skate_Tow_13_sample_304_077.JPG	2025-06-11
Bathyraja_trachura	http://www.boldsystems.org/pics/TZFPB/Bathyraja_trachura_-_roughtail_skate_Tow_13_sample_332_020.JPG	2025-06-11
Bathyraja_trachura	http://www.boldsystems.org/pics/TZFPB/Bathyraja_trachura_-_roughtail_skate_Tow_13_sample_333_021.JPG	2025-06-11
Bathyraja_trachura	http://www.boldsystems.org/pics/TZFPC/IMG_1799%2B1160702154.JPG	2025-06-11
Bathyraja_trachura	http://www.fishbase.se/images/species/Batra_u0.jpg	2025-06-11
Bathyraja_trachura	http://www.fishbase.se/images/species/Batra_u2.jpg	2025-06-11
Bathyraja_violacea	http://www.boldsystems.org/pics/TZFPB/Lycodapus_pachysoma_-_stout_eelpout_Tow_20_sample_352_024.JPG	2025-06-11
Bathyraja_violacea	https://v3.boldsystems.org/pics/TZFPC/IMG_1830%2B1160775264.JPG	2025-06-11
Bathyraja_violacea	http://www.boldsystems.org/pics/TZFPB/Lycodapus_pachysoma_-_stout_eelpout_Tow_20_sample_353_026.JPG	2025-06-11
Bathyraja_violacea	http://www.boldsystems.org/pics/TZFPC/IMG_1831%2B1160775432.JPG	2025-06-11
Bathyraja_violacea	http://www.fishbase.se/images/species/Bavio_u0.jpg	2025-06-11
Bathytoshia_brevicaudata	http://www.fishbase.org/images/species/Dabre_u2.jpg	2025-06-11
Bathytoshia_brevicaudata	http://www.fishbase.org/tools/UploadPhoto/uploads/196lb.jpg	2025-06-11
Bathytoshia_brevicaudata	https://static.inaturalist.org/photos/1203997/medium.?1413271433	2025-06-11
Bathytoshia_brevicaudata	https://static.inaturalist.org/photos/7886577/medium.jpeg?1495170091	2025-06-11
Bathytoshia_centroura	http://shark-references.com/images/species/Dasyatis%20centroura%20S09-D%20BEST.jpg	2025-06-11
Bathytoshia_centroura	http://www.fishbase.org/images/species/Dacen_u0.jpg	2025-06-11
Bathytoshia_centroura	http://www.fishbase.org/images/species/Dacen_u1.jpg	2025-06-11
Bathytoshia_centroura	http://www.fishbase.org/images/species/Dacen_u3.jpg	2025-06-11

Bathytoshia_centroura	https://farm3.staticflickr.com/2666/3712597138_c80ba7ba68.jpg	2025-06-11
Bathytoshia_lata	http://fishesofaustralia.net.au/Images/Image/BathytoshLatadentrock.jpg	2025-06-11
Bathytoshia_lata	http://fishesofaustralia.net.au/Images/Image/BathytoshLataSylkeRohrlach.jpg	2025-06-11
Bathytoshia_lata	http://www.fishbase.org/images/species/Dalat_u0.jpg	2025-06-11
Bathytoshia_lata	http://www.fishbase.org/tools/UploadPhoto/uploads/CIMG3612.JPG	2025-06-11
Bathytoshia_lata	http://www.fishbase.org/tools/UploadPhoto/uploads/CIMG3632.JPG	2025-06-11
Bathytoshia_lata	http://www.fishbase.org/tools/UploadPhoto/uploads/CIMG3648.JPG	2025-06-11
Benthobatis_kreffti	http://www.fishbase.se/images/species/Bekre_u0.jpg	2025-06-11
Benthobatis_kreffti	http://www.fishbase.se/tools/UploadPhoto/uploads/CEPSUL_Be_kr.jpg	2025-06-11
Benthobatis_marcida	http://shark-references.com/images/species/Bethobatis%20marcida%20PE11-8-EPI-A.jpg	2025-06-11
Benthobatis_moresbyi	http://www.fishbase.se/images/species/Bemor_u0.jpg	2025-06-11
Benthobatis_moresbyi	http://www.fishbase.se/photos/workimagethumb.php?s=http://www.fishbase.se/tools/UploadPhoto/uploads/1436774032_117.211.16.93.jpg&w=600	2025-06-11
Benthobatis_moresbyi	http://www.fishbase.se/tools/UploadPhoto/uploads/Benthobatis.jpg	2025-06-11
Benthobatis_yangi	http://shark-references.com/images/species/Taiwanese%20Blind%20Electric%20Ray,%20Benthobatis%20yangi,%20de%20Carvalho,%20Compagno%20&%20Ebert,%202003.jpg	2025-06-11
Beringraja_binoculata	https://inaturalist-open-data.s3.amazonaws.com/photos/176873409/medium.jpeg	2025-06-11
Beringraja_binoculata	https://static.inaturalist.org/photos/140778369/medium.jpeg	2025-06-11
Beringraja_binoculata	https://inaturalist-open-data.s3.amazonaws.com/photos/146714953/large.jpeg	2025-06-11
Beringraja_binoculata	http://shark-references.com/images/species/rajabinocdors.jpg	2025-06-11
Beringraja_binoculata	http://www.fishbase.org/photos/workimagethumb.php?s=http://www.fishbase.org/tools/UploadPhoto/uploads/1395418201_71.202.224.33.jpg&w=600	2025-06-11
Beringraja_binoculata	http://www.fishbase.org/tools/UploadPhoto/uploads/bigskate.jpg	2025-06-11
Beringraja_binoculata	https://www.fishbase.se/images/species/Rabin_u0.jpg	2025-06-11
Beringraja_inornata	https://inaturalist-open-data.s3.amazonaws.com/photos/201422464/medium.jpg	2025-06-11
Beringraja_pulchra	https://shark-references.com/images/species/RajaPulchra_Russia_Survey2014_PANCHENKO,%20BOIKO.jpg	2025-06-11
Beringraja_rhina	http://www.fishbase.org/images/species/Rarhi_u0.jpg	2025-06-11
Beringraja_rhina	http://www.fishbase.org/images/species/Rarhi_u1.jpg	2025-06-11
Beringraja_stellulata	http://www.boldsystems.org/pics/FMV/UW153490%2B1394637582.JPG	2025-06-11
Breviraja_mouldi	https://inaturalist-open-data.s3.amazonaws.com/photos/162139/medium.jpeg	2025-06-11
Breviraja_spinosa	http://www.fishbase.se/images/species/Brspi_m0.jpg	2025-06-11
Breviraja_spinosa	http://www.fishbase.se/images/species/Brspi_u0.jpg	2025-06-11
Brevitrygon_imbricata	http://shark-references.com/images/species/Himatura_imbricata.jpg	2025-06-11
Brevitrygon_imbricata	http://www.fishbase.org/images/species/Hiimb_u0.jpg	2025-06-11
Brevitrygon_imbricata	http://www.fishbase.org/tools/UploadPhoto/uploads/0018ps1imb.jpg	2025-06-11
Brevitrygon_javaensis	http://shark-references.com/images/species/Himantura_javensis.jpg	2025-06-11
Brevitrygon_walga	http://www.fishbase.org/photos/workimagethumb.php?s=http%3A%2F%2Fwww.fishbase.org%2Ftools%2FUploadPhoto%2Fuploads%2F1374653007_182.52.68.233.jpg&w=300	2025-06-11
Brevitrygon_walga	http://www.fishbase.org/tools/UploadPhoto/uploads/1366279893_182.178.77.189.jpg	2025-06-11
Brevitrygon_walga	http://www.fishbase.org/tools/UploadPhoto/uploads/1371882795_182.178.57.199.jpg	2025-06-11
Brevitrygon_walga	http://www.fishbase.org/tools/UploadPhoto/uploads/1371882828_182.178.57.199.jpg	2025-06-11
Brochiraja_heuresa	https://shark-references.com/images/species/Brochiraja_heuresa.jpg	2025-06-11
Brochiraja_vittacauda	http://shark-references.com/images/species/Brochiraja_vittacauda.jpg	2025-06-11
Cruriraja_hulleyi	http://shark-references.com/images/species/Legskate,%20Cruriraja%20hulleyi,%20Aschliman,%20Ebert%20&%20Compagno,%202010.jpg	2025-06-11
Cruriraja_parcomaculata	http://www.boldsystems.org/pics/DSSAU/Cruriraja_parcomaculata_%238%2B1180406536.jpg	2025-06-11

Cruriraja_parcomaculata	http://www.boldsystems.org/pics/ELAME/d001561%2B1260411106.JPG	2025-06-11
Cruriraja_parcomaculata	http://www.boldsystems.org/pics/ELAME/d001570%2B1260411094.JPG	2025-06-11
Cruriraja_parcomaculata	http://www.boldsystems.org/pics/ELAME/d001571%2B1260411094.JPG	2025-06-11
Cruriraja_parcomaculata	http://www.boldsystems.org/pics/ELAME/d001586%2B1260411078.JPG	2025-06-11
Cruriraja_parcomaculata	http://www.boldsystems.org/pics/ELAME/d001645%2B1260411112.JPG	2025-06-11
Cruriraja_parcomaculata	http://www.boldsystems.org/pics/ELAME/d001647%2B1260411092.JPG	2025-06-11
Cruriraja_parcomaculata	http://www.boldsystems.org/pics/ELAME/d001657%2B1260411090.JPG	2025-06-11
Dactylobatus_clarkii	http://www.fishbase.se/images/species/Dacla_u1.jpg	2025-06-11
Dactylobatus_clarkii	http://www.fishbase.se/photos/PicturesSummary.php?ID=7608&what=species	2025-06-11
Dasyatis_chrysonota	http://www.boldsystems.org/pics/HVDBF/DSC00989%2B1354883160.jpg	2025-06-11
Dasyatis_chrysonota	http://www.fishbase.org/images/species/Dachr_u2.jpg	2025-06-11
Dasyatis_hypostigma	http://shark-references.com/images/species/Dasay_u1.jpg	2025-06-11
Dasyatis_hypostigma	http://www.boldsystems.org/pics/EFBD/BPS2500A%2B1227120070.JPG	2025-06-11
Dasyatis_hypostigma	http://www.boldsystems.org/pics/EFBD/BPS2500B%2B1230329712.jpg	2025-06-11
Dasyatis_hypostigma	http://www.boldsystems.org/pics/EFBD/BPS2501A%2B1219440804.jpg	2025-06-11
Dasyatis_hypostigma	http://www.boldsystems.org/pics/FARGB/UNMDP_DI_0564%2B1305862234.jpg	2025-06-11
Dasyatis_hypostigma	http://www.boldsystems.org/pics/FARGB/UNMDP_DI_0565%2B1295652242.jpg	2025-06-11
Dasyatis_hypostigma	http://www.boldsystems.org/pics/FARGB/UNMDP_DI_0695%2B1300415058.jpg	2025-06-11
Dasyatis_hypostigma	http://www.boldsystems.org/pics/FARGB/UNMDP-DI_0460_dorsal%2B1312465212.JPG	2025-06-11
Dasyatis_hypostigma	http://www.fishbase.org/images/species/Dahyp_u0.jpg	2025-06-11
Dasyatis_marmorata	http://www.boldsystems.org/pics/EFBD/BPS0533A%2B1009861200.JPG	2025-06-11
Dasyatis_marmorata	http://www.fishbase.org/images/species/Dachr_u0.jpg	2025-06-11
Dasyatis_marmorata	http://www.fishbase.org/images/species/Dachr_u1.jpg	2025-06-11
Dasyatis_pastinaca	http://www.boldsystems.org/pics/BIM/D06.1_Dasyatis_pastinaca%2B1372110792.JPG	2025-06-11
Dasyatis_pastinaca	http://www.boldsystems.org/pics/BIM/D12.2%2B978318000.JPG	2025-06-11
Dasyatis_pastinaca	http://www.boldsystems.org/pics/BIM/E81-6%2B1383857286.JPG	2025-06-11
Dasyatis_pastinaca	http://www.boldsystems.org/pics/EFBD/BPS1216A%2B1239995568.JPG	2025-06-11
Dasyatis_pastinaca	http://www.boldsystems.org/pics/EFBD/BPS1399A%2B1273081574.JPG	2025-06-11
Dasyatis_pastinaca	http://www.boldsystems.org/pics/ELAME/10PP09DPA_dors%2B1318363190.JPG	2025-06-11
Dasyatis_pastinaca	http://www.boldsystems.org/pics/ELAME/ALG2010_076%2B1318363074.jpg	2025-06-11
Dasyatis_pastinaca	http://www.fishbase.org/images/species/Dapas_u2.jpg	2025-06-11
Dasyatis_pastinaca	http://www.fishbase.org/images/species/Dapas_u4.jpg	2025-06-11
Dasyatis_pastinaca	http://www.fishbase.org/tools/UploadPhoto/uploads/Pastinaca_dasyatis_pastinaca_2262.jpg	2025-06-11
Dasyatis_tortonesei	http://www.boldsystems.org/pics/EFBD/BPS0548A%2B1009861200.JPG	2025-06-11
Dasyatis_tortonesei	http://www.boldsystems.org/pics/EFBD/BPS2498A%2B1191023970.JPG	2025-06-11
Dasyatis_tortonesei	http://www.boldsystems.org/pics/ELAME/CNRAMCMazara116%2B1214514926.JPG	2025-06-11
Dasyatis_tortonesei	http://www.boldsystems.org/pics/ELAME/CNRAMCMazara118%2B1214515360.JPG	2025-06-11
Dasyatis_tortonesei	http://www.fishbase.org/images/species/Dator_u1.jpg	2025-06-11
Dentiraja_australis	http://www.fishbase.se/images/species/Okaus_f0.jpg	2025-06-11
Dentiraja_cerva	http://shark-references.com/images/species/Dipturus_cerva_main.jpg	2025-06-11
Dentiraja_confusa	http://shark-references.com/images/species/Dipturus_confusus_first.jpg	2025-06-11
Dentiraja_confusa	http://shark-references.com/images/species/Dipturus_confusus_main.jpg	2025-06-11
Dentiraja_endeavouri	http://shark-references.com/images/species/Dipturus_endeavouri_main.jpg	2025-06-11

Dentiraja_endeavouri	http://shark-references.com/images/species/Dipturus_endeavouri_paratype.jpg	2025-06-11
Dentiraja_falloarga	http://shark-references.com/images/species/Dipturus_falloargus_first.jpg	2025-06-11
Dentiraja_falloarga	http://shark-references.com/images/species/Dipturus_falloargus_main.jpg	2025-06-11
Dentiraja_healdi	http://shark-references.com/images/species/Dipturus_healdi_first.jpg	2025-06-11
Dentiraja_healdi	http://shark-references.com/images/species/Dipturus_healdi_main.jpg	2025-06-11
Dentiraja_lemprieri	http://fishesofaustralia.net.au/images/image/DentirajaLemprieriRLS.jpg	2025-06-11
Dentiraja_lemprieri	http://fishesofaustralia.net.au/Images/Image/DentirajaLemprieriWW.jpg	2025-06-11
Dentiraja_lemprieri	http://fishesofaustralia.net.au/Images/Image/DipturusLemprieriRK.jpg	2025-06-11
Dentiraja_lemprieri	http://shark-references.com/images/species/Dipturus_lemprieri_main.jpg	2025-06-11
Dentiraja_oculata	http://shark-references.com/images/species/Dipturus_oculus_first.jpg	2025-06-11
Dentiraja_oculata	http://shark-references.com/images/species/Dipturus_oculus_main.jpg	2025-06-11
Dentiraja_polyommata	http://shark-references.com/images/species/Dipturus_polyommata_main.jpg	2025-06-11
Dentiraja_polyommata	http://www.fishbase.se/images/species/Rapol_m0.jpg	2025-06-11
Diplobatis_ommata	http://www.fishbase.se/photos/PicturesSummary.php?ID=8723&what=specieshttp://elasmodiver.com/elasmodiver_home.htm	2025-06-11
Diplobatis_ommata	http://www.fishbase.se/photos/PicturesSummary.php?StartRow=0&ID=8723&what=species&TotRec=5	2025-06-11
Diplobatis_picta	http://www.fishbase.se/images/species/Dipic_f0.jpg	2025-06-11
Dipturus_acrobelus	http://fishesofaustralia.net.au/images/image/DipturusAcrobelasCSIRO.jpg	2025-06-11
Dipturus_acrobelus	http://shark-references.com/images/species/Dipturus_acrobelus_first.jpg	2025-06-11
Dipturus_acrobelus	http://shark-references.com/images/species/Dipturus_acrobelus_main.jpg	2025-06-11
Dipturus_acrobelus	http://shark-references.com/images/species/Dipturus_acrobelus_paratype.jpg	2025-06-11
Dipturus_apricus	http://shark-references.com/images/species/Dipturus_apricus_first.jpg	2025-06-11
Dipturus_apricus	http://shark-references.com/images/species/Dipturus_apricus_main.jpg	2025-06-11
Dipturus_batis	http://www.boldsystems.org/pics/EFBB/BPS2266A%2B1346441272.JPG	2025-06-11
Dipturus_batis	http://www.fishbase.se/images/species/Dibat_m0.jpg	2025-06-11
Dipturus_batis	http://www.fishbase.se/images/species/Dibat_u0.jpg	2025-06-11
Dipturus_batis	http://www.fishbase.se/tools/UploadPhoto/uploads/130lbcommon.jpg	2025-06-11
Dipturus_batis	http://www.fishbase.se/tools/UploadPhoto/uploads/1419176895_82.156.224.224.jpg	2025-06-11
Dipturus_batis	http://www.fishbase.se/tools/UploadPhoto/uploads/D.batis2x.jpg	2025-06-11
Dipturus_bullisi	http://www.fishbase.se/images/species/Dibul_m0.jpg	2025-06-11
Dipturus_canutus	http://shark-references.com/images/species/Dipturus_canutus_first.jpg	2025-06-11
Dipturus_canutus	http://shark-references.com/images/species/Dipturus_canutus_main.jpg	2025-06-11
Dipturus_grahami	http://fishesofaustralia.net.au/images/image/DipturusGrahamiCSIRO.jpg	2025-06-11
Dipturus_grahami	http://shark-references.com/images/species/Dipturus_grahami_main.jpg	2025-06-11
Dipturus_gudgeri	http://fishesofaustralia.net.au/images/image/DipturusGudgeriCSIRO.jpg	2025-06-11
Dipturus_gudgeri	http://shark-references.com/images/species/Dipturus_gudgeri_main.jpg	2025-06-11
Dipturus_johannisdavisi	http://www.fishbase.se/images/species/Dijoh_u0.jpg	2025-06-11
Dipturus_johannisdavisi	http://www.fishbase.se/tools/UploadPhoto/uploads/Djo.jpg	2025-06-11
Dipturus_kwangtungensis	http://shark-references.com/images/species/Dipturus-kwangtungensis_main.jpg	2025-06-11
Dipturus_kwangtungensis	http://www.fishbase.se/images/species/Dikwa_u0.jpg	2025-06-11
Dipturus_laevis	http://cdn1.arkive.org/media/3B/3B4ED78D-CA28-4366-B188-9ECFDF4BE324/Presentation.Large/Barndoor-skate-.jpg	2025-06-11
Dipturus_laevis	http://cdn2.arkive.org/media/FE/FECC1B39-D755-47B5-A090-6F5BB6EE3926/Presentation.Large/Pair-of-barndoor-skate-.jpg	2025-06-11
Dipturus_laevis	http://www.boldsystems.org/pics/ELAME/d000680%2B1260411086.JPG	2025-06-11
Dipturus_laevis	http://www.boldsystems.org/pics/SCAFB/07-249%2B1185898148.JPG	2025-06-11

Dipturus_laevis	http://www.boldsystems.org/pics/SCFAC/06-125%2B1217536958.jpg	2025-06-11
Dipturus_laevis	http://www.fishbase.se/images/species/Dilae_u0.jpg	2025-06-11
Dipturus_macrocaudus	http://www.fishbase.se/images/species/Dimac_u3.jpg	2025-06-11
Dipturus_melanospilus	http://fishesofaustralia.net.au/images/image/DipturusMelanospilusCSIRO.jpg	2025-06-11
Dipturus_melanospilus	http://shark-references.com/images/species/Dipturus_melanospilus_first.jpg	2025-06-11
Dipturus_melanospilus	http://shark-references.com/images/species/Dipturus_melanospilus_main.jpg	2025-06-11
Dipturus_nidarosiensis	http://www.boldsystems.org/pics/EFBB/BPS0061A%2B1336799068.jpg	2025-06-11
Dipturus_nidarosiensis	http://www.boldsystems.org/pics/EFBB/BPS0226A%2B1009854004.JPG	2025-06-11
Dipturus_nidarosiensis	http://www.boldsystems.org/pics/EFBB/BPS0547A%2B1009857600.JPG	2025-06-11
Dipturus_nidarosiensis	http://www.boldsystems.org/pics/EFBB/BPS0587C%2B1158606420.JPG	2025-06-11
Dipturus_nidarosiensis	http://www.boldsystems.org/pics/EFBB/BPS0958A%2B1009857600.JPG	2025-06-11
Dipturus_olseni	http://www.fishbase.se/images/species/Diols_u0.jpg	2025-06-11
Dipturus_oregoni	https://fishbase.se/tools/display_image.php?fw=n&imgName=1517468894_172.68.106.30.jpg	2025-06-11
Dipturus_oxyrinchus	http://www.boldsystems.org/pics/ELAME/1Gr08D6dorsROX%2B1260411068.JPG	2025-06-11
Dipturus_oxyrinchus	http://www.boldsystems.org/pics/ELAME/2Gr08D6dorsROX%2B1260411088.JPG	2025-06-11
Dipturus_oxyrinchus	http://www.boldsystems.org/pics/ELAME/3Gr08D6dorsROX%2B1260411102.JPG	2025-06-11
Dipturus_oxyrinchus	http://www.boldsystems.org/pics/ELAME/4Gr08D6dorsROX%2B1260411082.JPG	2025-06-11
Dipturus_oxyrinchus	http://www.boldsystems.org/pics/RNEZ/RNEZ165D%2B1265310524.jpg	2025-06-11
Dipturus_oxyrinchus	http://www.boldsystems.org/pics/RNEZ/RNEZ172D%2B1265311384.jpg	2025-06-11
Dipturus_oxyrinchus	http://www.boldsystems.org/pics/RNEZ/RNEZ220D%2B1265319650.jpg	2025-06-11
Dipturus_oxyrinchus	http://www.boldsystems.org/pics/RNEZ/RNEZ222D%2B1265321736.jpg	2025-06-11
Dipturus_pullopunctatus	http://www.boldsystems.org/pics/ELAME/d001611%2B1260411108.JPG	2025-06-11
Dipturus_pullopunctatus	http://www.boldsystems.org/pics/ELAME/d001667%2B1260411102.JPG	2025-06-11
Dipturus_pullopunctatus	http://www.boldsystems.org/pics/HVDB/IMG_4775%2B1316181288.JPG	2025-06-11
Dipturus_pullopunctatus	http://www.fishbase.se/images/species/Dipul_u0.jpg	2025-06-11
Dipturus_queenslandicus	http://fishesofaustralia.net.au/images/image/DipturusQueenslandicusCSIRO.jpg	2025-06-11
Dipturus_teevani	http://www.boldsystems.org/pics/CBPM/COB049%2B1306428740.JPG	2025-06-11
Dipturus_teevani	http://www.fishbase.se/images/species/Ditee_f0.jpg	2025-06-11
Dipturus_tengu	http://www.fishbase.se/images/species/Diten_u0.jpg	2025-06-11
Dipturus_trachydermus	http://shark-references.com/images/species/Dipturus_trachyderma_mtDNA.jpg	2025-06-11
Dipturus_wengi	http://shark-references.com/images/species/Dipturus_wengi_first.jpg	2025-06-11
Dipturus_wengi	http://shark-references.com/images/species/Dipturus_wengi_main.jpg	2025-06-11
Discopyge_tschudii	http://www.boldsystems.org/pics/FARG/INIDEP-DI_0798%2B1254767706.jpg	2025-06-11
Discopyge_tschudii	http://www.boldsystems.org/pics/FARGB/UNMDP_DI_096%2B1303442386.jpg	2025-06-11
Discopyge_tschudii	http://www.boldsystems.org/pics/FARGB/UNMDP-DI_064%2B1312408658.JPG	2025-06-11
Discopyge_tschudii	http://www.fishbase.se/images/species/Ditsc_u0.jpg	2025-06-11
Discopyge_tschudii	http://www.fishbase.se/images/species/Ditsc_u1.jpg	2025-06-11
Discopyge_tschudii	http://www.fishbase.se/images/species/Ditsc_u2.jpg	2025-06-11
Discopyge_tschudii	http://www.fishbase.se/tools/UploadPhoto/uploads/P7220036.JPG	2025-06-11
Electrolux_addisoni	http://www.boldsystems.org/pics/SAIAB/SAIAB_78450-T633%2B1159982704.jpg	2025-06-11
Fenestraja_ishiyamai	http://shark-references.com/images/species/Fenestraja-ishiyamai.jpg	2025-06-11
Fenestraja_plutonia	http://www.fishbase.se/images/species/Feplu_u0.jpg	2025-06-11
Fluvitrygon_kittipongi	http://www.fishbase.org/images/species/Hikit_u0.jpg	2025-06-11

Fluvitrygon_signifer	https://shark-references.com/images/species/Fluvitrygon_signifer_Sumatra.jpg	2025-06-11
Fluvitrygon_signifer	https://shark-references.com/images/species/Fluvitrygon_signifer_Sumatra.jpg	2025-06-11
Fluvitrygon_signifer	https://www.inaturalist.org/photos/172031565	2025-06-11
Fontitrygon_geijskesi	http://www.fishbase.org/images/species/Dagei_u0.jpg	2025-06-11
Fontitrygon_margarita	http://www.fishbase.org/tools/UploadPhoto/uploads/Dasyatis_margarita.JPG	2025-06-11
Fontitrygon_margaritella	http://www.fishbase.org/images/species/Damar_u3.jpg	2025-06-11
Glaucostegus_cemiculus	http://www.fishbase.se/images/species/Rhcem_u0.jpg	2025-06-12
Glaucostegus_cemiculus	http://www.fishbase.se/photos/workimagethumb.php?s=http://www.fishbase.se/tools/UploadPhoto/uploads/1396947169_78.221.206.67.jpg&w=600	2025-06-12
Glaucostegus_cemiculus	http://www.fishbase.se/tools/UploadPhoto/uploads/DSCN2789.JPG	2025-06-12
Glaucostegus_cemiculus	http://www.fishbase.se/tools/UploadPhoto/uploads/Pesce_violino_Rhinobatos_rhinobatos_Genova_7191_95.jpg	2025-06-12
Glaucostegus_granulatus	http://shark-references.com/images/species/8731_Rhgra_u1.jpg	2025-06-12
Glaucostegus_granulatus	http://www.fishbase.se/photos/workimagethumb.php?s=http://www.fishbase.se/tools/UploadPhoto/uploads/1383728409_182.52.68.117.jpg&w=600	2025-06-12
Glaucostegus_granulatus	http://www.fishbase.se/photos/workimagethumb.php?s=http://www.fishbase.se/tools/UploadPhoto/uploads/1383728639_182.52.68.117.jpg&w=600	2025-06-12
Glaucostegus_granulatus	http://www.fishbase.se/photos/workimagethumb.php?s=http://www.fishbase.se/tools/UploadPhoto/uploads/1401027998_182.178.24.155.jpg&w=600	2025-06-12
Glaucostegus_granulatus	http://www.fishbase.se/tools/UploadPhoto/uploads/1371897309_182.178.38.182.jpg	2025-06-12
Glaucostegus_granulatus	http://www.fishbase.se/tools/UploadPhoto/uploads/1371897381_182.178.38.182.jpg	2025-06-12
Glaucostegus_halavi	http://www.fishbase.se/images/species/Rhhal_u0.jpg	2025-06-12
Glaucostegus_halavi	http://www.fishbase.se/images/species/Rhhal_u1.jpg	2025-06-12
Glaucostegus_halavi	http://www.fishbase.se/photos/workimagethumb.php?s=http://www.fishbase.se/tools/UploadPhoto/uploads/1435946816_39.57.112.249.jpg&w=600	2025-06-12
Glaucostegus_halavi	http://www.fishbase.se/tools/UploadPhoto/uploads/1364061794_182.178.14.100.jpg	2025-06-12
Glaucostegus_obtusus	http://www.boldsystems.org/pics/ANGEN/sample_104%2B1433347648.jpg	2025-06-12
Glaucostegus_obtusus	http://www.fishbase.se/images/species/Rhobt_u1.jpg	2025-06-12
Glaucostegus_obtusus	http://www.fishbase.se/photos/workimagethumb.php?s=http://www.fishbase.se/tools/UploadPhoto/uploads/1374049388_182.52.68.240.jpg&w=600	2025-06-12
Glaucostegus_obtusus	http://www.fishbase.se/photos/workimagethumb.php?s=http://www.fishbase.se/tools/UploadPhoto/uploads/1383230211_182.178.98.78.jpg&w=600	2025-06-12
Glaucostegus_obtusus	http://www.fishbase.se/photos/workimagethumb.php?s=http://www.fishbase.se/tools/UploadPhoto/uploads/1384449244_182.178.70.122.jpg&w=600	2025-06-12
Glaucostegus_obtusus	http://www.fishbase.se/photos/workimagethumb.php?s=http://www.fishbase.se/tools/UploadPhoto/uploads/1400405452_182.178.61.167.jpg&w=600	2025-06-12
Glaucostegus_thouin	http://www.fishbase.se/images/species/Rhtho_u4.jpg	2025-06-12
Glaucostegus_thouin	http://www.fishbase.se/tools/UploadPhoto/uploads/1365909319_182.178.95.68.jpg	2025-06-12
Glaucostegus_thouin	http://www.fishbase.se/tools/UploadPhoto/uploads/1367079603_182.178.80.166.jpg	2025-06-12
Glaucostegus_typus	http://fishesofaustralia.net.au/Images/Image/GlaucostegTypusAnneHoggett.jpg	2025-06-12
Glaucostegus_typus	http://www.boldsystems.org/pics/IFV/KMF113%2B1490378290.JPG	2025-06-12
Glaucostegus_typus	http://www.fishbase.se/images/species/Gltyp_u0.jpg	2025-06-12
Glaucostegus_typus	http://www.fishbase.se/tools/UploadPhoto/uploads/dsc00564x.jpg	2025-06-12
Gurgesiella_dorsalifera	http://www.fishbase.se/images/species/Gudor_u0.jpg	2025-06-12
Gurgesiella_dorsalifera	http://www.fishbase.se/images/species/Gudor_u1.jpg	2025-06-12
Gurgesiella_dorsalifera	http://www.fishbase.se/tools/UploadPhoto/uploads/Gurgesielladorsalifera.JPG	2025-06-12
Gurgesiella_furvescens	http://www.fishbase.se/images/species/Gufur_f0.jpg	2025-06-12
Gurgesiella_furvescens	http://www.fishbase.se/images/species/Gufur_f1.jpg	2025-06-12
Gymnura_altavela	http://shark-references.com/images/species/Gymnura_altavela_5.jpg	2025-06-12
Gymnura_altavela	http://www.boldsystems.org/pics/BIM/D13.2%2B1382644942.JPG	2025-06-12
Gymnura_altavela	http://www.fishbase.se/images/species/Gyalt_u0.jpg	2025-06-12
Gymnura_altavela	http://www.fishbase.se/images/species/Gyalt_u1.jpg	2025-06-12
Gymnura_australis	http://www.fishbase.se/images/species/Gyaus_u0.jpg	2025-06-12

Gymnura_crebripunctata	http://www.fishbase.se/images/species/Gycre_u0.jpg	2025-06-12
Gymnura_japonica	http://www.fishbase.se/images/species/Gyjap_u1.jpg	2025-06-12
Gymnura_japonica	http://www.fishbase.se/photos/workimagethumb.php?s=http://www.fishbase.se/tools/UploadPhoto/uploads/1374652321_182.52.68.233.jpg&w=600	2025-06-12
Gymnura_marmorata	http://shark-references.com/images/species/Gymnura_marmorata.jpg	2025-06-12
Gymnura_marmorata	http://www.discoverlife.org/IM/I_RR/0035/320/Gymnura_marmorata,I_RR3508.jpg	2025-06-12
Gymnura_marmorata	http://www.discoverlife.org/IM/I_RR/0036/320/Gymnura_marmorata,I_RR3604.jpg	2025-06-12
Gymnura_marmorata	http://www.fishbase.org/images/species/Gymar_j0.jpg	2025-06-12
Gymnura_marmorata	http://www.fishbase.org/images/species/Gymar_u1.jpg	2025-06-12
Gymnura_micrura	http://shark-references.com/images/species/Gymnura_micrura.jpg	2025-06-12
Gymnura_micrura	http://shark-references.com/images/species/Gymnura%20%20micrura%20%20S05-B%20BEST.jpg	2025-06-12
Gymnura_micrura	http://www.boldsystems.org/pics/FLBAR/FWRI_01496_0048edits%2B1377633198.jpg	2025-06-12
Gymnura_micrura	http://www.boldsystems.org/pics/MEFM/MEFM687-06%2B1149696138.JPG	2025-06-12
Gymnura_micrura	http://www.boldsystems.org/pics/MXIV/MXIV568%2B1277254184.JPG	2025-06-12
Gymnura_micrura	http://www.boldsystems.org/pics/MXIV/MXIV590%2B1277256372.JPG	2025-06-12
Gymnura_micrura	http://www.fishbase.org/tools/UploadPhoto/uploads/Gmicrura.jpg	2025-06-12
Gymnura_micrura	http://www.fishbase.org/tools/UploadPhoto/uploads/IMG00037-20110626-1150.jpg	2025-06-12
Gymnura_micrura	https://static.inaturalist.org/photos/4502759/medium.jpg?1470772017	2025-06-12
Gymnura_micrura	https://upload.wikimedia.org/wikipedia/commons/thumb/f/fd/Gymnura_micrura.jpg/500px-Gymnura_micrura.jpg	2025-06-12
Gymnura_natalensis	http://www.boldsystems.org/pics/HVDBF/DSC00995%2B1357824092.jpg	2025-06-12
Gymnura_natalensis	http://www.boldsystems.org/pics/HVDBF/DSC00996%2B1354883282.jpg	2025-06-12
Gymnura_natalensis	http://www.fishbase.org/tools/UploadPhoto/uploads/1351799978_41.151.218.155.jpg	2025-06-12
Gymnura_poecilura	http://shark-references.com/images/species/Gpoecilura.jpg	2025-06-12
Gymnura_poecilura	http://shark-references.com/images/species/Gymnura_poecilura_India_juvenile.jpg	2025-06-12
Gymnura_poecilura	http://www.boldsystems.org/pics/INELA/Gymnura_poecilura_02%2B1352930694.jpg	2025-06-12
Gymnura_poecilura	http://www.boldsystems.org/pics/INELA/Gymnura_poecilura_04%2B1312837454.jpg	2025-06-12
Gymnura_poecilura	http://www.boldsystems.org/pics/JTFR/PDGN01%2B1497329108.jpg	2025-06-12
Gymnura_poecilura	http://www.boldsystems.org/pics/JTFR/PDGN02%2B1497328430.jpg	2025-06-12
Gymnura_poecilura	http://www.fishbase.org/images/species/Gypoe_u0.jpg	2025-06-12
Gymnura_poecilura	http://www.fishbase.org/images/species/Gypoe_u2.jpg	2025-06-12
Gymnura_poecilura	http://www.fishbase.org/tools/UploadPhoto/uploads/1366994285_182.178.16.188.jpg	2025-06-12
Gymnura_poecilura	http://www.fishbase.org/tools/UploadPhoto/uploads/1382791135_194.225.167.210.jpg	2025-06-12
Gymnura_poecilura	http://www.fishbase.org/tools/UploadPhoto/uploads/1486543035_172.68.106.90.jpg	2025-06-12
Gymnura_poecilura	http://www.fishbase.org/tools/UploadPhoto/uploads/img_3599.jpg	2025-06-12
Gymnura_zonura	http://www.fishbase.org/images/species/Aezon_u0.jpg	2025-06-12
Gymnura_zonura	http://www.fishbase.org/tools/UploadPhoto/uploads/1483002324_162.158.165.102.jpg	2025-06-12
Hemitrygon_akajei	http://www.fishbase.org/tools/UploadPhoto/uploads/1371713498_182.52.68.125.jpg	2025-06-12
Hemitrygon_bennetti	http://shark-references.com/images/species/Dasyatis%20bennettii.jpg	2025-06-12
Hemitrygon_bennetti	http://www.boldsystems.org/pics/FSCS/P1000315%2B1194119414.JPG	2025-06-12
Hemitrygon_bennetti	http://www.boldsystems.org/pics/FSCS/P1000317%2B1194119526.JPG	2025-06-12
Hemitrygon_bennetti	http://www.boldsystems.org/pics/FSCS/P1000318%2B1194119582.JPG	2025-06-12
Hemitrygon_bennetti	http://www.boldsystems.org/pics/FSCS/P1000321%2B1194119680.JPG	2025-06-12
Hemitrygon_bennetti	http://www.fishbase.org/images/species/Daben_u0.jpg	2025-06-12
Hemitrygon_bennetti	http://www.fishbase.org/tools/UploadPhoto/uploads/Dasyatisbennettii.jpg	2025-06-12

Hemitrygon_fluviorum	http://fishesofaustralia.net.au/images/image/HemitryFluviorHarryRosenthal.jpg	2025-06-12
Hemitrygon_izuensis	http://shark-references.com/images/species/Dasyatis_izuensis.jpg	2025-06-12
Hemitrygon_izuensis	http://www.fishbase.org/images/species/Daizu_u0.jpg	2025-06-12
Hemitrygon_laosensis	http://www.fishbase.se/images/species/Dalao_u0.jpg	2025-06-12
Hemitrygon_laosensis	http://www.fishbase.se/images/species/Dalao_u1.jpg	2025-06-12
Hemitrygon_longicauda	http://shark-references.com/images/species/Dasyatis_longicauda.jpg	2025-06-12
Hemitrygon_navarrae	http://www.fishbase.se/images/species/Danav_u0.jpg	2025-06-12
Hemitrygon_parvonigra	http://fishesofaustralia.net.au/images/image/DasyatisParvonigraCSIRO.jpg	2025-06-12
Hemitrygon_parvonigra	http://www.fishbase.se/images/species/Dapar_u0.jpg	2025-06-12
Heteronarce_bentuviai	http://www.fishbase.org/images/species/Heben_u0.jpg	2025-06-12
Heteronarce_bentuviai	http://www.fishbase.org/tools/UploadPhoto/uploads/Heteronarce-bentuviai.jpg	2025-06-12
Heteronarce_bentuviai	https://www.researchgate.net/publication/261671366_Narcine_bentuviai_a_new_torpedinoid_ray_from_the_northern_red_sea	2025-06-12
Hexatrygon_bickelli	http://fishesofaustralia.net.au/images/image/HexatrygonBickelliCSIRO.jpg	2025-06-12
Hexatrygon_bickelli	http://www.boldsystems.org/pics/ANCC/BPS0314A%2B1024300074.jpg	2025-06-12
Hexatrygon_bickelli	http://www.fishbase.se/photos/workimagethumb.php?s=http://www.fishbase.se/tools/UploadPhoto/uploads/1405427535_117.239.146.240.jpeg&w=600	2025-06-12
Himantura_australis	http://fishesofaustralia.net.au/Images/Image/HimantAustralBenHeinrichs.jpg	2025-06-12
Himantura_australis	http://fishesofaustralia.net.au/Images/Image/HimantAustralisKennethFoster.jpg	2025-06-12
Himantura_australis	http://fishesofaustralia.net.au/images/image/HimantUarnak.jpg	2025-06-12
Himantura_australis	http://fishesofaustralia.net.au/Images/Image/HimanturaUarnak2AndyALewis.jpg	2025-06-12
Himantura_australis	http://fishesofaustralia.net.au/Images/Image/HimanturAustralRobertNyman.jpg	2025-06-12
Himantura_australis	http://shark-references.com/images/species/Himantura_australis_main.jpg	2025-06-12
Himantura_leoparda	http://fishesofaustralia.net.au/Images/Image/HimanturaLeoparda2CSIRO.jpg	2025-06-12
Himantura_leoparda	http://shark-references.com/images/species/Himantura_leoparda_main.jpg	2025-06-12
Himantura_leoparda	http://www.boldsystems.org/pics/FEE/IMSMETU-HL-01-1%2B1465253752.jpg	2025-06-12
Himantura_leoparda	http://www.boldsystems.org/pics/JTFR/PDGN04%2B1497328876.jpg	2025-06-12
Himantura_leoparda	http://www.boldsystems.org/pics/JTFR/PDGN05%2B1497329258.jpg	2025-06-12
Himantura_leoparda	http://www.fishbase.se/images/species/Hileo_u0.jpg	2025-06-12
Himantura_leoparda	http://www.fishbase.se/images/species/Hiuar_u4.jpg	2025-06-12
Himantura_leoparda	http://www.fishbase.se/photos/workimagethumb.php?s=http://www.fishbase.se/tools/UploadPhoto/uploads/1428142102_39.50.180.66.jpg&w=600	2025-06-12
Himantura_leoparda	http://www.fishbase.se/photos/workimagethumb.php?s=http://www.fishbase.se/tools/UploadPhoto/uploads/1428158036_39.44.76.37.jpg&w=600	2025-06-12
Himantura_leoparda	http://www.fishbase.se/tools/UploadPhoto/uploads/1360248801_117.207.255.41.jpg	2025-06-12
Himantura_leoparda	http://www.fishbase.se/tools/UploadPhoto/uploads/1366126677_182.178.81.169.jpg	2025-06-12
Himantura_uarnak	http://shark-references.com/images/species/5507_Hiuar_u4.jpg	2025-06-12
Himantura_uarnak	http://shark-references.com/images/species/Himantura_tutul_Zanzibar.jpg	2025-06-12
Himantura_uarnak	http://www.boldsystems.org/pics/MAHAN/DSC_093904%2B1458068910.jpg	2025-06-12
Himantura_uarnak	http://www.boldsystems.org/pics/SERIP/Himantura_uarnak_WJC637b%2B1465747362.jpg	2025-06-12
Himantura_uarnak	http://www.fishbase.se/images/species/Hiuar_u1.jpg	2025-06-12
Himantura_uarnak	http://www.fishbase.se/images/species/Hiuar_u2.jpg	2025-06-12
Himantura_uarnak	http://www.fishbase.se/images/species/Hiuar_u3.jpg	2025-06-12
Himantura_uarnak	http://www.fishbase.se/photos/workimagethumb.php?s=http://www.fishbase.se/tools/UploadPhoto/uploads/1375392874_83.89.41.145.jpg&w=600	2025-06-12
Himantura_uarnak	http://www.fishbase.se/photos/workimagethumb.php?s=http://www.fishbase.se/tools/UploadPhoto/uploads/1396655066_71.202.224.33.jpg&w=600	2025-06-12
Himantura_uarnak	http://www.fishbase.se/photos/workimagethumb.php?s=http://www.fishbase.se/tools/UploadPhoto/uploads/1436892561_222.124.71.149.jpg&w=600	2025-06-12

Himantura_uarnak	http://www.fishbase.se/photos/workimagethumb.php?s=http://www.fishbase.se/tools/UploadPhoto/uploads/1440753798_101.109.73.69.jpg&w=600	2025-06-12
Himantura_uarnak	http://www.fishbase.se/tools/UploadPhoto/uploads/1428514568_39.44.210.202.jpg	2025-06-12
Himantura_undulata	http://www.fishbase.se/images/species/Hiund_u0.jpg	2025-06-12
Himantura_undulata	http://www.fishbase.se/photos/workimagethumb.php?s=http://www.fishbase.se/tools/UploadPhoto/uploads/1440972348_2.238.54.39.jpg&w=600	2025-06-12
Himantura_undulata	http://www.fishbase.se/photos/workimagethumb.php?s=http://www.fishbase.se/tools/UploadPhoto/uploads/1441853914_101.109.72.177.jpg&w=600	2025-06-12
Himantura_undulata	http://www.fishbase.se/photos/workimagethumb.php?s=http://www.fishbase.se/tools/UploadPhoto/uploads/1441854336_101.109.72.177.jpg&w=600	2025-06-12
Himantura_undulata	http://www.fishbase.se/tools/UploadPhoto/uploads/13032009030.jpg	2025-06-12
Himantura_undulata	http://www.fishbase.se/tools/UploadPhoto/uploads/1365140187_124.124.120.94.jpg	2025-06-12
Himantura_undulata	http://www.fishbase.se/tools/UploadPhoto/uploads/140920096871.jpg	2025-06-12
Himantura_undulata	http://www.fishbase.se/tools/UploadPhoto/uploads/1440971603_2.238.54.39.jpg	2025-06-12
Hypanus_americanus	http://shark-references.com/images/species/1247_Daame_u0.jpg	2025-06-12
Hypanus_americanus	http://shark-references.com/images/species/Dasyatis-americana.jpg	2025-06-12
Hypanus_americanus	http://shark-references.com/images/species/Dasyatis%20americana_N04-EPI-C_%20BEST.jpg	2025-06-12
Hypanus_dipterurus	http://www.fishbase.se/images/species/Dadip_u0.jpg	2025-06-12
Hypanus_guttatus	http://shark-references.com/images/species/D_guttata_Costa_3.jpg	2025-06-12
Hypanus_guttatus	http://shark-references.com/images/species/D_guttata_Costa_4.jpg	2025-06-12
Hypanus_guttatus	http://shark-references.com/images/species/Dagut_u2.jpg	2025-06-12
Hypanus_guttatus	http://www.fishbase.se/images/species/Dagut_f0.jpg	2025-06-12
Hypanus_guttatus	http://www.fishbase.se/images/species/Dagut_m0.jpg	2025-06-12
Hypanus_guttatus	http://www.fishbase.se/images/species/Dagut_u0.jpg	2025-06-12
Hypanus_guttatus	http://www.fishbase.se/images/species/Dagut_u1.jpg	2025-06-12
Hypanus_guttatus	http://www.fishbase.se/images/species/Dagut_u3.jpg	2025-06-12
Hypanus_guttatus	http://www.fishbase.se/tools/UploadPhoto/uploads/Dasyatisguttata.jpg	2025-06-12
Hypanus_guttatus	http://www.fishbase.se/tools/UploadPhoto/uploads/DasyatisguttataRaphaelMacieira01.jpg	2025-06-12
Hypanus_guttatus	http://www.fishbase.se/tools/UploadPhoto/uploads/Dguttata.jpg	2025-06-12
Hypanus_longus	https://fishbase.se/tools/display_image.php?fw=n&imgName=1633235714_172.70.82.48.jpg	2025-06-12
Hypanus_longus	http://www.fishbase.se/images/species/Dalon_u0.jpg	2025-06-12
Hypanus_longus	http://www.fishbase.se/images/species/Dalon_u1.jpg	2025-06-12
Hypanus_longus	https://static.inaturalist.org/photos/2368017/medium.jpg?1441948729	2025-06-12
Hypanus_marianae	http://shark-references.com/images/species/Dasyatis_marianae_Costa_3.jpg	2025-06-12
Hypanus_marianae	http://shark-references.com/images/species/Dasyatis_marianae_Costa_4.jpg	2025-06-12
Hypanus_marianae	http://www.fishbase.se/images/species/Damar_u6.jpg	2025-06-12
Hypanus_marianae	http://www.fishbase.se/tools/UploadPhoto/uploads/DASMAR.jpg	2025-06-12
Hypanus_sabinus	http://shark-references.com/images/species/Dasyatis-sabina.jpg	2025-06-12
Hypanus_sabinus	http://www.boldsystems.org/pics/MXV/MXV0621%2B1400519184.jpg	2025-06-12
Hypanus_sabinus	http://www.fishbase.se/images/species/Dasab_u0.jpg	2025-06-12
Hypanus_sabinus	http://www.fishbase.se/images/species/Dasab_u1.jpg	2025-06-12
Hypanus_say	http://shark-references.com/images/species/Dasyatis%20say%20S12-A%20BEST.jpg	2025-06-12
Hypanus_say	http://www.fishbase.se/images/species/Dasay_u0.jpg	2025-06-12
Hypanus_say	http://www.fishbase.se/images/species/Dasay_u1.jpg	2025-06-12
Hypanus_say	http://www.fishbase.se/images/species/Dasay_u2.jpg	2025-06-12
Hypanus_say	https://farm8.staticflickr.com/7165/6852821225_133409b7c9.jpg	2025-06-12
Hypanus_say	https://static.inaturalist.org/photos/4140320/medium.jpg?1467254623	2025-06-12

Hypnos_monopterygius	http://fishesofaustralia.net.au/images/image/HypnosMonoptJohnTurnbull.jpg	2025-06-12
Hypnos_monopterygius	http://www.fishbase.se/images/species/Hymon_u0.jpg	2025-06-12
Hypnos_monopterygius	http://www.fishbase.se/images/species/Hymon_u1.jpg	2025-06-12
Hypnos_monopterygius	https://static.inaturalist.org/photos/5872733/medium.jpg?1482561157	2025-06-12
Hypnos_monopterygius	https://static.inaturalist.org/photos/5872735/medium.jpg?1482561247	2025-06-12
Insentiraja_laxipella	http://fishesofaustralia.net.au/images/image/InsentirajaLaxipellaCSIRO.jpg	2025-06-12
Insentiraja_subtilispinosa	http://fishesofaustralia.net.au/images/image/InsentirajaSubtilispinCSIRO.jpg	2025-06-12
Insentiraja_subtilispinosa	http://fishesofaustralia.net.au/Images/Image/InsentirajaSubtilispinosaCSIRO.jpg	2025-06-12
Irolita_westraliensis	http://shark-references.com/images/species/Irolita_westraliensis_first.jpg	2025-06-12
Irolita_westraliensis	http://shark-references.com/images/species/Irolita_westraliensis_main.jpg	2025-06-12
Leucoraja_circularis	http://www.boldsystems.org/pics/_w300/NBMF/Leuc_circ_ZMUB_22857%2B1481124224.jpg	2025-06-12
Leucoraja_circularis	http://www.boldsystems.org/pics/_w300/NBMF/Leuc_circ_ZMUB_23305%2B1481124224.jpg	2025-06-12
Leucoraja_circularis	http://www.boldsystems.org/pics/_w300/RNEZ/RNEZ243D%2B1265326376.jpg	2025-06-12
Leucoraja_circularis	http://www.boldsystems.org/pics/ELAME/6C05C1%2B1318363190.JPG	2025-06-12
Leucoraja_circularis	http://www.boldsystems.org/pics/ELAME/IMG_2383%2B1260411112.JPG	2025-06-12
Leucoraja_circularis	http://www.boldsystems.org/pics/ELAME/IMG_2388%2B1260411102.JPG	2025-06-12
Leucoraja_circularis	http://www.boldsystems.org/pics/RNEZ/RNEZ252D%2B1265327960.jpg	2025-06-12
Leucoraja_erinacea	http://www.boldsystems.org/pics/SCAFB/07-037%2B1182260296.JPG	2025-06-12
Leucoraja_erinacea	http://www.boldsystems.org/pics/SCAFB/07-259%2B1214601398.JPG	2025-06-12
Leucoraja_erinacea	http://www.boldsystems.org/pics/SCAFB/08-143%2B1235403132.jpg	2025-06-12
Leucoraja_erinacea	http://www.boldsystems.org/pics/SCFAC/06-226_15-05-08%2B1208262832.jpg	2025-06-12
Leucoraja_erinacea	http://www.fishbase.se/images/species/Leeri_u0.jpg	2025-06-12
Leucoraja_erinacea	http://www.fishbase.se/tools/UploadPhoto/uploads/P5260070.JPG	2025-06-12
Leucoraja_erinacea	https://static.inaturalist.org/photos/6182500/medium.jpg?1486091997	2025-06-12
Leucoraja_erinacea	https://teacheratsea.files.wordpress.com/2014/09/little-skate-leucoraja-erinacea.jpg	2025-06-12
Leucoraja_fullonica	http://www.boldsystems.org/pics/ELAME/d000674%2B1260411082.JPG	2025-06-12
Leucoraja_fullonica	http://www.boldsystems.org/pics/NBMF/Leuc_full_ZMUB_22856%2B1481124226.jpg	2025-06-12
Leucoraja_fullonica	http://www.fishbase.se/images/species/Leful_m0.jpg	2025-06-12
Leucoraja_fullonica	http://www.fishbase.se/tools/UploadPhoto/uploads/Leucorajafullonica2x.jpg	2025-06-12
Leucoraja_fullonica	http://www.fishbase.se/tools/UploadPhoto/uploads/shagreenray3x.jpg	2025-06-12
Leucoraja_garmani	http://shark-references.com/images/species/Leucoraja%20garmani%20PE11-9-EPI-A%20(1).jpg	2025-06-12
Leucoraja_garmani	http://www.boldsystems.org/pics/CBPM/COB101%2B1306432228.JPG	2025-06-12
Leucoraja_garmani	http://www.boldsystems.org/pics/SCAFB/07-083%2B1183485686.jpg	2025-06-12
Leucoraja_garmani	http://www.fishbase.se/images/species/Legar_u0.jpg	2025-06-12
Leucoraja_garmani	http://www.fishbase.se/images/species/Legar_u1.jpg	2025-06-12
Leucoraja_lentiginosa	http://www.fishbase.se/images/species/Lelen_u6.jpg	2025-06-12
Leucoraja_melitensis	http://www.boldsystems.org/pics/ELAME/d000723%2B1260411110.JPG	2025-06-12
Leucoraja_melitensis	http://www.boldsystems.org/pics/ELAME/d000724%2B1260411078.JPG	2025-06-12
Leucoraja_naevus	http://www.boldsystems.org/pics/BNSF/MT02832%2B1326223746.jpg	2025-06-12
Leucoraja_naevus	http://www.boldsystems.org/pics/BNSF/MT02833%2B1326223748.jpg	2025-06-12
Leucoraja_naevus	http://www.boldsystems.org/pics/BNSF/MT02836%2B1326223756.jpg	2025-06-12
Leucoraja_naevus	http://www.boldsystems.org/pics/BNSFI/MT04140%2B1375216124.jpg	2025-06-12
Leucoraja_naevus	http://www.boldsystems.org/pics/RNEZ/RNEZ231D%2B1265328798.jpg	2025-06-12

Leucoraja_naevus	http://www.fishbase.se/images/species/Lenae_f0.jpg	2025-06-12
Leucoraja_naevus	http://www.fishbase.se/images/species/Lenae_u0.jpg	2025-06-12
Leucoraja_naevus	http://www.fishbase.se/images/species/Lenae_u1.jpg	2025-06-12
Leucoraja_naevus	http://www.fishbase.se/tools/UploadPhoto/uploads/RAJNAE.JPG	2025-06-12
Leucoraja_ocellata	http://www.boldsystems.org/pics/SCAFB/08-144%2B1235403134.jpg	2025-06-12
Leucoraja_ocellata	http://www.boldsystems.org/pics/SCFAC/06-212%2B1144698184.JPG	2025-06-12
Leucoraja_ocellata	http://www.boldsystems.org/pics/SCFAC/06-875%2B1181079736.JPG	2025-06-12
Leucoraja_ocellata	http://www.fishbase.se/images/species/Leoce_m1.jpg	2025-06-12
Leucoraja_wallacei	http://www.boldsystems.org/pics/ELAME/d001542%2B1260411094.JPG	2025-06-12
Leucoraja_wallacei	http://www.boldsystems.org/pics/ELAME/d001543%2B1260411092.JPG	2025-06-12
Leucoraja_wallacei	http://www.boldsystems.org/pics/ELAME/d001619%2B1260411084.JPG	2025-06-12
Leucoraja_wallacei	http://www.boldsystems.org/pics/ELAME/d001620%2B1260411106.JPG	2025-06-12
Leucoraja_wallacei	http://www.boldsystems.org/pics/ELAME/d001625%2B1260411074.JPG	2025-06-12
Leucoraja_wallacei	http://www.fishbase.se/images/species/Lewal_u1.jpg	2025-06-12
Leucoraja_wallacei	http://www.fishbase.se/images/species/Lewal_u2.jpg	2025-06-12
Maculabatis_ambigua	http://shark-references.com/images/species/Maculabatis-ambigua_main.jpg	2025-06-12
Maculabatis_arabica	http://shark-references.com/images/species/Maculabatis_arabica_holo.jpg	2025-06-12
Maculabatis_astra	http://shark-references.com/images/species/Himantura_astra_first.jpg	2025-06-12
Maculabatis_astra	http://shark-references.com/images/species/Himantura_astra_main.jpg	2025-06-12
Maculabatis_astra	http://www.fishbase.se/photos/workimagethumb.php?s=http://www.fishbase.se/tools/UploadPhoto/uploads/1477932799_162.158.166.130.jpg&w=600	2025-06-12
Maculabatis_gerrardi	http://shark-references.com/images/species/15483_Higer_u0.jpg	2025-06-12
Maculabatis_gerrardi	http://shark-references.com/images/species/15483_Higer_u1.jpg	2025-06-12
Maculabatis_gerrardi	http://www.boldsystems.org/pics/COFPL/COFJAU148%2B1457543196.JPG	2025-06-12
Maculabatis_gerrardi	http://www.boldsystems.org/pics/FRIB/37_H.gerradi%2B1344643260.JPG	2025-06-12
Maculabatis_gerrardi	http://www.boldsystems.org/pics/INELA/Himantura_gerrardi%2B1312837868.jpg	2025-06-12
Maculabatis_gerrardi	http://www.fishbase.se/images/species/Higer_u4.jpg	2025-06-12
Maculabatis_pastinacoides	http://www.fishbase.se/images/species/Hipas_u0.jpg	2025-06-12
Maculabatis_randalli	http://www.fishbase.se/images/species/Hiran_u0.jpg	2025-06-12
Maculabatis_randalli	http://www.fishbase.se/photos/workimagethumb.php?s=http://www.fishbase.se/tools/UploadPhoto/uploads/1410333372_118.103.236.195.jpg&w=600	2025-06-12
Maculabatis_toshi	http://fishesofaustralia.net.au/images/image/HimanturaToshiCSIRO.jpg	2025-06-12
Malacoraja_senta	http://www.fishbase.se/images/species/Masen_u1.jpg	2025-06-12
Megatrygon_microps	http://fishesofaustralia.net.au/Images/Image/DasyatisMicropsCSIRO.jpg	2025-06-12
Mobula_alfredi	http://fishesofaustralia.net.au/images/image/MantaAlfrediLCouterier.jpg	2025-06-12
Mobula_alfredi	http://shark-references.com/images/species/Manta_alfredi_Csilla_Ari%20(5).JPG	2025-06-12
Mobula_alfredi	http://shark-references.com/images/species/Manta_alfredi_Csilla_Ari%20(6).JPG	2025-06-12
Mobula_birostris	http://fishesofaustralia.net.au/Images/Image/MantaBirostrisCSIRO.jpg	2025-06-12
Mobula_birostris	http://fishesofaustralia.net.au/images/image/MantaBirostrisThailand4.jpg	2025-06-12
Mobula_birostris	http://shark-references.com/images/species/Manta-birostris_CsillaAri.jpg	2025-06-12
Mobula_birostris	http://shark-references.com/images/species/Mantabirostris3.jpg	2025-06-12
Mobula_birostris	http://shark-references.com/images/species/Mantabirostris6.jpg	2025-06-12
Mobula_hypostoma	http://shark-references.com/images/species/Atlantic_Mobula_Ray_022.jpg	2025-06-12
Mobula_hypostoma	http://www.boldsystems.org/pics/FLBAR/FWRI_02514_0466%2B1484925234.JPG	2025-06-12
Mobula_hypostoma	http://www.fishbase.se/images/species/Mohyp_u0.jpg	2025-06-12

Mobula_hypostoma	https://farm9.staticflickr.com/8097/8433440609_e5305e422a.jpg	2025-06-12
Mobula_kuhlii	http://www.fishbase.se/photos/workimagethumb.php?s=http://www.fishbase.se/tools/UploadPhoto/uploads/1374152763_182.178.16.68.jpg&w=600	2025-06-12
Mobula_kuhlii	http://www.fishbase.se/photos/workimagethumb.php?s=http://www.fishbase.se/tools/UploadPhoto/uploads/1375141838_182.178.64.14.jpg&w=600	2025-06-12
Mobula_kuhlii	http://www.fishbase.se/photos/workimagethumb.php?s=http://www.fishbase.se/tools/UploadPhoto/uploads/1380915703_182.178.28.52.jpg&w=600	2025-06-12
Mobula_kuhlii	http://www.fishbase.se/tools/UploadPhoto/uploads/1372522396_182.178.67.11.jpg	2025-06-12
Mobula_kuhlii	https://static.inaturalist.org/photos/1975173/medium.jpg?1444749763	2025-06-12
Mobula_mobular	http://shark-references.com/images/species/Mobula_eregoodootenkee_complete.jpg	2025-06-12
Mobula_mobular	http://shark-references.com/images/species/Mobula_mobular.jpg	2025-06-12
Mobula_mobular	http://www.fishbase.se/images/species/Momob_u2.jpg	2025-06-12
Mobula_mobular	http://www.fishbase.se/photos/workimagethumb.php?s=http://www.fishbase.se/tools/UploadPhoto/uploads/1423059809_86.205.57.96.jpg&w=600	2025-06-12
Mobula_mobular	https://farm4.staticflickr.com/3064/2992407025_1ff0425c56.jpg	2025-06-12
Mobula_munkiana	https://shark-references.com/images/species/6004.jpg	2025-06-12
Mobula_munkiana	https://static.inaturalist.org/photos/299011104/medium.jpg	2025-06-12
Mobula_tarapacana	http://www.fishbase.se/images/species/Motar_u0.jpg	2025-06-12
Mobula_tarapacana	http://www.fishbase.se/images/species/Motar_u1.jpg	2025-06-12
Mobula_tarapacana	http://www.fishbase.se/photos/workimagethumb.php?s=http://www.fishbase.se/tools/UploadPhoto/uploads/1466725269_188.114.102.249.jpg&w=600	2025-06-12
Mobula_tarapacana	http://www.fishbase.se/tools/UploadPhoto/uploads/1347121502_62.178.129.238.jpg	2025-06-12
Mobula_thurstoni	http://fishesofaustralia.net.au/Images/Image/MobulaThrustoniCSIRO.jpg	2025-06-12
Mobula_thurstoni	http://www.fishbase.se/images/species/Mothu_m0.jpg	2025-06-12
Mobula_thurstoni	http://www.fishbase.se/photos/workimagethumb.php?s=http://www.fishbase.se/tools/UploadPhoto/uploads/1379089534_182.178.113.1.jpg&w=600	2025-06-12
Mobula_thurstoni	http://www.fishbase.se/photos/workimagethumb.php?s=http://www.fishbase.se/tools/UploadPhoto/uploads/1379089564_182.178.113.1.jpg&w=600	2025-06-12
Mobula_thurstoni	http://www.fishbase.se/photos/workimagethumb.php?s=http://www.fishbase.se/tools/UploadPhoto/uploads/1379089745_182.178.113.1.jpg&w=600	2025-06-12
Myliobatis_aquila	http://www.boldsystems.org/pics/EFBD/BPS1920A%2B1315607340.JPG	2025-06-12
Myliobatis_aquila	http://www.boldsystems.org/pics/ELAME/05MB09%2B1318363074.jpg	2025-06-12
Myliobatis_aquila	http://www.boldsystems.org/pics/ELAME/09MB01%2B1318363074.JPG	2025-06-12
Myliobatis_aquila	http://www.boldsystems.org/pics/ELAMO/el412%2B1322850530.jpg	2025-06-12
Myliobatis_aquila	http://www.boldsystems.org/pics/HVDBF/DSC01021%2B1354883678.jpg	2025-06-12
Myliobatis_aquila	http://www.fishbase.se/images/species/Myaqu_u2.jpg	2025-06-12
Myliobatis_aquila	http://www.fishbase.se/tools/UploadPhoto/uploads/Aquila_di_mare_Myliobatis_aquila_Genova_7178_93.jpg	2025-06-12
Myliobatis_aquila	http://www.fishbase.se/tools/UploadPhoto/uploads/myliobatis_aquila_CC_marineland01.jpg	2025-06-12
Myliobatis_aquila	http://www.fishbase.se/tools/UploadPhoto/uploads/P1013300.jpg	2025-06-12
Myliobatis_aquila	http://www.fishbase.se/tools/UploadPhoto/uploads/Stingray2.jpg	2025-06-12
Myliobatis_californicus	http://www.fishbase.se/images/species/Mycal_u0.jpg	2025-06-12
Myliobatis_californicus	http://www.fishbase.se/images/species/Mycal_u1.jpg	2025-06-12
Myliobatis_californicus	http://www.fishbase.se/images/species/Mycal_u2.jpg	2025-06-12
Myliobatis_californicus	https://farm1.staticflickr.com/30/41548389_832c197e9e.jpg	2025-06-12
Myliobatis_chilensis	http://www.fishbase.se/images/species/Mychi_j0.jpg	2025-06-12
Myliobatis_freminvillei	http://shark-references.com/images/species/Myliobatis%20freminvillei%20S09-C%20BEST.jpg	2025-06-12
Myliobatis_freminvillei	http://www.fishbase.se/images/species/Myfre_u0.jpg	2025-06-12
Myliobatis_freminvillei	http://www.fishbase.se/images/species/Myfre_u1.jpg	2025-06-12
Myliobatis_goodei	http://shark-references.com/images/species/Myliobatus%20goodei%20Imm%20Male%20N06-EPI-B%20%20BEST.jpg	2025-06-12
Myliobatis_goodei	http://shark-references.com/images/species/thumbnail/Myliobatus-goodei.jpg	2025-06-12
Myliobatis_goodei	http://www.boldsystems.org/pics/CCB/MT20%2B1355343502.JPG	2025-06-12

Myliobatis_goodei	http://www.boldsystems.org/pics/CCB/MT5%2B1346448068.JPG	2025-06-12
Myliobatis_goodei	http://www.boldsystems.org/pics/FARGB/UNMDP_DI_105%2B1303442640.jpg	2025-06-12
Myliobatis_goodei	http://www.boldsystems.org/pics/FARGB/UNMDP-DI_0493_dorsal%2B1312465226.JPG	2025-06-12
Myliobatis_goodei	http://www.boldsystems.org/pics/FARGB/UNMDP-DI_0497_dorsal%2B1312465232.JPG	2025-06-12
Myliobatis_goodei	http://www.boldsystems.org/pics/FARGB/UNMDP-DI_080%2B1312408724.JPG	2025-06-12
Myliobatis_hamlyni	http://fishesofaustralia.net.au/images/image/MyliobatisHamlyniKG.jpg	2025-06-12
Myliobatis_hamlyni	http://shark-references.com/images/species/Myliobatis_hamlyni.bmp	2025-06-12
Myliobatis_longirostris	http://shark-references.com/images/species/Myliobatis_longirostris.jpg	2025-06-12
Myliobatis_peruvianus	https://shark-references.com/images/species/M_peruvianus_peru.jpg	2025-06-12
Myliobatis_ridens	http://www.boldsystems.org/pics/CCB/MT13%2B1355343500.JPG	2025-06-12
Myliobatis_ridens	http://www.boldsystems.org/pics/FARGB/UNMDP-DI_0507_dorsal%2B1312465240.JPG	2025-06-12
Myliobatis_ridens	http://www.boldsystems.org/pics/FARGB/UNMDP-DI_0528_dorsal%2B1312465256.JPG	2025-06-12
Myliobatis_tenuicaudatus	http://fishesofaustralia.net.au/images/image/MyliobatAustralRK.jpg	2025-06-12
Myliobatis_tenuicaudatus	http://www.fishbase.se/images/species/Myten_m0.jpg	2025-06-12
Myliobatis_tenuicaudatus	https://static.inaturalist.org/photos/1200347/medium.?1413263052	2025-06-12
Myliobatis_tenuicaudatus	https://static.inaturalist.org/photos/1441659/medium.jpg?1419465585	2025-06-12
Narcine_baliensis	http://shark-references.com/images/species/thumbnail/Narcine_baliensis_main_para.jpg	2025-06-12
Narcine_baliensis	http://shark-references.com/images/species/thumbnail/Narcine_baliensis_para.jpg	2025-06-12
Narcine_bancroftii	http://shark-references.com/images/species/Leucistic_Narcine.jpg	2025-06-12
Narcine_bancroftii	http://www.boldsystems.org/pics/FWRI/FWRI00277_Narcine_bancroftii%2B1336180112.JPG	2025-06-12
Narcine_bancroftii	http://www.fishbase.se/images/species/Naban_u0.jpg	2025-06-12
Narcine_bancroftii	http://www.fishbase.se/tools/UploadPhoto/uploads/N.bancroftii.jpg	2025-06-12
Narcine_brasiliensis	http://www.fishbase.se/images/species/Nabra_j0.jpg	2025-06-12
Narcine_brasiliensis	http://www.fishbase.se/images/species/Nabra_u0.jpg	2025-06-12
Narcine_brasiliensis	http://www.fishbase.se/images/species/Nabra_u1.jpg	2025-06-12
Narcine_brasiliensis	http://www.fishbase.se/images/species/Nabra_u6.jpg	2025-06-12
Narcine_brasiliensis	http://www.fishbase.se/tools/UploadPhoto/uploads/Nbrasiliesis.jpg	2025-06-12
Narcine_brasiliensis	https://static.inaturalist.org/photos/5137736/medium.jpeg?1475783358	2025-06-12
Narcine_brevilabiata	http://www.boldsystems.org/pics/CFCS/P1000564%2B1214072986.JPG	2025-06-12
Narcine_brevilabiata	http://www.fishbase.se/photos/workimagethumb.php?s=http://www.fishbase.se/tools/UploadPhoto/uploads/1445915831_125.26.188.203.jpg&w=600	2025-06-12
Narcine_entemedor	http://www.boldsystems.org/pics/SEMAR/SEMAR-161%2B1274311118.jpg	2025-06-12
Narcine_entemedor	http://www.discoverlife.org/IM/I_RR/0001/640/Narcine_entemedor,I_RR155.jpg	2025-06-12
Narcine_entemedor	http://www.fishbase.se/images/species/Naent_u0.jpg	2025-06-12
Narcine_entemedor	http://www.fishbase.se/images/species/Naent_u1.jpg	2025-06-12
Narcine_entemedor	http://www.fishbase.se/photos/workimagethumb.php?s=http://www.fishbase.se/tools/UploadPhoto/uploads/1430323399_173.19.96.111.jpg&w=600	2025-06-12
Narcine_entemedor	https://static.inaturalist.org/photos/7172736/medium.jpeg?1492392795	2025-06-12
Narcinops_lasti	http://fishesofaustralia.net.au/images/image/NarcineLastiCSIRO.jpg	2025-06-12
Narcine_leoparda	http://shark-references.com/images/species/N.%20leoparda%20Ecuador_Carrera-Fern%C3%A1ndez%202.jpg	2025-06-12
Narcine_lingula	http://www.fishbase.se/images/species/Nalin_u0.jpg	2025-06-12
Narcine_lingula	http://www.fishbase.se/images/species/Nalin_u1.jpg	2025-06-12
Narcine_prodorsalis	http://www.fishbase.se/images/species/Napro_u0.jpg	2025-06-12
Narcine_prodorsalis	http://www.fishbase.se/photos/workimagethumb.php?s=http://www.fishbase.se/tools/UploadPhoto/uploads/1486542235_172.68.106.90.jpg&w=600	2025-06-12
Narcinops_tasmaniensis	http://fishesofaustralia.net.au/Images/Image/NarcineTasmaniensisCSIRO.jpg	2025-06-12

Narcinops_tasmaniensis	http://shark-references.com/images/species/Narcine_tasmaniensis.jpg	2025-06-12
Narcinops_tasmaniensis	http://www.fishbase.se/images/species/Natas_u1.jpg	2025-06-12
Narcinops_tasmaniensis	http://www.fishbase.se/tools/UploadPhoto/uploads/1403835392_114.76.64.149.jpg	2025-06-12
Narcinops_tasmaniensis	http://www.fishbase.se/tools/UploadPhoto/uploads/1403835516_114.76.64.149.jpg	2025-06-12
Narcine_timlei	http://www.fishbase.se/images/species/Natim_u1.jpg	2025-06-12
Narcine_timlei	http://www.fishbase.se/tools/UploadPhoto/uploads/Narcinetimlei.JPG	2025-06-12
Narcine_timlei	https://www.researchgate.net/profile/R_K_Manna/publication/301682351/figure/fig1/AS:355758324633600@1461830802401/Figure-2-Narcine-timlei-female-recorded-from-Chilika-lagoon-a-Dorsal-view-b.png	2025-06-12
Narcine_vermiculata	http://www.boldsystems.org/pics/SEMAR/SEMAR-159%2B1274311098.jpg	2025-06-12
Narcine_vermiculata	http://www.fishbase.se/images/species/Naver_u0.jpg	2025-06-12
Narcine_vermiculata	http://www.fishbase.se/images/species/Naver_u1.jpg	2025-06-12
Narcinops_nelsoni	http://shark-references.com/images/species/Narcine_nelsoni_first.jpg	2025-06-12
Narcinops_ornata	http://fishesofaustralia.net.au/images/image/NarcineOrnataSIRO.jpg	2025-06-12
Narcinops_westraliensis	http://fishesofaustralia.net.au/images/image/NarcineWestraliensisCSIRO.jpg	2025-06-12
Narcinops_westraliensis	http://www.fishbase.se/images/species/Nawes_u0.jpg	2025-06-12
Narke_capensis	http://www.fishbase.se/images/species/Nacap_u0.jpg	2025-06-12
Narke_dipterygia	http://www.fishbase.se/images/species/Nadip_u0.jpg	2025-06-12
Narke_dipterygia	http://www.fishbase.se/photos/workimagethumb.php?s=http://www.fishbase.se/tools/UploadPhoto/uploads/1382007499_182.178.61.112.jpg&w=600	2025-06-12
Narke_dipterygia	http://www.fishbase.se/photos/workimagethumb.php?s=http://www.fishbase.se/tools/UploadPhoto/uploads/1382007635_182.178.61.112.jpg&w=600	2025-06-12
Narke_dipterygia	http://www.fishbase.se/photos/workimagethumb.php?s=http://www.fishbase.se/tools/UploadPhoto/uploads/1382008316_182.178.61.112.jpg&w=600	2025-06-12
Narke_dipterygia	http://www.fishbase.se/tools/UploadPhoto/uploads/Narkedipterygia.JPG	2025-06-12
Narke_japonica	http://www.fishbase.se/images/species/Najap_u0.jpg	2025-06-12
Narke_japonica	http://www.fishbase.se/tools/UploadPhoto/uploads/NJ_front.gif	2025-06-12
Neoraja_caerulea	http://www.fishbase.se/images/species/Necae_m0.jpg	2025-06-12
Neoraja_iberica	http://www.fishbase.se/images/species/Neibe_f0.gif	2025-06-12
Neoraja_iberica	http://www.fishbase.se/images/species/Neibe_j0.gif	2025-06-12
Neoraja_iberica	http://www.fishbase.se/images/species/Neibe_m0.gif	2025-06-12
Neotrygon_annotata	http://fishesofaustralia.net.au/images/image/NeotrygonAnnotatCSIRO.jpg	2025-06-12
Neotrygon_australiae	http://fishesofaustralia.net.au/images/image/DasyatisKuhlii2DH.jpg	2025-06-12
Neotrygon_caeruleopunctata	http://shark-references.com/images/species/Neotrygon_caeruleopunctata.jpg	2025-06-12
Neotrygon_kuhlii	http://shark-references.com/images/species/800px-Bluespotted_stingray_papua_new_guinea.jpg	2025-06-12
Neotrygon_kuhlii	http://shark-references.com/images/species/Neotrygon%20kuhlii%20_poeschl.jpg	2025-06-12
Neotrygon_kuhlii	http://www.boldsystems.org/pics/ANCC/BPS0655A%2B1174071366.JPG	2025-06-12
Neotrygon_kuhlii	http://www.boldsystems.org/pics/INELA/Neotrygon_Kuhlii_01%2B1315335076.jpg	2025-06-12
Neotrygon_kuhlii	http://www.boldsystems.org/pics/SERIP/DASsab_P1040123d%2B1468762156.jpg	2025-06-12
Neotrygon_kuhlii	http://www.boldsystems.org/pics/SERIP/DASsab_P1040125%2B1465570052.jpg	2025-06-12
Neotrygon_kuhlii	http://www.fishbase.se/images/species/Dakuh_u0.jpg	2025-06-12
Neotrygon_kuhlii	http://www.fishbase.se/images/species/Dakuh_u2.jpg	2025-06-12
Neotrygon_kuhlii	http://www.fishbase.se/images/species/Dakuh_u3.jpg	2025-06-12
Neotrygon_kuhlii	http://www.fishbase.se/images/species/Dakuh_u4.jpg	2025-06-12
Neotrygon_leylandi	http://fishesofaustralia.net.au/images/image/NeotrygonLeylandiCSIRO.jpg	2025-06-12
Neotrygon_leylandi	http://www.fishbase.se/photos/workimagethumb.php?s=http://www.fishbase.se/tools/UploadPhoto/uploads/1383813599_118.175.90.164.jpg&w=600	2025-06-12
Neotrygon_ningalooensis	http://fishesofaustralia.net.au/Images/Image/NeotrygonNingalooensisCSIRO.jpg	2025-06-12

Neotrygon_ningalooensis	http://fishesofaustralia.net.au/images/image/NeotrygonNingalooensisRLS.jpg	2025-06-12
Neotrygon_picta	http://fishesofaustralia.net.au/images/image/NeotrygonPictaCSIRO.jpg	2025-06-12
Neotrygon_picta	http://shark-references.com/images/species/Neotrygon_picta_main.jpg	2025-06-12
Neotrygon_picta	http://www.fishbase.se/images/species/Nepic_u0.jpg	2025-06-12
Neotrygon_trigonoides	http://fishesofaustralia.net.au/images/image/NeotrygTrigonoid2AnneHoggett.jpg	2025-06-12
Neotrygon_trigonoides	http://fishesofaustralia.net.au/Images/Image/NeotrygTrigonoid4AnneHoggett.jpg	2025-06-12
Neotrygon_trigonoides	http://shark-references.com/images/species/Dkuh%20NC%2020080816%20p.%20CSIRO%20P1110513.jpg	2025-06-12
Neotrygon_trigonoides	http://shark-references.com/images/species/Dkuh%20NC%2020090816%20P1000851.jpg	2025-06-12
Neotrygon_trigonoides	http://shark-references.com/images/species/Dkuh%20NC%20MNHN%202009-0823%20P1000106.jpg	2025-06-12
Neotrygon_trigonoides	http://shark-references.com/images/species/Neotrygon_trigonoides_holotype.jpg	2025-06-12
Notoraja_alisae	http://www.boldsystems.org/pics/ANCC/BPS0306A%2B1024296122.jpg	2025-06-12
Notoraja_azurea	http://fishesofaustralia.net.au/images/image/NotorajaAzureaCSIRO.jpg	2025-06-12
Notoraja_azurea	http://shark-references.com/images/species/Notoraja_azurea_main.jpg	2025-06-12
Notoraja_hirticauda	http://fishesofaustralia.net.au/images/image/NotorajaHirticaudaCSIRO.jpg	2025-06-12
Notoraja_lira	http://shark-references.com/images/species/Notoraja_lira_first.jpg	2025-06-12
Notoraja_sapphira	http://www.fishbase.se/images/species/Nosap_m0.jpg	2025-06-12
Notoraja_sticta	http://shark-references.com/images/species/Notoraja_sticta_first.jpg	2025-06-12
Notoraja_sticta	http://shark-references.com/images/species/Notoraja_sticta_main.jpg	2025-06-12
Okamejei_boesemani	http://shark-references.com/images/species/Okamejei-boesemani_main.jpg	2025-06-12
Okamejei_cairae	http://www.fishbase.se/images/species/Okcai_u0.jpg	2025-06-12
Okamejei_hollandi	http://www.fishbase.se/photos/workimagethumb.php?s=http://www.fishbase.se/tools/UploadPhoto/uploads/1480411165_172.68.107.103.jpg&w=600	2025-06-12
Okamejei_kenojei	http://www.fishbase.se/images/species/Okken_u0.jpg	2025-06-12
Okamejei_leptoura	http://fishesofaustralia.net.au/images/image/OkamejeiLeptouraCSIRO.jpg	2025-06-12
Okamejei_leptoura	http://shark-references.com/images/species/Okamejei_leptoura_first.jpg	2025-06-12
Okamejei_leptoura	http://shark-references.com/images/species/Okamejei_leptoura_main.jpg	2025-06-12
Okamejei_leptoura	http://shark-references.com/images/species/Okamejei_leptoura_paratype.jpg	2025-06-12
Orbiraja_jensenae	http://shark-references.com/images/species/Okamejei-jensenae_first.jpg	2025-06-12
Orbiraja_jensenae	http://www.fishbase.se/images/species/Okjen_u0.jpg	2025-06-12
Orbiraja_jensenae	http://www.fishbase.se/photos/workimagethumb.php?s=http://www.fishbase.se/tools/UploadPhoto/uploads/1374050515_182.52.68.240.jpg&w=600	2025-06-12
Orbiraja_jensenae	http://www.fishbase.se/photos/workimagethumb.php?s=http://www.fishbase.se/tools/UploadPhoto/uploads/1383627451_182.52.68.117.jpg&w=600	2025-06-12
Orbiraja_jensenae	http://www.fishbase.se/photos/workimagethumb.php?s=http://www.fishbase.se/tools/UploadPhoto/uploads/1383636863_182.52.68.117.jpg&w=600	2025-06-12
Orbiraja_jensenae	http://www.fishbase.se/photos/workimagethumb.php?s=http://www.fishbase.se/tools/UploadPhoto/uploads/1441855063_101.109.72.177.jpg&w=600	2025-06-12
Orbiraja_jensenae	http://www.fishbase.se/photos/workimagethumb.php?s=http://www.fishbase.se/tools/UploadPhoto/uploads/1441855427_101.109.72.177.jpg&w=600	2025-06-12
Orbiraja_jensenae	http://www.fishbase.se/photos/workimagethumb.php?s=http://www.fishbase.se/tools/UploadPhoto/uploads/1441855880_101.109.72.177.jpg&w=600	2025-06-12
Paratrygon_aiereba	http://shark-references.com/images/species/Paratrygon_aiereba_INPA40507.jpg	2025-06-12
Paratrygon_aiereba	http://www.fishbase.se/tools/UploadPhoto/uploads/DSCF0516.JPG	2025-06-12
Pastinachus_ater	http://fishesofaustralia.net.au/Images/Image/PastinachusAterCSIRO.jpg	2025-06-12
Pastinachus_ater	http://fishesofaustralia.net.au/images/image/PastinachusAtrusAndyALewis.jpg	2025-06-12
Pastinachus_ater	http://www.boldsystems.org/pics/ANCC/BPS0660A%2B1174326970.JPG	2025-06-12
Pastinachus_ater	http://www.boldsystems.org/pics/ANCC/BPS2123A%2B1336867720.JPG	2025-06-12
Pastinachus_ater	http://www.boldsystems.org/pics/JTFR/PKTN60%2B1490080966.jpg	2025-06-12
Pastinachus_ater	http://www.boldsystems.org/pics/JTFR/PKTN73%2B1490080993.jpg	2025-06-12
Pastinachus_ater	http://www.fishbase.se/images/species/Paatr_u0.jpg	2025-06-12

Pastinachus_gracilicaudus	http://shark-references.com/images/species/Pastinachus-gracilicaudus_first.jpg	2025-06-12
Pastinachus_gracilicaudus	http://shark-references.com/images/species/Pastinachus-gracilicaudus_main.jpg	2025-06-12
Pastinachus_sephen	http://shark-references.com/images/species/JNC3139%20Pastinachus%20sephen%20PB270018.JPG	2025-06-12
Pastinachus_sephen	http://www.boldsystems.org/pics/INELA/Pastinachus_sephen%2B1315334348.jpg	2025-06-12
Pastinachus_sephen	http://www.fishbase.se/FishWatcher/uploads/images/Copy.jpg	2025-06-12
Pastinachus_sephen	http://www.fishbase.se/images/species/Pasep_u0.jpg	2025-06-12
Pastinachus_sephen	http://www.fishbase.se/images/species/Pasep_u1.jpg	2025-06-12
Pastinachus_sephen	http://www.fishbase.se/images/species/Pasep_u4.jpg	2025-06-12
Pastinachus_sephen	http://www.fishbase.se/photos/workimagethumb.php?s=http://www.fishbase.se/tools/UploadPhoto/uploads/1450687298_202.141.231.138.jpg&w=600	2025-06-12
Pastinachus_solocirostris	http://www.boldsystems.org/pics/KFDB/NGK54%2B1496088880.JPG	2025-06-12
Pastinachus_stellurostris	http://shark-references.com/images/species/Pastinachus_stellurostris_main.jpg	2025-06-12
Pastinachus_stellurostris	http://www.fishbase.se/images/species/Paste_u0.jpg	2025-06-12
Pateobatis_bleekeri	http://shark-references.com/images/species/Himantura%20bleekeri.jpg	2025-06-12
Pateobatis_bleekeri	http://www.fishbase.se/images/species/Hible_u2.jpg	2025-06-12
Pateobatis_bleekeri	http://www.fishbase.se/tools/UploadPhoto/uploads/1366767771_182.178.50.0.jpg	2025-06-12
Pateobatis_fai	http://fishesofaustralia.net.au/Images/Image/HimantFaiRainerKretzberg.jpg	2025-06-12
Pateobatis_fai	http://fishesofaustralia.net.au/Images/Image/HimanturaFaiCocoetJo.jpg	2025-06-12
Pateobatis_fai	http://fishesofaustralia.net.au/Images/Image/HimanturaFaiCSIRO2.jpg	2025-06-12
Pateobatis_fai	http://fishesofaustralia.net.au/images/image/HimanturFaiKirkRGastrich.jpg	2025-06-12
Pateobatis_fai	http://shark-references.com/images/species/12587_Hifai_u1.jpg	2025-06-12
Pateobatis_fai	http://www.fishbase.se/images/species/Hifai_u0.jpg	2025-06-12
Pateobatis_jenkinsii	http://fishesofaustralia.net.au/images/image/HimanturaJenkinsiiCSIRO.jpg	2025-06-12
Pateobatis_jenkinsii	http://fishesofaustralia.net.au/Images/Image/HimaturJenkinsJimGreenfield.jpg	2025-06-12
Pateobatis_uarnacoides	http://www.fishbase.se/images/species/Hiuar_u6.jpg	2025-06-12
Pavoraja_alleni	http://fishesofaustralia.net.au/images/image/PavorajaAlleniCSIRO.jpg	2025-06-12
Pavoraja_mosaica	http://fishesofaustralia.net.au/images/image/PavorajaMosaicaCSIRO.jpg	2025-06-12
Pavoraja_nitida	http://fishesofaustralia.net.au/images/image/PavorajaNitidaCSIRO.jpg	2025-06-12
Pavoraja_pseudonitida	http://fishesofaustralia.net.au/images/image/PavorajaPseudonitidaCSIRO.jpg	2025-06-12
Pavoraja_umbrosa	http://fishesofaustralia.net.au/images/image/PavorajaUmbrosaCSIRO.jpg	2025-06-12
Platyrhina_hyugaensis	http://www.fishbase.se/images/species/Plhyu_f0.jpg	2025-06-12
Platyrhina_psomadakisi	http://shark-references.com/images/species/Platyrhina_psomadakisi_paratype.jpg	2025-06-12
Platyrhina_sinensis	http://www.fishbase.se/images/species/Plsin_u2.jpg	2025-06-12
Platyrhina_sinensis	http://www.fishbase.se/photos/workimagethumb.php?s=http://www.fishbase.se/tools/UploadPhoto/uploads/1442216986_101.109.72.183.jpg&w=600	2025-06-12
Platyrhina_tangi	http://shark-references.com/images/species/Platyrhina-tangi.jpg	2025-06-12
Platyrhina_tangi	http://www.fishbase.org/images/species/Plsin_u1.jpg	2025-06-12
Platyrhina_tangi	http://www.fishbase.org/images/species/Pltan_m0.jpg	2025-06-12
Platyrhina_tangi	http://www.fishbase.org/tools/UploadPhoto/uploads/1366081428_203.218.206.45.jpg	2025-06-12
Platyrhinoidis_triseriata	http://www.fishbase.se/images/species/Pltri_u0.jpg	2025-06-12
Platyrhinoidis_triseriata	http://www.fishbase.se/images/species/Pltri_u1.jpg	2025-06-12
Plesiobatis_daviesi	http://fishesofaustralia.net.au/images/image/PlesiobatisDaviesiKG.jpg	2025-06-12
Plesiobatis_daviesi	http://www.boldsystems.org/pics/ANCC/BPS0304A%2B1024295546.jpg	2025-06-12
Plesiobatis_daviesi	http://www.fishbase.se/images/species/Pldav_m0.jpg	2025-06-12
Plesiobatis_daviesi	http://www.fishbase.se/tools/UploadPhoto/uploads/ples.jpg	2025-06-12

Potamotrygon_albimaculata	https://shark-references.com/images/species/Potamotrygon_albimaculata_main.jpg	2025-06-12
Potamotrygon_amandae	http://shark-references.com/images/species/Foto_1_Lipsker.jpg	2025-06-12
Potamotrygon_brachyura	http://www.boldsystems.org/pics/FARAN/IBSI_Ict_DI_0004%2B1385170236.JPG	2025-06-12
Potamotrygon_brachyura	http://www.boldsystems.org/pics/FARAN/IBSI_Ict_DI_0008%2B1385170238.JPG	2025-06-12
Potamotrygon_brachyura	http://www.boldsystems.org/pics/FARAN/IBSI_Ict_DI_0010%2B1385170238.JPG	2025-06-12
Potamotrygon_brachyura	http://www.boldsystems.org/pics/LARI/LAR445%2B1424792536.JPG	2025-06-12
Potamotrygon_falkneri	http://shark-references.com/images/species/Potamotrygon_falkneri.jpg	2025-06-12
Potamotrygon_falkneri	http://www.boldsystems.org/pics/CEGAR/IBSI_Ict_DI_0040%2B1415664914.JPG	2025-06-12
Potamotrygon_falkneri	http://www.fishbase.se/images/species/Pocas_u0.jpg	2025-06-12
Potamotrygon_falkneri	http://www.fishbase.se/images/species/Pofal_u0.jpg	2025-06-12
Potamotrygon_falkneri	http://www.fishbase.se/images/species/Pofal_u1.jpg	2025-06-12
Potamotrygon_falkneri	http://www.fishbase.se/tools/UploadPhoto/uploads/brazilandargentinamarch2009028.jpg	2025-06-12
Potamotrygon_henlei	http://www.fishbase.se/images/species/Pohen_u0.jpg	2025-06-12
Potamotrygon_henlei	http://www.fishbase.se/tools/UploadPhoto/uploads/IMG_0429.JPG	2025-06-12
Potamotrygon_histrix	http://www.fishbase.se/images/species/Pohis_u0.jpg	2025-06-12
Potamotrygon_histrix	http://www.fishbase.se/images/species/Pohis_u1.jpg	2025-06-12
Potamotrygon_histrix	http://www.fishbase.se/images/species/Pohys_u0.jpg	2025-06-12
Potamotrygon_histrix	http://www.fishbase.se/tools/UploadPhoto/uploads/p.hystrix.jpg	2025-06-12
Potamotrygon_histrix	https://static.inaturalist.org/photos/682948/medium.JPG?1392864591	2025-06-12
Potamotrygon_humerosa	http://shark-references.com/images/species/Potamotrygon%20humerosa_1.jpg	2025-06-12
Potamotrygon_jabuti	http://shark-references.com/images/species/Potamotrygon_jabuti_main.jpg	2025-06-12
Potamotrygon_leopoldi	http://shark-references.com/images/species/Potamotrygon_leopoldi_INPA_BR12-09B.jpg	2025-06-12
Potamotrygon_leopoldi	http://www.fishbase.se/images/species/Poleo_u0.jpg	2025-06-12
Potamotrygon_leopoldi	http://www.fishbase.se/images/species/Poleo_u1.jpg	2025-06-12
Potamotrygon_leopoldi	http://www.fishbase.se/images/species/Poleo_u3.jpg	2025-06-12
Potamotrygon_leopoldi	http://www.fishbase.se/images/species/Poleo_u5.jpg	2025-06-12
Potamotrygon_leopoldi	http://www.fishbase.se/photos/workimagethumb.php?s=http://www.fishbase.se/tools/UploadPhoto/uploads/1403709323_177.64.109.143.jpg&w=600	2025-06-12
Potamotrygon_leopoldi	http://www.fishbase.se/tools/UploadPhoto/uploads/Potamotrygon_leopoldi.jpg	2025-06-12
Potamotrygon_limai	http://shark-references.com/images/species/Potamotrygon_limai_holotype.jpg	2025-06-12
Potamotrygon_magdalenae	http://www.fishbase.se/images/species/Pomag_f0.jpg	2025-06-12
Potamotrygon_magdalenae	http://www.fishbase.se/images/species/Pomag_j0.jpg	2025-06-12
Potamotrygon_magdalenae	http://www.fishbase.se/images/species/Pomag_j1.jpg	2025-06-12
Potamotrygon_marinae	https://www.fishbase.se/images/species/Pomar_m1.jpg	2025-06-12
Potamotrygon_motoro	http://shark-references.com/images/species/Potamotrygon_motoro.jpg	2025-06-12
Potamotrygon_motoro	http://www.boldsystems.org/pics/LARI/LAR312_%5B2%5D%2B1364390950.JPG	2025-06-12
Potamotrygon_motoro	http://www.boldsystems.org/pics/LARI/LAR314_%5B2%5D%2B1364390696.JPG	2025-06-12
Potamotrygon_motoro	http://www.boldsystems.org/pics/LARI/LAR315_%5B2%5D%2B1366322252.JPG	2025-06-12
Potamotrygon_motoro	http://www.fishbase.se/images/species/Pomot_u0.jpg	2025-06-12
Potamotrygon_motoro	http://www.fishbase.se/images/species/Pomot_u2.jpg	2025-06-12
Potamotrygon_motoro	http://www.fishbase.se/images/species/Pomot_u3.jpg	2025-06-12
Potamotrygon_motoro	http://www.fishbase.se/images/species/Pomot_u4.jpg	2025-06-12
Potamotrygon_motoro	http://www.fishbase.se/images/species/Pomot_u5.jpg	2025-06-12
Potamotrygon_motoro	http://www.fishbase.se/images/species/Pomot_u6.jpg	2025-06-12

Potamotrygon_motoro	https://farm4.staticflickr.com/3237/2645987203_8e0e2beaf6.jpg	2025-06-12
Potamotrygon_motoro	https://farm7.staticflickr.com/6046/6379056973_2993b5d303.jpg	2025-06-12
Potamotrygon_orbignyi	http://www.fishbase.se/images/species/Poorb_u0.jpg	2025-06-12
Potamotrygon_orbignyi	http://www.fishbase.se/images/species/Poorb_u1.jpg	2025-06-12
Potamotrygon_orbignyi	http://www.fishbase.se/images/species/Poorb_u2.jpg	2025-06-12
Potamotrygon_orbignyi	http://www.fishbase.se/images/species/Poorb_u3.jpg	2025-06-12
Potamotrygon_orbignyi	http://www.fishbase.se/images/species/Poorb_u4.jpg	2025-06-12
Potamotrygon_orbignyi	http://www.fishbase.se/tools/UploadPhoto/uploads/porbignyi_ventredorso.jpg	2025-06-12
Potamotrygon_orbignyi	http://www.shark-references.com/images/species/Potamotrygon_orbignyi_ANSP199581_145MDW.jpg	2025-06-12
Potamotrygon_pantanensis	http://www.shark-references.com/images/species/Potamotrgon_pantanensis_first.jpg	2025-06-12
Potamotrygon_pantanensis	http://www.shark-references.com/images/species/Potamotrgon_pantanensis_main.jpg	2025-06-12
Potamotrygon_rex	https://shark-references.com/images/species/Potamotrygon-rex.jpg	2025-06-12
Potamotrygon_schroederi	http://www.fishbase.se/images/species/Posch_u0.jpg	2025-06-12
Potamotrygon_schroederi	http://www.fishbase.se/images/species/Posch_u2.jpg	2025-06-12
Potamotrygon_schroederi	http://www.fishbase.se/images/species/Posch_u3.jpg	2025-06-12
Potamotrygon_schroederi	http://www.fishbase.se/tools/UploadPhoto/uploads/p.schoerederi.jpg	2025-06-12
Potamotrygon_scobina	http://www.fishbase.se/images/species/Paaje_u0.jpg	2025-06-12
Potamotrygon_scobina	http://www.fishbase.se/images/species/Posco_u0.jpg	2025-06-12
Potamotrygon_scobina	http://www.fishbase.se/tools/UploadPhoto/uploads/P.scobina.JPG	2025-06-12
Potamotrygon_signata	http://shark-references.com/images/species/Potamotrygon_signata_main.jpg	2025-06-12
Potamotrygon_tigrina	http://shark-references.com/images/species/MUSM39978_exANSP%20182452.jpg	2025-06-12
Potamotrygon_yepezi	http://www.fishbase.se/images/species/Poyep_j0.jpg	2025-06-12
Potamotrygon_yepezi	http://www.fishbase.se/images/species/Poyep_m0.jpg	2025-06-12
Pristis_clavata	https://shark-references.com/images/species/Pristis_clavata_Kyne.jpg	2025-06-12
Pristis_clavata	https://farm5.staticflickr.com/4089/4966159001_0eb4a12318.jpg	2025-06-12
Pristis_pectinata	http://www.fishbase.org/photos/workimagethumb.php?s=http://www.fishbase.org/tools/UploadPhoto/uploads/1459798621_141.101.102.51.jpg&w=600	2025-06-12
Pristis_pectinata	http://www.fishbase.org/tools/UploadPhoto/uploads/pakawat.jpg	2025-06-12
Pristis_pristis	http://fishesofaustralia.net.au/Images/Image/PristisPristisCSIRO.jpg	2025-06-12
Pristis_pristis	http://fishesofaustralia.net.au/Images/Image/PristisPristisDaveWilson.jpg	2025-06-12
Pristis_pristis	http://fishesofaustralia.net.au/Images/Image/PristisPristisPeterKyne.jpg	2025-06-12
Pristis_pristis	http://fishesofaustralia.net.au/Images/Image/PristisPristisSFUUniversityCommunications.jpg	2025-06-12
Pristis_zijsron	http://www.fishbase.se/tools/UploadPhoto/uploads/Pesce_sega_Pristis_zijsron_Genova_6746_93.jpg	2025-06-12
Psammobatis_bergi	http://www.boldsystems.org/pics/CCB/PbM1%2B1339686050.JPG	2025-06-12
Psammobatis_bergi	http://www.boldsystems.org/pics/CCB/PbM2%2B1339686252.JPG	2025-06-12
Psammobatis_bergi	http://www.boldsystems.org/pics/CCB/PbM3%2B1339686316.JPG	2025-06-12
Psammobatis_bergi	http://www.boldsystems.org/pics/CCB/PbM4%2B1339686374.JPG	2025-06-12
Psammobatis_bergi	http://www.boldsystems.org/pics/CCB/PbM5%2B1355343474.JPG	2025-06-12
Psammobatis_bergi	http://www.boldsystems.org/pics/FARG/INIDEP-DI_0794%2B1255812908.jpg	2025-06-12
Psammobatis_bergi	http://www.boldsystems.org/pics/FARG/INIDEP-DI_0795%2B1255812682.jpg	2025-06-12
Psammobatis_bergi	http://www.boldsystems.org/pics/FARG/INIDEP-DI_0796%2B1255812354.jpg	2025-06-12
Psammobatis_bergi	http://www.boldsystems.org/pics/FARGB/UNMDP_DI_103%2B1303442612.jpg	2025-06-12
Psammobatis_bergi	http://www.boldsystems.org/pics/FWFA/UNMDP_DI_1029%2B1308246576.JPG	2025-06-12
Psammobatis_bergi	http://www.fishbase.se/images/species/Psber_f0.jpg	2025-06-12

Psammobatis_bergi	http://www.fishbase.se/images/species/Psber_m0.jpg	2025-06-15
Psammobatis_extenta	http://www.boldsystems.org/pics/CCB/RM-006%2B1355343522.JPG	2025-06-15
Psammobatis_extenta	http://www.boldsystems.org/pics/CCB/RM-007%2B1355343524.JPG	2025-06-15
Psammobatis_extenta	http://www.boldsystems.org/pics/CCB/RM-008%2B1355343526.JPG	2025-06-15
Psammobatis_extenta	http://www.boldsystems.org/pics/FARG/INIDEP-DI_0797%2B1254767536.jpg	2025-06-15
Psammobatis_extenta	http://www.fishbase.se/images/species/Psext_u0.jpg	2025-06-15
Psammobatis_lentiginosa	http://www.boldsystems.org/pics/CEGAR/CEGAR_DI_044b%2B1307720788.JPG	2025-06-15
Psammobatis_lentiginosa	http://www.boldsystems.org/pics/FARG/INIDEP-DI_0347%2B1160757518.jpg	2025-06-15
Psammobatis_lentiginosa	http://www.boldsystems.org/pics/FARG/INIDEP-DI_0348%2B1160758224.jpg	2025-06-15
Psammobatis_lentiginosa	http://www.boldsystems.org/pics/FARG/INIDEP-DI_0349%2B1160758678.jpg	2025-06-15
Psammobatis_lentiginosa	http://www.boldsystems.org/pics/FARG/INIDEP-DI_0350%2B1160759248.jpg	2025-06-15
Psammobatis_lentiginosa	http://www.boldsystems.org/pics/FARG/INIDEP-DI_0462%2B1196178292.JPG	2025-06-15
Psammobatis_lentiginosa	http://www.boldsystems.org/pics/FARG/INIDEP-DI_0463%2B1196178800.JPG	2025-06-15
Psammobatis_lentiginosa	http://www.fishbase.se/images/species/Pslen_u0.jpg	2025-06-15
Psammobatis_lentiginosa	http://www.fishbase.se/images/species/Pslen_u1.jpg	2025-06-15
Psammobatis_normani	http://www.boldsystems.org/pics/CCB/RM-001%2B1355343512.JPG	2025-06-15
Psammobatis_normani	http://www.boldsystems.org/pics/CCB/RM-003%2B1355343516.JPG	2025-06-15
Psammobatis_normani	http://www.boldsystems.org/pics/FARG/INIDEP-DI_0377%2B1193843264.jpg	2025-06-15
Psammobatis_normani	http://www.boldsystems.org/pics/FARG/INIDEP-DI_0378%2B1193843718.jpg	2025-06-15
Psammobatis_normani	http://www.boldsystems.org/pics/FARG/INIDEP-DI_0389%2B1193851602.jpg	2025-06-15
Psammobatis_normani	http://www.boldsystems.org/pics/FARG/INIDEP-DI_0391%2B1193853232.jpg	2025-06-15
Psammobatis_normani	http://www.boldsystems.org/pics/FARG/INIDEP-T_0073%2B1137513162.JPG	2025-06-15
Psammobatis_normani	http://www.boldsystems.org/pics/FARG/INIDEP-T_0074%2B1137754776.JPG	2025-06-15
Psammobatis_normani	http://www.boldsystems.org/pics/FARG/INIDEP-T_0076%2B1138018210.JPG	2025-06-15
Psammobatis_normani	http://www.boldsystems.org/pics/FARGB/CEGAR_DI_038%2B1305242390.jpg	2025-06-15
Psammobatis_normani	http://www.fishbase.se/images/species/Psnor_m0.jpg	2025-06-15
Psammobatis_normani	http://www.fishbase.se/images/species/Psnor_m1.jpg	2025-06-15
Psammobatis_rudis	http://shark-references.com/images/species/IMG_8872.jpg	2025-06-15
Psammobatis_rudis	http://www.boldsystems.org/pics/CCB/RM-013%2B1355343534.JPG	2025-06-15
Psammobatis_rudis	http://www.boldsystems.org/pics/FARG/INIDEP-DI_0390%2B1193852840.jpg	2025-06-15
Psammobatis_rudis	http://www.boldsystems.org/pics/FARG/INIDEP-DI_0468%2B1196165826.JPG	2025-06-15
Psammobatis_rudis	http://www.boldsystems.org/pics/FARG/INIDEP-DI_0478%2B1196166298.JPG	2025-06-15
Psammobatis_rudis	http://www.boldsystems.org/pics/FARG/INIDEP-DI_0493%2B1196271676.JPG	2025-06-15
Psammobatis_rudis	http://www.boldsystems.org/pics/FARG/INIDEP-T_0070%2B1137285426.JPG	2025-06-15
Psammobatis_rudis	http://www.boldsystems.org/pics/FARG/INIDEP-T_0075%2B1137857842.JPG	2025-06-15
Psammobatis_rudis	http://www.boldsystems.org/pics/FARG/INIDEP-T_0077%2B1138293194.JPG	2025-06-15
Psammobatis_rudis	http://www.boldsystems.org/pics/FARG/INIDEP-T_0122%2B1139856766.JPG	2025-06-15
Psammobatis_rudis	http://www.boldsystems.org/pics/FARG/INIDEP-T_0123%2B1139856874.JPG	2025-06-15
Psammobatis_rudis	http://www.fishbase.se/images/species/Psrud_f0.jpg	2025-06-15
Psammobatis_rudis	http://www.fishbase.se/images/species/Psrud_m0.jpg	2025-06-15
Psammobatis_rutrum	http://www.boldsystems.org/pics/RAJBR/movi2148-d%2B1226821550.jpg	2025-06-15
Psammobatis_rutrum	http://www.boldsystems.org/pics/RAJBR/movi2368-d%2B1226889022.jpg	2025-06-15
Psammobatis_rutrum	http://www.boldsystems.org/pics/RAJBR/movi2384-d%2B1226888860.jpg	2025-06-15

Psammobatis_rutrum	http://www.fishbase.se/images/species/Psrut_u1.jpg	2025-06-15
Psammobatis_rutrum	http://www.fishbase.se/images/species/Psrut_u2.jpg	2025-06-15
Psammobatis_scobina	http://shark-references.com/images/species/DSC00272.jpg	2025-06-15
Psammobatis_scobina	http://shark-references.com/images/species/Psammobatis%20scobina%20female.jpg	2025-06-15
Psammobatis_scobina	http://shark-references.com/images/species/Psammobatis%20scobina%20male.jpg	2025-06-15
Psammobatis_scobina	http://www.fishbase.se/images/species/Pssco_m0.jpg	2025-06-15
Psammobatis_scobina	http://www.fishbase.se/images/species/Pssco_u0.jpg	2025-06-15
Pseudobatos_lentiginosus	http://www.boldsystems.org/pics/MXIV/MXIV586%2B1277256354.JPG	2025-06-15
Pseudobatos_lentiginosus	http://www.boldsystems.org/pics/MXV/MXV260%2B1299684740.JPG	2025-06-15
Pseudobatos_lentiginosus	http://www.fishbase.org/images/species/Rhlen_u2.jpg	2025-06-15
Pseudobatos_glaucostigmus	http://www.fishbase.se/photos/workimagethumb.php?s=http://www.fishbase.se/tools/UploadPhoto/uploads/1378270852_182.52.138.153.jpg&w=600	2025-06-15
Pseudobatos_glaucostigmus	http://www.fishbase.se/photos/workimagethumb.php?s=http://www.fishbase.se/tools/UploadPhoto/uploads/1378365390_182.52.138.68.jpg&w=600	2025-06-15
Pseudobatos_glaucostigmus	http://www.fishbase.se/tools/UploadPhoto/uploads/Rhinobatosglaucostigma.jpg	2025-06-15
Pseudobatos_horkelii	http://www.fishbase.se/images/species/Rhhor_j0.jpg	2025-06-15
Pseudobatos_horkelii	http://www.fishbase.se/images/species/Rhhor_j1.jpg	2025-06-15
Pseudobatos_horkelii	http://www.fishbase.se/images/species/Rhhor_u0.jpg	2025-06-15
Pseudobatos_horkelii	http://www.fishbase.se/images/species/Rhhor_u1.jpg	2025-06-15
Pseudobatos_percellens	http://shark-references.com/images/species/Rhinobatos_leucorhynchus.jpg	2025-06-15
Pseudobatos_leucorhynchus	http://www.fishbase.org/images/species/Rhleu_u0.jpg	2025-06-15
Pseudobatos_leucorhynchus	http://www.fishbase.org/images/species/Rhleu_u4.jpg	2025-06-15
Pseudobatos_percellens	http://www.fishbase.org/images/species/Rhper_f1.jpg	2025-06-15
Pseudobatos_percellens	http://www.fishbase.org/images/species/Rhper_u0.jpg	2025-06-15
Pseudobatos_percellens	http://www.fishbase.org/images/species/Rhper_u1.jpg	2025-06-15
Pseudobatos_percellens	http://www.fishbase.org/images/species/Rhper_u3.jpg	2025-06-15
Pseudobatos_percellens	http://www.fishbase.org/images/species/Rhper_u4.jpg	2025-06-15
Pseudobatos_planiceps	http://shark-references.com/images/species/Rhinobatos%20planiceps.jpg	2025-06-15
Pseudobatos_planiceps	http://www.fishbase.org/images/species/Rhpla_u0.jpg	2025-06-15
Pseudobatos_prahli	http://shark-references.com/images/species/Rhinobatos_prahli.jpg	2025-06-15
Pseudobatos_prahli	http://www.discoverlife.org/IM/I_RR/0037/640/Rhinobatos_prahli,I_RR3735.jpg	2025-06-15
Pseudobatos_prahli	http://www.fishbase.org/images/species/Rhpra_u0.jpg	2025-06-15
Pseudobatos_productus	http://www.fishbase.org/images/species/Rhpro_u0.jpg	2025-06-15
Pseudobatos_productus	http://www.fishbase.org/images/species/Rhpro_u1.jpg	2025-06-15
Pseudobatos_productus	http://www.fishbase.org/images/species/Rhpro_u2.jpg	2025-06-15
Pteroplatytrygon_violacea	http://fishesofaustralia.net.au/images/image/PteroplatytrygonViolaceaCSIRO.jpg	2025-06-15
Pteroplatytrygon_violacea	http://shark-references.com/images/species/Dasyatis_violacea.jpg	2025-06-15
Pteroplatytrygon_violacea	http://shark-references.com/images/species/Pteroplatytrygon_violacea.jpg	2025-06-15
Pteroplatytrygon_violacea	http://www.boldsystems.org/pics/BIM/E42.6%2B1371745214.JPG	2025-06-15
Pteroplatytrygon_violacea	http://www.boldsystems.org/pics/EFBD/BPS1814A%2B1307024844.JPG	2025-06-15
Pteroplatytrygon_violacea	http://www.boldsystems.org/pics/ELAME/003149d%2B1318363192.JPG	2025-06-15
Pteroplatytrygon_violacea	http://www.boldsystems.org/pics/ELAME/003150d1%2B1318363192.JPG	2025-06-15
Pteroplatytrygon_violacea	http://www.boldsystems.org/pics/ELAME/003150v%2B1318363192.JPG	2025-06-15
Pteroplatytrygon_violacea	http://www.fishbase.se/images/species/Ptvio_f0.jpg	2025-06-15
Pteroplatytrygon_violacea	http://www.fishbase.se/images/species/Ptvio_f2.jpg	2025-06-15

Pteroplatytrygon_violacea	http://www.fishbase.se/images/species/Ptvio_m0.jpg	2025-06-15
Pteroplatytrygon_violacea	http://www.fishbase.se/images/species/Ptvio_u0.jpg	2025-06-15
Raja_asterias	http://shark-references.com/images/species/Raja_asterias_2.jpg	2025-06-15
Raja_asterias	http://shark-references.com/images/species/Raja_asterias_3.jpg	2025-06-15
Raja_asterias	http://www.fishbase.se/images/species/Raast_u0.jpg	2025-06-15
Raja_asterias	http://www.fishbase.se/photos/workimagethumb.php?s=http://www.fishbase.se/tools/UploadPhoto/uploads/1391205942_78.181.254.237.jpg&w=600	2025-06-15
Raja_asterias	http://www.fishbase.se/tools/UploadPhoto/uploads/1391206218_79.55.206.251.jpg	2025-06-15
Raja_asterias	http://www.fishbase.se/tools/UploadPhoto/uploads/P3230010.JPG	2025-06-15
Raja_brachyura	http://www.fishbase.se/images/species/Rabra_u2.jpg	2025-06-15
Raja_brachyura	http://www.fishbase.se/images/species/Rabra_u4.jpg	2025-06-15
Raja_brachyura	http://www.fishbase.se/photos/workimagethumb.php?s=http://www.fishbase.se/tools/UploadPhoto/uploads/1448878224_151.61.115.88.jpg&w=600	2025-06-15
Raja_brachyura	http://www.fishbase.se/tools/UploadPhoto/uploads/Raja_brachyura_Sardegna09_5561_.JPG	2025-06-15
Raja_brachyura	http://www.fishbase.se/tools/UploadPhoto/uploads/Raja_brachyura_Sardegna09_5575_.JPG	2025-06-15
Raja_brachyura	http://www.fishbase.se/tools/UploadPhoto/uploads/RBRA-ce071113.dr.JPG	2025-06-15
Raja_clavata	http://shark-references.com/images/species/Raja_brachyura.jpg	2025-06-15
Raja_clavata	http://shark-references.com/images/species/Raja_clavata_Coll.jpg	2025-06-15
Raja_clavata	http://www.fishbase.se/images/species/Amrad_u2.jpg	2025-06-15
Raja_clavata	http://www.fishbase.se/images/species/Racla_m0.jpg	2025-06-15
Raja_clavata	http://www.fishbase.se/images/species/Racla_u1.jpg	2025-06-15
Raja_clavata	http://www.fishbase.se/images/species/Racla_u3.jpg	2025-06-15
Raja_clavata	http://www.fishbase.se/images/species/Racla_u4.jpg	2025-06-15
Raja_clavata	http://www.fishbase.se/images/species/Racla_u6.jpg	2025-06-15
Raja_clavata	http://www.fishbase.se/photos/workimagethumb.php?s=http://www.fishbase.se/tools/UploadPhoto/uploads/1419177563_82.156.224.224.jpg&w=600	2025-06-15
Raja_clavata	http://www.fishbase.se/tools/UploadPhoto/uploads/1360347910_41.241.124.236.jpg	2025-06-15
Raja_clavata	http://www.fishbase.se/tools/UploadPhoto/uploads/Raja_clavata_Genova_7130_94.jpg	2025-06-15
Raja_clavata	http://www.fishbase.se/tools/UploadPhoto/uploads/Raja_clavata_Genova_7131_93.jpg	2025-06-15
Raja_clavata	http://www.fishbase.se/tools/UploadPhoto/uploads/Raja_clavata_Genova_7136._94jpg.jpg	2025-06-15
Raja_clavata	http://www.fishbase.se/tools/UploadPhoto/uploads/RAJCLA.JPG	2025-06-15
Raja_herwigi	http://www.fishbase.se/images/species/Raher_m0.jpg	2025-06-15
Raja_maderensis	http://www.fishbase.se/images/species/Ramad_u0.jpg	2025-06-15
Raja_microocellata	http://www.fishbase.se/images/species/Ramic_u0.jpg	2025-06-15
Raja_microocellata	http://www.fishbase.se/photos/workimagethumb.php?s=http://www.fishbase.se/tools/UploadPhoto/uploads/1381241742_217.120.237.5.jpg&w=600	2025-06-15
Raja_miraletus	http://shark-references.com/images/species/Raja_miraletus_3_Valencia.jpg	2025-06-15
Raja_miraletus	http://shark-references.com/images/species/raja_miraletus_valencia.jpg	2025-06-15
Raja_miraletus	http://www.boldsystems.org/pics/BIM/S64.6%2B978325200.JPG	2025-06-15
Raja_miraletus	http://www.boldsystems.org/pics/ELAME/2M08104RMI%2B1260411088.jpg	2025-06-15
Raja_miraletus	http://www.boldsystems.org/pics/ELAME/CNRAMCMazara037%2B1213820926.JPG	2025-06-15
Raja_miraletus	http://www.boldsystems.org/pics/ELAME/d000021%2B1260411112.JPG	2025-06-15
Raja_miraletus	http://www.boldsystems.org/pics/ELAME/d000732%2B1260411078.JPG	2025-06-15
Raja_miraletus	http://www.boldsystems.org/pics/ELAME/d001505%2B1260411110.JPG	2025-06-15
Raja_miraletus	http://www.boldsystems.org/pics/ELAME/IMG_2430%2B1260411096.JPG	2025-06-15
Raja_miraletus	http://www.boldsystems.org/pics/FCFP/FCFOP64-44%2B1041404400.JPG	2025-06-15
Raja_miraletus	http://www.boldsystems.org/pics/JFS/RaMi47S%2B1406126244.jpg	2025-06-15

Raja_miraletus	http://www.fishbase.se/images/species/Ramir_u0.jpg	2025-06-15
Raja_miraletus	http://www.fishbase.se/images/species/Ramir_u1.jpg	2025-06-15
Raja_miraletus	http://www.fishbase.se/images/species/Ramir_u2.jpg	2025-06-15
Raja_miraletus	http://www.fishbase.se/photos/workimagethumb.php?s=http://www.fishbase.se/tools/UploadPhoto/uploads/1388922803_223.185.27.171.jpg&w=600	2025-06-15
Raja_miraletus	http://www.fishbase.se/photos/workimagethumb.php?s=http://www.fishbase.se/tools/UploadPhoto/uploads/1388922926_223.185.27.171.jpg&w=600	2025-06-15
Raja_miraletus	http://www.fishbase.se/photos/workimagethumb.php?s=http://www.fishbase.se/tools/UploadPhoto/uploads/1471899648_188.114.102.50.jpg&w=600	2025-06-15
Raja_miraletus	http://www.fishbase.se/tools/UploadPhoto/uploads/RAJMIR.JPG	2025-06-15
Raja_montagui	http://www.boldsystems.org/pics/BNSFI/MT04182%2B1375216186.jpg	2025-06-15
Raja_montagui	http://www.boldsystems.org/pics/BNSFI/MT04184%2B1375216190.jpg	2025-06-15
Raja_montagui	http://www.boldsystems.org/pics/NBMF/Raja_montag_ZMUB_20819%2B1481124228.jpg	2025-06-15
Raja_montagui	http://www.boldsystems.org/pics/RNEZ/RNEZ227D%2B1265391580.jpg	2025-06-15
Raja_montagui	http://www.fishbase.se/images/species/Ramon_u0.jpg	2025-06-15
Raja_montagui	http://www.fishbase.se/images/species/Ramon_u1.jpg	2025-06-15
Raja_montagui	http://www.fishbase.se/images/species/Ramon_u2.jpg	2025-06-15
Raja_montagui	http://www.fishbase.se/tools/UploadPhoto/uploads/spottedray.jpg	2025-06-15
Raja_montagui	https://farm9.staticflickr.com/8159/7226932430_562a4a058e.jpg	2025-06-15
Raja_polystigma	http://www.boldsystems.org/pics/ELAME/2M08107RPO%2B1260411078.jpg	2025-06-15
Raja_polystigma	http://www.boldsystems.org/pics/ELAME/3M08107RPO%2B1260411074.jpg	2025-06-15
Raja_polystigma	http://www.boldsystems.org/pics/ELAME/4M08107RPO%2B1260411078.jpg	2025-06-15
Raja_polystigma	http://www.boldsystems.org/pics/ELAME/CNRAMCMazara075%2B1213891758.JPG	2025-06-15
Raja_polystigma	http://www.boldsystems.org/pics/ELAME/CNRAMCMazara076%2B1213891768.JPG	2025-06-15
Raja_polystigma	http://www.boldsystems.org/pics/ELAME/d000238%2B1260411084.JPG	2025-06-15
Raja_polystigma	http://www.boldsystems.org/pics/ELAME/d000240%2B1260411084.JPG	2025-06-15
Raja_polystigma	http://www.boldsystems.org/pics/ELAMO/EL097%2B1277978804.jpg	2025-06-15
Raja_polystigma	http://www.fishbase.se/images/species/Rapol_u0.jpg	2025-06-15
Raja_polystigma	http://www.fishbase.se/photos/workimagethumb.php?s=http://www.fishbase.se/tools/UploadPhoto/uploads/1448878384_151.61.115.88.jpg&w=600	2025-06-15
Raja_radula	http://artedi.nrm.se/nrmfish/images/tNRM64393_209.jpg	2025-06-15
Raja_radula	http://www.boldsystems.org/pics/ELAME/d000703%2B1260411072.JPG	2025-06-15
Raja_radula	http://www.boldsystems.org/pics/ELAME/IMG_1951%2B1260411082.JPG	2025-06-15
Raja_radula	http://www.boldsystems.org/pics/ELAME/IMG_1955%2B1260411080.JPG	2025-06-15
Raja_radula	http://www.boldsystems.org/pics/ELAMO/EL088%2B1277977848.jpg	2025-06-15
Raja_radula	http://www.boldsystems.org/pics/ELAMO/EL090%2B1277977942.jpg	2025-06-15
Raja_radula	http://www.fishbase.se/images/species/Rarad_u0.jpg	2025-06-15
Raja_radula	http://www.fishbase.se/tools/UploadPhoto/uploads/Raja_radula_Koufonissi08_a_3131_.JPG	2025-06-15
Raja_radula	http://www.fishbase.se/tools/UploadPhoto/uploads/Raja_radula_Paros2010_a_2380_.JPG	2025-06-15
Raja_radula	http://www.fishbase.se/tools/UploadPhoto/uploads/Raja_radula_Paros2010_b_2392_.JPG	2025-06-15
Raja_radula	http://www.fishbase.se/tools/UploadPhoto/uploads/Raja_radula_Paros2010_c_2897_.JPG	2025-06-15
Raja_radula	http://www.fishbase.se/tools/UploadPhoto/uploads/Razza_scuffina_Raja_radula_Koufonissi08_3166_.JPG	2025-06-15
Raja_straeleni	http://www.boldsystems.org/pics/ELAME/d001624%2B1260411106.JPG	2025-06-15
Raja_straeleni	http://www.boldsystems.org/pics/ELAME/d001636%2B1260411108.JPG	2025-06-15
Raja_straeleni	http://www.boldsystems.org/pics/ELAME/d001655%2B1260411108.JPG	2025-06-15
Raja_straeleni	http://www.boldsystems.org/pics/ELAME/d001660%2B1260411074.JPG	2025-06-15
Raja_straeleni	http://www.fishbase.se/images/species/Rastr_j0.jpg	2025-06-15

Raja_straeleni	http://www.fishbase.se/images/species/Rastr_u0.jpg	2025-06-15
Raja_straeleni	http://www.fishbase.se/images/species/Rastr_u1.jpg	2025-06-15
Raja_straeleni	http://www.fishbase.se/photos/workimagethumb.php?s=http://www.fishbase.se/tools/UploadPhoto/uploads/1397973888_197.87.220.53.jpg&w=600	2025-06-15
Raja_undulata	http://www.boldsystems.org/pics/ELAMO/2899-D%2B1269556510.JPG	2025-06-15
Raja_undulata	http://www.boldsystems.org/pics/ELAMO/EL110%2B1277991910.jpg	2025-06-15
Raja_undulata	http://www.fishbase.se/images/species/Raund_u0.jpg	2025-06-15
Raja_undulata	http://www.fishbase.se/images/species/Raund_u1.jpg	2025-06-15
Raja_undulata	http://www.fishbase.se/images/species/Raund_u2.jpg	2025-06-15
Raja_undulata	http://www.fishbase.se/tools/UploadPhoto/uploads/1351850069_2.9.192.149.jpg	2025-06-15
Raja_undulata	http://www.fishbase.se/tools/UploadPhoto/uploads/Raja_undulata_Genova_7132_93.jpg	2025-06-15
Raja_undulata	http://www.fishbase.se/tools/UploadPhoto/uploads/rajaundulata.jpg	2025-06-15
Raja_undulata	https://upload.wikimedia.org/wikipedia/commons/thumb/4/4a/Raja_undulata_2.jpg/500px-Raja_undulata_2.jpg	2025-06-15
Rajella_barnardi	http://www.boldsystems.org/pics/ELAME/d001486%2B1260411100.JPG	2025-06-15
Rajella_barnardi	http://www.boldsystems.org/pics/ELAME/d001487%2B1260411098.JPG	2025-06-15
Rajella_barnardi	http://www.boldsystems.org/pics/ELAME/d001492%2B1260411080.JPG	2025-06-15
Rajella_barnardi	http://www.boldsystems.org/pics/HVDB/IMG_1332%2B324668060.JPG	2025-06-15
Rajella_bathyphila	http://www.boldsystems.org/pics/SCAFB/07-360%2B1186676864.jpg	2025-06-15
Rajella_bathyphila	http://www.boldsystems.org/pics/SCAFB/07-393%2B1187112326.jpg	2025-06-15
Rajella_bathyphila	http://www.boldsystems.org/pics/SCAFB/07-400%2B1187112322.jpg	2025-06-15
Rajella_bathyphila	http://www.boldsystems.org/pics/SCFAC/OBS373_Abyssal_F_juvenile_%5B78cm%5D%2B1159967660.JPG	2025-06-15
Rajella_bathyphila	http://www.boldsystems.org/pics/SCFAC/OBS378_Abyssal_M_running_%5B89cm%5D%2B1160053984.JPG	2025-06-15
Rajella_bathyphila	http://www.fishbase.se/images/species/Rabat_u0.jpg	2025-06-15
Rajella_bigelowi	https://v3.boldsystems.org/pics/KUBIV/158963%2B1327953546.jpg	2025-06-15
Rajella_challengeri	http://shark-references.com/images/species/Rajella_challengeri_first.jpg	2025-06-15
Rajella_challengeri	http://shark-references.com/images/species/Rajella_challengeri_main.jpg	2025-06-15
Rajella_challengeri	http://shark-references.com/images/species/Rajella_challengeri_paratype.jpg	2025-06-15
Rajella_dissimilis	http://www.boldsystems.org/pics/DSSAU/Rajella_dissimilis_dorsal_1%2B1191934202.JPG	2025-06-15
Rajella_dissimilis	http://www.boldsystems.org/pics/ELAME/d001637%2B1260411100.JPG	2025-06-15
Rajella_dissimilis	http://www.boldsystems.org/pics/ELAME/d001638%2B1260411074.JPG	2025-06-15
Rajella_dissimilis	http://www.boldsystems.org/pics/ELAME/d001639%2B1260411112.JPG	2025-06-15
Rajella_fyllae	http://artedi.nrm.se/nrmfish/images/tNRM60470b.jpg	2025-06-15
Rajella_fyllae	http://dsiphoto.mnhn.fr/gicim/sd00031/i2003-0538_p1.jpg	2025-06-15
Rajella_fyllae	http://www.boldsystems.org/pics/ELAME/d000825%2B1260411094.JPG	2025-06-15
Rajella_fyllae	http://www.boldsystems.org/pics/ELAME/d000831%2B1260411078.JPG	2025-06-15
Rajella_fyllae	http://www.boldsystems.org/pics/ELAME/d000833%2B1260411102.JPG	2025-06-15
Rajella_fyllae	http://www.boldsystems.org/pics/RNEZ/RNEZ202D%2B1265392282.jpg	2025-06-15
Rajella_fyllae	http://www.boldsystems.org/pics/RNEZ/RNEZ205D%2B1265392836.jpg	2025-06-15
Rajella_fyllae	http://www.boldsystems.org/pics/RNEZ/RNEZ207D%2B1265396832.jpg	2025-06-15
Rajella_fyllae	http://www.boldsystems.org/pics/RNEZ/RNEZ208D%2B1265397458.jpg	2025-06-15
Rajella_fyllae	http://www.boldsystems.org/pics/RNEZ/RNEZ209D%2B1265397804.jpg	2025-06-15
Rajella_fyllae	http://www.boldsystems.org/pics/RNEZ/RNEZ261D%2B1265399370.jpg	2025-06-15
Rajella_fyllae	http://www.fishbase.se/images/species/Rafyl_u0.jpg	2025-06-15
Rajella_kukujevi	http://www.fishbase.se/images/species/Rakuk_u0.jpg	2025-06-15

Rajella_kukujevi	http://www.fishbase.se/images/species/Rakuk_u2.jpg	2025-06-15
Rajella_leoparda	http://www.boldsystems.org/pics/ELAME/d001481%2B1260411070.JPG	2025-06-15
Rajella_leoparda	http://www.boldsystems.org/pics/ELAME/d001482%2B1260411112.JPG	2025-06-15
Rajella_leoparda	http://www.boldsystems.org/pics/ELAME/d001484%2B1260411078.JPG	2025-06-15
Rajella_leoparda	http://www.boldsystems.org/pics/ELAME/d001506%2B1260411072.JPG	2025-06-15
Rajella_leoparda	http://www.fishbase.se/images/species/Raleo_u0.jpg	2025-06-15
Rajella_lintea	http://www.fishbase.se/images/species/Dilin_u2.jpg	2025-06-15
Rajella_nigerrima	http://www.fishbase.se/images/species/Ranig_f0.jpg	2025-06-15
Rajella_purpuriventralis	http://www.fishbase.se/images/species/Rapur_m0.jpg	2025-06-15
Rajella_sadowskii	http://www.fishbase.se/images/species/Rasad_f0.jpg	2025-06-15
Rajella_sadowskii	https://www.researchgate.net/profile/Paulo_Costa23/publication/231754716/figure/fig3/AS:300376852058115@1448626830745/Fig-3-Fresh-specimen-of-Rajella-sadowskii-MOVI-36984-female-330-mm-DW-558-mm-TL.png	2025-06-15
Rajella_sadowskii	https://www.researchgate.net/profile/Paulo_Costa23/publication/231754716/figure/fig9/AS:300376856252420@1448626831359/Fig-9-Egg-capsule-with-fresh-advanced-embryo-of-Rajella-sadowskii-MOVI-36991-female.png	2025-06-15
Rhina_ancylostoma	http://68.media.tumblr.com/tumblr_lvvas4GhYS1qitkrlo1_1280.jpg	2025-06-15
Rhina_ancylostoma	http://fishesofaustralia.net.au/Images/Image/Rhinaancylostoma.jpg	2025-06-15
Rhina_ancylostoma	http://fishesofaustralia.net.au/images/image/RhinaAncylostomaJinsukKim.jpg	2025-06-15
Rhina_ancylostoma	http://www.boldsystems.org/pics/IFV/MSWCF338%2B1490028738.JPG	2025-06-15
Rhina_ancylostoma	http://www.fishbase.org/images/species/Rhanc_u0.jpg	2025-06-15
Rhina_ancylostoma	http://www.fishbase.org/images/species/Rhanc_u1.jpg	2025-06-15
Rhina_ancylostoma	http://www.fishbase.org/images/species/Rhanc_u2.jpg	2025-06-15
Rhinobatos_albomaculatus	http://www.theonlinezoo.com/img/24/toz24638s.jpg	2025-06-15
Rhinobatos_annandalei	http://www.fishbase.org/images/species/Rhann_u0.jpg	2025-06-15
Rhinobatos_borneensis	http://shark-references.com/images/species/Rhinobatos_borneensis_first.jpg	2025-06-15
Rhinobatos_holcorhynchus	http://www.fishbase.org/images/species/Rhhol_u1.jpg	2025-06-15
Rhinobatos_hynnicephalus	http://www.fishbase.org/images/species/Rhhyn_u0.jpg	2025-06-15
Pseudobatos_productus	https://www.fishbase.se/images/species/Rhpro_u1.jpg	2025-06-15
Pseudobatos_productus	https://www.fishbase.se/images/species/Rhpro_u2.jpg	2025-06-15
Pseudobatos_productus	https://www.fishbase.se/images/species/Rhpro_u0.jpg	2025-06-15
Rhinobatos_punctifer	http://www.boldsystems.org/pics/IFV/KMF140%2B1490286146.JPG	2025-06-15
Rhinobatos_punctifer	http://www.boldsystems.org/pics/NNPF/1089-b%2B1261111698.jpg	2025-06-15
Rhinobatos_punctifer	http://www.fishbase.se/images/species/Rhpun_u0.jpg	2025-06-15
Rhinobatos_punctifer	http://www.fishbase.se/images/species/Rhpun_u1.jpg	2025-06-15
Rhinobatos_punctifer	http://www.fishbase.se/photos/workimagethumb.php?s=http://www.fishbase.se/tools/UploadPhoto/uploads/1383625474_182.52.68.117.jpg&w=600	2025-06-15
Rhinobatos_punctifer	http://www.fishbase.se/photos/workimagethumb.php?s=http://www.fishbase.se/tools/UploadPhoto/uploads/1476630197_162.158.30.12.jpg&w=600	2025-06-15
Rhinobatos_rhinobatos	http://www.boldsystems.org/pics/BIM/H038.1_Rhinobatos_rhinobatos%2B1372950774.JPG	2025-06-15
Rhinobatos_rhinobatos	http://www.boldsystems.org/pics/BIM/S_47.3_Rhinobatos_rhinobatos%2B1372950938.JPG	2025-06-15
Rhinobatos_rhinobatos	http://www.fishbase.se/images/species/Rhrhi_u0.jpg	2025-06-15
Rhinobatos_sainsburyi	http://fishesofaustralia.net.au/images/image/Rhinobatossainsburyi.jpg	2025-06-15
Rhinobatos_schlegelii	http://www.fishbase.se/images/species/Rhsch_u0.jpg	2025-06-15
Rhinobatos_schlegelii	http://www.fishbase.se/tools/UploadPhoto/uploads/R.schlegelii.jpg	2025-06-15
Rhinobatos_whitei	http://shark-references.com/images/species/Fig%201A%20079-fresh-dorsal.jpg	2025-06-15
Rhinoptera_bonasus	http://shark-references.com/images/species/Atlantic_Cownose_Ray_003.jpg	2025-06-15

Rhinoptera_bonasus	http://shark-references.com/images/species/Caribbean%20Cownose-Ray-002.jpg	2025-06-15
Rhinoptera_bonasus	http://shark-references.com/images/species/Rhinoptera_bonasus_Rangel.jpg	2025-06-15
Rhinoptera_bonasus	http://www.boldsystems.org/pics/FLBAR/FWRI_01339_0406%2B1479492866.JPG	2025-06-15
Rhinoptera_brasiliensis	http://www.fishbase.se/images/species/Rhbra_m0.jpg	2025-06-15
Rhinoptera_brasiliensis	http://www.fishbase.se/images/species/Rhbra_u0.jpg	2025-06-15
Rhinoptera_javanica	http://fishesofaustralia.net.au/images/image/RhinopteraJavanicaCSIRO.jpg	2025-06-15
Rhinoptera_javanica	http://shark-references.com/images/species/Rhinoptera_adspersa_1.jpg	2025-06-15
Rhinoptera_javanica	http://www.fishbase.se/images/species/Rhjav_u0.jpg	2025-06-15
Rhinoptera_javanica	http://www.fishbase.se/tools/UploadPhoto/uploads/Rhinopterajavanica.jpg	2025-06-15
Rhinoptera_jayakari	http://www.fishbase.se/images/species/Rhjay_u0.jpg	2025-06-15
Rhinoptera_jayakari	http://www.fishbase.se/tools/UploadPhoto/uploads/1366645593_182.178.10.206.jpg	2025-06-15
Rhinoptera_jayakari	http://www.fishbase.se/tools/UploadPhoto/uploads/1366645618_182.178.10.206.jpg	2025-06-15
Rhinoptera_marginata	http://www.fishbase.se/photos/workimagethumb.php?s=http://www.fishbase.se/tools/UploadPhoto/uploads/1383161472_85.64.220.252.jpg&w=600	2025-06-15
Rhinoptera_neglecta	http://fishesofaustralia.net.au/Images/Image/RhinopteraNeglecta2KG.jpg	2025-06-15
Rhinoptera_neglecta	http://www.fishbase.se/images/species/Rhneg_u0.jpg	2025-06-15
Rhinoptera_steindachneri	http://www.fishbase.se/images/species/Rhste_u0.jpg	2025-06-15
Rhinoptera_steindachneri	https://static.inaturalist.org/photos/2368521/medium.jpg?1441979202	2025-06-15
Rhynchobatus_australiae	https://fishesofaustralia.net.au/images/image/RhynchobatAustralDavidR.jpg	2025-06-15
Rhynchobatus_australiae	http://fishesofaustralia.net.au/images/image/RhynchobAstralCSIRO.jpg	2025-06-15
Rhynchobatus_australiae	http://fishesofaustralia.net.au/images/image/RhynchobatAust3GuySkillen.jpg	2025-06-15
Rhynchobatus_australiae	https://shark-references.com/images/species/5503_Rhdji_u2.jpg	2025-06-15
Rhynchobatus_australiae	https://shark-references.com/images/species/5282.jpg	2025-06-15
Rhynchobatus_australiae	http://www.boldsystems.org/pics/BASGA/BKKP23-H%2B1306974482.jpg	2025-06-15
Rhynchobatus_australiae	http://www.fishbase.org/images/species/Rhdji_u1.jpg	2025-06-15
Rhynchobatus_australiae	http://www.fishbase.org/images/species/Rhdji_u3.jpg	2025-06-15
Rhynchobatus_australiae	http://www.fishbase.org/tools/UploadPhoto/uploads/1367340680_182.178.50.77.jpg	2025-06-15
Rhynchobatus_australiae	https://1.bp.blogspot.com/-Vuihf36rV-8/V49Ikuw98tI/AAAAAAAA4zM/nsDMIrLRXoIw2_i0hSWNklJz4Z2pBzhSQCK4B/s400/Rhynchobatus_australiae-colour_variation-2016-Genetic_Phenotypi-Diversity_Giles-Riginos-Naylor-Dharmadi-et-Ovenden.png	2025-06-15
Rhynchobatus_compagnoi	http://shark-references.com/images/species/Rhynchobatus_cooki_holo.jpg	2025-06-15
Rhynchobatus_compagnoi	https://1.bp.blogspot.com/-Ub8s9_X0sy4/V57B8wAUFJI/AAAAAAAA5pY/S1qObrnHcHwMD8_xYiUlk-EPHBBM3aG0wCLcB/s400/Rhynchobatus_cooki-novataxa_2016-%2BLast-Kyne-et-Compagno_.png	2025-06-15
Rhynchobatus_compagnoi	https://4.bp.blogspot.com/-uEymwiFQhPg/V57B9397WGI/AAAAAAAA5pc/3Na_FSfjb2Ma8xA5ReiwdrV0yRv6zSp3gCLcB/s400/Rhynchobatus_cooki-novataxa_2016-Last-Kyne-et-Compagno_i.jpg	2025-06-15
Rhynchobatus_djiddensis	http://www.fishbase.org/images/species/Rhdji_u5.jpg	2025-06-15
Rhynchobatus_djiddensis	http://www.fishbase.org/photos/workimagethumb.php?s=http://www.fishbase.org/tools/UploadPhoto/uploads/1478687362_173.245.48.76.jpg&w=600	2025-06-15
Rhynchobatus_immaculatus	https://pbs.twimg.com/media/Cf7n2eGWsAAx1pO.jpg	2025-06-15
Rhynchobatus_laevis	http://fishesofaustralia.net.au/Images/Image/RhynchobatLaevisDavidR.jpg	2025-06-15
Rhynchobatus_laevis	http://shark-references.com/images/species/Rhynchobatus_laevis%20_2%20(1).jpg	2025-06-15
Rhynchobatus_laevis	http://shark-references.com/images/species/thumbnail/Rhynchobatus_laevis%20_2%20(2).jpg	2025-06-15
Rhynchobatus_laevis	http://www.boldsystems.org/pics/ANGEN/sample_SH%2B1420055248.jpg	2025-06-15
Rhynchobatus_laevis	http://www.fishbase.org/photos/workimagethumb.php?s=http://www.fishbase.org/tools/UploadPhoto/uploads/1383815845_118.175.90.164.jpg&w=600	2025-06-15
Rhynchobatus_laevis	http://www.fishbase.org/photos/workimagethumb.php?s=http://www.fishbase.org/tools/UploadPhoto/uploads/1383815998_118.175.90.164.jpg&w=600	2025-06-15
Rhynchobatus_laevis	http://www.fishbase.org/tools/UploadPhoto/uploads/1383816120_118.175.90.164.jpg	2025-06-15
Rhynchobatus_palpebratus	http://fishesofaustralia.net.au/images/image/Rhynchobatuspalpebratus.jpg	2025-06-15

Rhynchobatus_palpebratus	http://www.fishbase.se/images/species/Rhlae_u0.jpg	2025-06-15
Rhynchobatus_springeri	https://fishbase.se/tools/display_image.php?fw=n&imgName=1517456109_172.68.106.30.jpg	2025-06-15
Rhynchobatus_springeri	http://www.fishbase.se/images/species/Rhspr_u0.jpg	2025-06-15
Rhynchorhina_mauritaniensis	http://shark-references.com/images/species/thumbnail/Rhynchorhina_mauritaniensis_holo.jpg	2025-06-15
Rioraja_agassizi	http://shark-references.com/images/species/Riaga_m0.jpg	2025-06-15
Rioraja_agassizi	http://www.fishbase.se/images/species/Atpla_u0.jpg	2025-06-15
Rioraja_agassizi	http://www.fishbase.se/images/species/Riaga_j0.jpg	2025-06-15
Rioraja_agassizi	http://www.fishbase.se/images/species/Riaga_m0.jpg	2025-06-15
Rioraja_agassizi	http://www.fishbase.se/images/species/Riaga_u0.jpg	2025-06-15
Rioraja_agassizi	http://www.fishbase.se/photos/workimagethumb.php?s=http://www.fishbase.se/tools/UploadPhoto/uploads/1346244178_200.128.60.77.jpg&w=600	2025-06-15
Rostroraja_alba	http://www.boldsystems.org/pics/ELAME/1PC10RAL%2B1318363190.JPG	2025-06-15
Rostroraja_alba	http://www.boldsystems.org/pics/ELAME/d001534%2B1260411072.JPG	2025-06-15
Rostroraja_alba	http://www.boldsystems.org/pics/ELAME/d001596%2B1260411102.JPG	2025-06-15
Rostroraja_alba	http://www.boldsystems.org/pics/ELAME/d001597%2B1260411110.JPG	2025-06-15
Rostroraja_alba	http://www.boldsystems.org/pics/ELAME/d001662%2B1260411112.JPG	2025-06-15
Rostroraja_alba	http://www.boldsystems.org/pics/ELAME/d001672%2B1260411110.JPG	2025-06-15
Rostroraja_alba	http://www.fishbase.se/photos/workimagethumb.php?s=http://www.fishbase.se/tools/UploadPhoto/uploads/1393318816_80.239.24.2.jpg&w=600	2025-06-15
Rostroraja_alba	http://www.fishbase.se/tools/UploadPhoto/uploads/1359166168_217.120.237.5.jpg	2025-06-15
Rostroraja_alba	http://www.fishbase.se/tools/UploadPhoto/uploads/R_alba_dorsal.JPG	2025-06-15
Raja_eglanteria	http://shark-references.com/images/species/Raja%20eglanteria%20N08-A%20BEST.jpg	2025-06-15
Raja_eglanteria	http://www.fishbase.se/images/species/Raegl_u0.jpg	2025-06-15
Raja_equatorialis	http://www.fishbase.se/images/species/Raequ_u1.jpg	2025-06-15
Rostroraja_texana	http://shark-references.com/images/species/Raja-texana.jpg	2025-06-15
Rostroraja_texana	http://www.fishbase.se/images/species/Ratex_u1.jpg	2025-06-15
Rostroraja_velezi	http://www.fishbase.se/images/species/Ravel_u0.jpg	2025-06-15
Rostroraja_velezi	http://www.fishbase.se/images/species/Ravel_u3.jpg	2025-06-15
Sinobatis_borneensis	http://www.fishbase.se/photos/workimagethumb.php?s=http://www.fishbase.se/tools/UploadPhoto/uploads/MalaysianRay1.jpg&w=600	2025-06-15
Sinobatis_bulbicauda	http://fishesofaustralia.net.au/Images/Image/SinobatBulbicaud2CSIRO.jpg	2025-06-15
Sinobatis_bulbicauda	http://fishesofaustralia.net.au/images/image/SinobatisBulbicaudaCSIRO.jpg	2025-06-15
Sinobatis_caerulea	http://fishesofaustralia.net.au/images/image/SinobatisCaerulCSIRO.jpg	2025-06-15
Spiniraja_whitleyi	http://fishesofaustralia.net.au/Images/Image/DipturusWhitleyiRK.jpg	2025-06-15
Spiniraja_whitleyi	http://fishesofaustralia.net.au/Images/Image/SpinirajaWhitleyiCSIRO.jpg	2025-06-15
Spiniraja_whitleyi	http://fishesofaustralia.net.au/Images/Image/SpinirajaWhitleyiRLS.jpg	2025-06-15
Spiniraja_whitleyi	http://fishesofaustralia.net.au/images/image/SpinirajWhitleyiIanShaw.jpg	2025-06-15
Spiniraja_whitleyi	http://fishesofaustralia.net.au/Images/Image/SpinirajWhitMTreloar.jpg	2025-06-15
Spiniraja_whitleyi	http://fishesofaustralia.net.au/Images/Image/SpnirajaWhitleyiRK.jpg	2025-06-15
Styracura_pacifica	http://www.fishbase.se/images/species/Hipac_u0.jpg	2025-06-15
Styracura_pacifica	http://www.fishbase.se/images/species/Hipac_u1.jpg	2025-06-15
Styracura_schmardae	http://www.fishbase.se/tools/UploadPhoto/uploads/DSCI3009.JPG	2025-06-15
Styracura_schmardae	http://www.fishbase.se/tools/UploadPhoto/uploads/IMG_8337.JPG	2025-06-15
Sympterygia_acuta	http://shark-references.com/images/species/Sympterygia_acuta_argentinia.jpg	2025-06-15
Sympterygia_acuta	http://www.boldsystems.org/pics/CCB/MT6%2B1355343492.JPG	2025-06-15
Sympterygia_acuta	http://www.boldsystems.org/pics/CCB/SaM1%2B1355343476.JPG	2025-06-15

Sympterygia_acuta	http://www.boldsystems.org/pics/CCB/SaM2%2B1355343478.JPG	2025-06-15
Sympterygia_acuta	http://www.boldsystems.org/pics/FARG/INIDEP-DI_0405%2B1195142810.jpg	2025-06-15
Sympterygia_acuta	http://www.boldsystems.org/pics/FARG/INIDEP-DI_0407%2B1195144342.jpg	2025-06-15
Sympterygia_acuta	http://www.boldsystems.org/pics/FARG/INIDEP-DI_0771%2B1255876350.jpg	2025-06-15
Sympterygia_acuta	http://www.boldsystems.org/pics/FARG/INIDEP-DI_0775%2B1255875426.jpg	2025-06-15
Sympterygia_acuta	http://www.boldsystems.org/pics/FARGB/UNMDP_DI_1008%2B1305237044.jpg	2025-06-15
Sympterygia_acuta	http://www.fishbase.se/images/species/Syacu_f0.jpg	2025-06-15
Sympterygia_acuta	http://www.fishbase.se/images/species/Syacu_m0.jpg	2025-06-15
Sympterygia_acuta	http://www.fishbase.se/images/species/Syacu_u9.jpg	2025-06-15
Sympterygia_bonapartii	http://shark-references.com/images/species/bonapartii.jpg	2025-06-15
Sympterygia_bonapartii	http://www.boldsystems.org/pics/CCB/SbM2%2B1339779846.JPG	2025-06-15
Sympterygia_bonapartii	http://www.boldsystems.org/pics/FARG/INIDEP-T_0213%2B1137284588.JPG	2025-06-15
Sympterygia_bonapartii	http://www.boldsystems.org/pics/FARG/INIDEP-T_0214%2B1137284736.JPG	2025-06-15
Sympterygia_bonapartii	http://www.boldsystems.org/pics/FARG/INIDEP-T_0215%2B1137284856.JPG	2025-06-15
Sympterygia_bonapartii	http://www.fishbase.se/images/species/Sybon_f0.jpg	2025-06-15
Sympterygia_bonapartii	http://www.fishbase.se/images/species/Sybon_u1.jpg	2025-06-15
Sympterygia_brevicaudata	http://shark-references.com/images/species/IMG_0215.jpg	2025-06-15
Sympterygia_brevicaudata	http://www.discoverlife.org/IM/I_RR/0026/640/Sympterygia_brevicaudata,I_RR2626.jpg	2025-06-15
Sympterygia_brevicaudata	http://www.fishbase.se/images/species/Sybre_u3.jpg	2025-06-15
Sympterygia_lima	http://shark-references.com/images/species/IMG_0188.jpg	2025-06-15
Sympterygia_lima	http://www.fishbase.se/images/species/Sylim_u0.jpg	2025-06-15
Sympterygia_lima	http://www.fishbase.se/photos/workimagethumb.php?s=http://www.fishbase.se/tools/UploadPhoto/uploads/1425682784_201.214.53.53.jpg&w=600	2025-06-15
Taeniura_lessoni	http://shark-references.com/images/species/Taeniura_lessoni_main.jpg	2025-06-15
Taeniura_lymma	http://fishesofaustralia.net.au/images/image/TaeniuraLymmaWW.jpg	2025-06-15
Taeniura_lymma	http://shark-references.com/images/species/Taeniura_lymma_Treml_2.JPG	2025-06-15
Taeniura_lymma	http://shark-references.com/images/species/Taeniura_lymma_Treml.JPG	2025-06-15
Taeniura_lymma	http://www.boldsystems.org/pics/HVDBF/IMG_5445%2B1297202202.JPG	2025-06-15
Taeniura_lymma	http://www.boldsystems.org/pics/LIFS/UG012-1%2B1220507294.JPG	2025-06-15
Taeniura_lymma	http://www.boldsystems.org/pics/LIFS/UG0713_8845%2B1235579384.jpg	2025-06-15
Taeniura_lymma	http://www.boldsystems.org/pics/TZAIX/HLC-12409%2B1200345860.jpg	2025-06-15
Taeniura_lymma	http://www.boldsystems.org/pics/UKFBJ/T4903%2B1327953548.jpg	2025-06-15
Taeniura_lymma	http://www.fishbase.se/images/species/Talym_u0.jpg	2025-06-15
Taeniura_lymma	http://www.fishbase.se/images/species/Talym_u1.jpg	2025-06-15
Taeniura_lymma	http://www.fishbase.se/images/species/Talym_u3.jpg	2025-06-15
Taeniura_lymma	http://www.fishbase.se/images/species/Talym_u4.jpg	2025-06-15
Taeniura_lymma	http://www.fishbase.se/images/species/Talym_u6.jpg	2025-06-15
Taeniura_lymma	https://farm8.staticflickr.com/7034/6395392561_12ac744bc6.jpg	2025-06-15
Taeniurops_grabatus	http://shark-references.com/images/species/Taeniura_grabata_tenerife.jpg	2025-06-15
Taeniurops_grabatus	http://www.fishbase.se/images/species/Tagra_u1.jpg	2025-06-15
Taeniurops_grabatus	http://www.fishbase.se/images/species/Tagra_u2.jpg	2025-06-15
Taeniurops_grabatus	http://www.fishbase.se/images/species/Tagra_u3.jpg	2025-06-15
Taeniurops_grabatus	http://www.fishbase.se/images/species/Tagra_u4.jpg	2025-06-15
Taeniurops_meyeni	http://fishesofaustralia.net.au/images/image/TaenuirMeyeniNickHobgood.jpg	2025-06-15

Taeniurops_meyeni	http://shark-references.com/images/species/_SIN8466.jpg	2025-06-15
Taeniurops_meyeni	http://www.fishbase.se/images/species/Tamey_u0.jpg	2025-06-15
Taeniurops_meyeni	http://www.fishbase.se/images/species/Tamey_u1.jpg	2025-06-15
Taeniurops_meyeni	http://www.fishbase.se/images/species/Tamey_u4.jpg	2025-06-15
Taeniurops_meyeni	http://www.fishbase.se/images/species/Tamey_u5.jpg	2025-06-15
Telatrygon_acutirostra	http://www.fishbase.se/images/species/Daacu_u0.jpg	2025-06-15
Telatrygon_biasa	http://shark-references.com/images/species/Telatrygon_biasa_main.jpg	2025-06-15
Telatrygon_zugei	http://www.boldsystems.org/pics/JTFR/PDGN14%2B1497330476.jpg	2025-06-15
Telatrygon_zugei	http://www.boldsystems.org/pics/JTFR/PDGN15%2B1497330642.jpg	2025-06-15
Telatrygon_zugei	http://www.fishbase.se/images/species/Dazug_u0.jpg	2025-06-15
Telatrygon_zugei	http://www.fishbase.se/tools/UploadPhoto/uploads/1398782508_182.178.102.136.jpg	2025-06-15
Telatrygon_zugei	http://www.fishbase.se/tools/UploadPhoto/uploads/Dasyatis_zurgei1.jpg	2025-06-15
Temera_hardwickii	http://www.fishbase.se/tools/UploadPhoto/uploads/1359014096_180.180.127.98.jpg	2025-06-15
Temera_hardwickii	http://www.fishbase.se/tools/UploadPhoto/uploads/1450948646_42.60.236.198.jpg	2025-06-15
Tetronarce_californica	http://www.fishbase.se/images/species/Tocal_u0.jpg	2025-06-15
Tetronarce_californica	http://www.fishbase.se/tools/UploadPhoto/uploads/P8216472.JPG	2025-06-15
Tetronarce_californica	http://www.fishbase.se/tools/UploadPhoto/uploads/P8216473.JPG	2025-06-15
Tetronarce_californica	http://www.fishbase.se/tools/UploadPhoto/uploads/P8216527.JPG	2025-06-15
Tetronarce_formosa	http://shark-references.com/images/species/Taiwan%20Torpedo,%20Torpedo%20formosa,%20Haas%20&%20Ebert,%202006.jpg	2025-06-15
Tetronarce_nobiliana	http://www.fishbase.se/images/species/Tonob_u0.jpg	2025-06-15
Tetronarce_nobiliana	http://www.fishbase.se/images/species/Tonob_u1.jpg	2025-06-15
Tetronarce_nobiliana	http://www.fishbase.se/tools/UploadPhoto/uploads/TORNOB.JPG	2025-06-15
Tetronarce_nobiliana	http://www.fishbase.se/tools/UploadPhoto/uploads/weirdfish.jpg	2025-06-15
Tetronarce_puelcha	http://www.fishbase.se/tools/UploadPhoto/uploads/P1000833.jpg	2025-06-15
Tetronarce_tokionis	http://fishesofaustralia.net.au/images/image/TorpedoTokionisCSIRO.jpg	2025-06-15
Tetronarce_tokionis	http://www.fishbase.se/images/species/Totok_u0.jpg	2025-06-15
Tetronarce_tremens	http://www.fishbase.se/images/species/Totre_u0.jpg	2025-06-15
Tetronarce_tremens	http://www.fishbase.se/images/species/Totre_u1.jpg	2025-06-15
Tetronarce_tremens	http://www.fishbase.se/photos/workimagethumb.php?s=http://www.fishbase.se/tools/UploadPhoto/uploads/1343351155_202.123.56.189.jpg&w=600	2025-06-15
Tetronarce_tremens	http://www.fishbase.se/tools/UploadPhoto/uploads/torpedo_tremens.JPG	2025-06-15
Torpedo_fuscomaculata	http://www.boldsystems.org/pics/IFV/TOM12%2B1456435362.jpg	2025-06-15
Torpedo_fuscomaculata	http://www.boldsystems.org/pics/IFV/TOP14%2B1456435362.jpg	2025-06-15
Torpedo_fuscomaculata	http://www.boldsystems.org/pics/IFV/Topo22%2B1464310638.jpg	2025-06-15
Torpedo_fuscomaculata	http://www.boldsystems.org/pics/IFV/TOS13%2B1456435362.jpg	2025-06-15
Torpedo_fuscomaculata	http://www.boldsystems.org/pics/SAIAB/SAIAB_78295-T454%2B1147625536.JPG	2025-06-15
Torpedo_fuscomaculata	http://www.boldsystems.org/pics/SBF/NBE0540%2B1210417380.JPG	2025-06-15
Torpedo_fuscomaculata	http://www.fishbase.se/images/species/Tofus_u0.jpg	2025-06-15
Torpedo_marmorata	http://shark-references.com/images/species/Torpedo_marmorata_%20Barria.jpg	2025-06-15
Torpedo_marmorata	http://www.fishbase.se/images/species/Tomar_u0.jpg	2025-06-15
Torpedo_marmorata	http://www.fishbase.se/images/species/Tomar_u2.jpg	2025-06-15
Torpedo_marmorata	http://www.fishbase.se/images/species/Tomar_u3.jpg	2025-06-15
Torpedo_marmorata	http://www.fishbase.se/images/species/Tomar_u4.jpg	2025-06-15
Torpedo_marmorata	http://www.fishbase.se/tools/UploadPhoto/uploads/TorpedineMarezzata_Torpedo_marmorata_2499.JPG	2025-06-15

Torpedo_panthera	http://shark-references.com/images/species/Torpedo_panthera_Treml_1%20(2).JPG	2025-06-15
Torpedo_panthera	http://shark-references.com/images/species/Torpedo_panthera_Treml_1%20(3).JPG	2025-06-15
Torpedo_panthera	http://www.fishbase.se/images/species/Topan_u0.jpg	2025-06-15
Torpedo_panthera	http://www.fishbase.se/images/species/Topan_u1.jpg	2025-06-15
Torpedo_panthera	http://www.fishbase.se/images/species/Topan_u2.jpg	2025-06-15
Torpedo_panthera	http://www.fishbase.se/tools/UploadPhoto/uploads/029-1_P5216859_Torpedo_panthera.jpg	2025-06-15
Torpedo_panthera	http://www.fishbase.se/tools/UploadPhoto/uploads/1366112186_182.178.80.212.jpg	2025-06-15
Torpedo_panthera	http://www.fishbase.se/tools/UploadPhoto/uploads/1482256041_188.114.102.48.jpg	2025-06-15
Torpedo_panthera	http://www.fishbase.se/tools/UploadPhoto/uploads/FB_7822.jpg	2025-06-15
Torpedo_sinuspersici	http://shark-references.com/images/species/Torpedo_sinuspersici.jpg	2025-06-15
Torpedo_sinuspersici	http://www.fishbase.se/images/species/Tosin_u2.jpg	2025-06-15
Torpedo_sinuspersici	http://www.fishbase.se/images/species/Tosin_u4.jpg	2025-06-15
Torpedo_sinuspersici	http://www.fishbase.se/photos/workimagethumb.php?s=http://www.fishbase.se/tools/UploadPhoto/uploads/1384502456_182.178.43.75.jpg&w=600	2025-06-15
Torpedo_sinuspersici	http://www.fishbase.se/photos/workimagethumb.php?s=http://www.fishbase.se/tools/UploadPhoto/uploads/1405717695_154.122.122.222.jpg&w=600	2025-06-15
Torpedo_sinuspersici	http://www.fishbase.se/photos/workimagethumb.php?s=http://www.fishbase.se/tools/UploadPhoto/uploads/1406989729_27.114.165.3.jpg&w=600	2025-06-15
Torpedo_sinuspersici	http://www.fishbase.se/tools/UploadPhoto/uploads/1347121014_62.178.129.238.jpg	2025-06-15
Torpedo_sinuspersici	http://www.fishbase.se/tools/UploadPhoto/uploads/1366112661_182.178.80.212.jpg	2025-06-15
Torpedo_sinuspersici	http://www.fishbase.se/tools/UploadPhoto/uploads/Torpedosinuspersici.JPG	2025-06-15
Torpedo_torpedo	https://fishbase.se/tools/display_image.php?fw=n&imgName=1346594650_87.1.111.51.jpg	2025-06-15
Torpedo_torpedo	https://fishbase.org/images/species/Totor_u1.jpg	2025-06-15
Torpedo_torpedo	https://fishbase.se/tools/display_image.php?fw=n&imgName=1471900051_188.114.102.50.jpg	2025-06-15
Torpedo_torpedo	https://fishbase.se/tools/display_image.php?fw=n&imgName=ce72a1fc42_3419.jpg	2025-06-15
Torpedo_torpedo	https://fishbase.se/tools/display_image.php?fw=n&imgName=45d4cbef6e_3424.jpg	2025-06-15
Torpedo_torpedo	https://fishbase.se/tools/display_image.php?fw=n&imgName=7865297e64_3421.jpg	2025-06-15
Torpedo_torpedo	https://inaturalist-open-data.s3.amazonaws.com/photos/417406780/large.jpeg	2025-06-15
Torpedo_torpedo	https://static.inaturalist.org/photos/35860360/large.jpeg	2025-06-15
Torpedo_torpedo	https://static.inaturalist.org/photos/416503799/large.jpeg	2025-06-15
Torpedo_torpedo	http://www.fishbase.org/photos/PicturesSummary.php?StartRow=0&ID=2062&what=species&TotRec=3	2025-06-15
Torpedo_torpedo	https://www.floridamuseum.ufl.edu/wp-content/uploads/sites/66/2017/05/Torpedo-torpedo-02.jpg	2025-06-15
Trygonoptera_galba	https://fishesofaustralia.net.au/Images/Image/TrygonopteraGalbaCSIRO.jpg	2025-06-15
Trygonoptera_imitata	https://www.fishbase.se/images/species/Trimi_u0.jpg	2025-06-15
Trygonoptera_imitata	https://shark-references.com/images/species/Trygonoptera_imitata_main.jpg	2025-06-15
Trygonoptera_imitata	https://fishesofaustralia.net.au/images/image/TrygonoptImitatSaschaSchultz.jpg	2025-06-15
Trygonoptera_imitata	https://fishesofaustralia.net.au/Images/Image/TrygonopteraImitataRLS.jpg	2025-06-15
Trygonoptera_imitata	https://fishesofaustralia.net.au/Images/Image/TrygonopteraImitata2RLS.jpg	2025-06-15
Trygonoptera_imitata	https://inaturalist-open-data.s3.amazonaws.com/photos/218726383/large.jpg	2025-06-15
Trygonoptera_imitata	https://inaturalist-open-data.s3.amazonaws.com/photos/12612431/medium.jpeg	2025-06-15
Trygonoptera_mucosa	http://fishesofaustralia.net.au/images/image/TrygonopMucosaRK.jpg	2025-06-15
Trygonoptera_mucosa	http://www.fishbase.se/images/species/Trmuc_u0.jpg	2025-06-15
Trygonoptera_ovalis	http://fishesofaustralia.net.au/Images/Image/TrygonopteraOvalisCSIRO.jpg	2025-06-15
Trygonoptera_ovalis	http://fishesofaustralia.net.au/Images/Image/TrygonopteraOvalisRK.jpg	2025-06-15
Trygonoptera_ovalis	http://fishesofaustralia.net.au/images/image/TrygonoptOvalBenJones.jpg	2025-06-15
Trygonoptera_ovalis	http://www.fishbase.se/images/species/Trova_u5.jpg	2025-06-15

Trygonoptera_personata	http://fishesofaustralia.net.au/images/image/TrygonopteraPersonataCSIRO.jpg	2025-06-15
Trygonoptera_personata	http://www.fishbase.se/images/species/Trper_u0.jpg	2025-06-15
Trygonoptera_testacea	http://fishesofaustralia.net.au/Images/Image/TrygonopteraTestaceaRLS.jpg	2025-06-15
Trygonoptera_testacea	http://fishesofaustralia.net.au/Images/Image/TrygonopTestac2IanShaw.jpg	2025-06-15
Trygonoptera_testacea	http://fishesofaustralia.net.au/Images/Image/TrygonopTestac2seakangaroo.jpg	2025-06-15
Trygonoptera_testacea	http://fishesofaustralia.net.au/Images/Image/TrygonopTestacIanShaw.jpg	2025-06-15
Trygonoptera_testacea	http://fishesofaustralia.net.au/Images/Image/TrygonopTestacJohnTurnbull.jpg	2025-06-15
Trygonoptera_testacea	http://fishesofaustralia.net.au/Images/Image/TrygonopTestacSaschaSchultz.jpg	2025-06-15
Trygonoptera_testacea	http://www.fishbase.se/images/species/Trtes_u0.jpg	2025-06-15
Trygonorrhina_dumerilii	http://fishesofaustralia.net.au/images/image/TDumeriliiRK.jpg	2025-06-15
Trygonorrhina_dumerilii	http://www.inaturalist.org/photos/6225679	2025-06-15
Trygonorrhina_fasciata	http://fishesofaustralia.net.au/images/image/TrygonorrhinaFasciataRLS.jpg	2025-06-15
Trygonorrhina_fasciata	http://www.fishbase.se/tools/UploadPhoto/uploads/Trygonorhinafasciata.jpg	2025-06-15
Urobatis_concentricus	http://www.fishbase.se/images/species/Urcon_u2.jpg	2025-06-15
Urobatis_concentricus	https://static.inaturalist.org/photos/2076188/medium.JPG?1435823125	2025-06-15
Urobatis_concentricus	https://static.inaturalist.org/photos/7443976/medium.jpg?1493164107	2025-06-15
Urobatis_halleri	http://www.boldsystems.org/pics/BACQ/CH663-H11%2B1472863104.JPG	2025-06-15
Urobatis_halleri	http://www.fishbase.se/images/species/Urhal_u0.jpg	2025-06-15
Urobatis_halleri	http://www.fishbase.se/images/species/Urhal_u1.jpg	2025-06-15
Urobatis_halleri	https://farm3.staticflickr.com/2286/2063412438_14dc75faec.jpg	2025-06-15
Urobatis_halleri	https://static.inaturalist.org/photos/1381312/medium.jpg?1444716632	2025-06-15
Urobatis_halleri	https://static.inaturalist.org/photos/6429768/medium.jpg?1488414579	2025-06-15
Urobatis_jamaicensis	http://shark-references.com/images/species/1024px-Yellow_Stingray,_Belize,_2007-09.jpg	2025-06-15
Urobatis_jamaicensis	http://www.boldsystems.org/pics/MEFM/MEFM680-06%2B1149694472.JPG	2025-06-15
Urobatis_jamaicensis	http://www.boldsystems.org/pics/MXIII/MXIII442%2B1248268354.jpg	2025-06-15
Urobatis_jamaicensis	http://www.boldsystems.org/pics/MXIII/MXIII444%2B1245864068.jpg	2025-06-15
Urobatis_jamaicensis	http://www.boldsystems.org/pics/MXIII/MXIII506%2B1245864042.jpg	2025-06-15
Urobatis_jamaicensis	http://www.boldsystems.org/pics/MXV/MXV265%2B1299684576.JPG	2025-06-15
Urobatis_jamaicensis	http://www.fishbase.se/images/species/Urjam_u0.jpg	2025-06-15
Urobatis_jamaicensis	http://www.fishbase.se/images/species/Urjam_u1.jpg	2025-06-15
Urobatis_jamaicensis	http://www.fishbase.se/images/species/Urjam_u2.jpg	2025-06-15
Urobatis_jamaicensis	http://www.fishbase.se/images/species/Urjam_u4.jpg	2025-06-15
Urobatis_jamaicensis	https://static.inaturalist.org/photos/4977573/medium.jpeg?1474601352	2025-06-15
Urobatis_maculatus	http://www.fishbase.se/images/species/Urmac_u2.jpg	2025-06-15
Urobatis_maculatus	https://static.inaturalist.org/photos/4593342/medium.jpeg?1471472448	2025-06-15
Urobatis_pardalis	https://www.fishbase.se/images/species/Urpar_u0.jpg	2025-06-15
Urobatis_pardalis	http://shark-references.com/images/species/U.%20pardalis_Embryo.jpg	2025-06-15
Urobatis_tumbesensis	http://shark-references.com/images/species/Urobatis%20tumbesensis.jpg	2025-06-15
Urobatis_tumbesensis	http://www.fishbase.se/images/species/Urtum_u0.jpg	2025-06-15
Urogymnus_acanthobothrium	http://shark-references.com/images/species/Urogymnus_acanthobothrium_main.jpg	2025-06-15
Urogymnus_asperrimus	http://www.fishbase.se/images/species/Urasp_u0.jpg	2025-06-15
Urogymnus_asperrimus	http://www.fishbase.se/images/species/Urasp_u1.jpg	2025-06-15

Urogymnus_asperrimus	http://www.fishbase.se/photos/workimagethumb.php?s=http://www.fishbase.se/tools/UploadPhoto/uploads/1383626148_182.52.68.117.jpg&w=600	2025-06-15
Urogymnus_asperrimus	http://www.fishbase.se/photos/workimagethumb.php?s=http://www.fishbase.se/tools/UploadPhoto/uploads/1414324605_79.238.54.32.jpg&w=600	2025-06-15
Urogymnus_asperrimus	https://static.inaturalist.org/photos/1991716/medium.jpg?1444755511	2025-06-15
Urogymnus_dalyensis	http://fishesofaustralia.net.au/Images/Image/HimantDalyensRichardPillans.jpg	2025-06-15
Urogymnus_dalyensis	http://fishesofaustralia.net.au/Images/Image/Himantura%20dalyensis%20David%20Wilson%20altB.jpg	2025-06-15
Urogymnus_dalyensis	http://fishesofaustralia.net.au/Images/Image/HimanturDalyensBradPusey.jpg	2025-06-15
Urogymnus_dalyensis	http://fishesofaustralia.net.au/images/image/UrogymDalyensBradPusey.jpg	2025-06-15
Urogymnus_dalyensis	http://shark-references.com/images/species/Himantura_dalyensis_first.jpg	2025-06-15
Urogymnus_dalyensis	http://shark-references.com/images/species/Himantura-dalyensis_Peter_Kyne.jpg	2025-06-15
Urogymnus_dalyensis	http://www.fishbase.se/images/species/Hidal_u0.jpg	2025-06-15
Urogymnus_granulatus	http://shark-references.com/images/species/12586_Higra_u0.jpg	2025-06-15
Urogymnus_granulatus	http://shark-references.com/images/species/Himantura%20granulata_DAVY%20(2).jpg	2025-06-15
Urogymnus_granulatus	http://shark-references.com/images/species/Himantura%20granulata_DAVY%20(3).jpg	2025-06-15
Urogymnus_granulatus	http://shark-references.com/images/species/Photo1396.jpg	2025-06-15
Urolophus_aurantiacus	http://www.fishbase.se/images/species/Uraur_u0.jpg	2025-06-15
Urolophus_aurantiacus	http://www.fishbase.se/images/species/Uraur_u1.jpg	2025-06-15
Urolophus_aurantiacus	https://www.fishbase.se/images/species/Uraur_u2.jpg	2025-06-15
Urolophus_bucculentus	http://fishesofaustralia.net.au/images/image/UrolophusBucculentusKHG.jpg	2025-06-15
Urolophus_circularis	http://fishesofaustralia.net.au/images/image/UrolophCircularis.jpg	2025-06-15
Urolophus_cruciatus	http://fishesofaustralia.net.au/images/image/UrolophusCruciatus.jpg	2025-06-15
Urolophus_cruciatus	http://fishesofaustralia.net.au/Images/Image/UrolophusCruciatus2RLS.jpg	2025-06-15
Urolophus_cruciatus	http://fishesofaustralia.net.au/Images/Image/UrolophusCruciatus3.jpg	2025-06-15
Urolophus_cruciatus	http://fishesofaustralia.net.au/Images/Image/UrolophusCruciatusRLS.jpg	2025-06-15
Urolophus_cruciatus	http://shark-references.com/images/species/Banded-stingaree.jpg	2025-06-15
Urolophus_deforgesi	http://www.boldsystems.org/pics/ANCC/BPS0305A%2B1025823878.JPG	2025-06-15
Urolophus_deforgesi	http://www.boldsystems.org/pics/ANCC/BPS0507A%2B1022806836.JPG	2025-06-15
Urolophus_flavomosaicus	http://fishesofaustralia.net.au/images/image/UrolophusflavomosaicusCSIRO.jpg	2025-06-15
Urolophus_gigas	http://fishesofaustralia.net.au/Images/Image/UrolophGigasSaschaSchultz.jpg	2025-06-15
Urolophus_gigas	http://fishesofaustralia.net.au/Images/Image/UrolophusGigasDHarasti.jpg	2025-06-15
Urolophus_gigas	http://fishesofaustralia.net.au/images/image/UrolophusGigasJMontana.jpg	2025-06-15
Urolophus_gigas	http://fishesofaustralia.net.au/Images/Image/UrolophusGigasRK.jpg	2025-06-15
Urolophus_gigas	http://www.fishbase.se/images/species/Urgig_u0.jpg	2025-06-15
Urolophus_gigas	http://www.fishbase.se/images/species/Urgig_u1.jpg	2025-06-15
Urolophus_gigas	https://farm4.staticflickr.com/3544/3414930912_733f18765a.jpg	2025-06-15
Urolophus_kapalensis	http://fishesofaustralia.net.au/Images/Image/UrolophKapalensDaveHarasti.jpg	2025-06-15
Urolophus_kapalensis	http://fishesofaustralia.net.au/Images/Image/UrolophusKapalensisCSIRO.jpg	2025-06-15
Urolophus_kapalensis	http://fishesofaustralia.net.au/images/image/UrolophusKapalensisRLing.jpg	2025-06-15
Urolophus_kapalensis	http://fishesofaustralia.net.au/Images/Image/UrolophusKapalensisTasoViglas.jpg	2025-06-15
Urolophus_lobatus	http://fishesofaustralia.net.au/images/image/UrolophusLobatusCSIRO.jpg	2025-06-15
Urolophus_mitosis	http://fishesofaustralia.net.au/images/image/UrolophusMitosisCSIRO.jpg	2025-06-15
Urolophus_orarius	http://fishesofaustralia.net.au/Images/Image/UrolophusOrariusCSIRO.jpg	2025-06-15
Urolophus_orarius	http://fishesofaustralia.net.au/images/image/UrolophusOrariusDHarasti.jpg	2025-06-15
Urolophus_orarius	http://www.fishbase.se/images/species/Urora_u0.jpg	2025-06-15

Urolophus_papilio	http://www.boldsystems.org/pics/ANCC/BPS0506A%2B1024392366.JPG	2025-06-15
Urolophus_paucimaculatus	http://fishesofaustralia.net.au/Images/Image/UrolophusPaucimaculatus.jpg	2025-06-15
Urolophus_paucimaculatus	http://fishesofaustralia.net.au/Images/Image/UrolophusPaucimaculatusCSIRO.jpg	2025-06-15
Urolophus_paucimaculatus	http://fishesofaustralia.net.au/Images/Image/UrolophusPausimaculatus3JKF.jpg	2025-06-15
Urolophus_viridis	http://fishesofaustralia.net.au/images/image/UrolophViridisCSIRO.jpg	2025-06-15
Urolophus_viridis	http://fishesofaustralia.net.au/Images/Image/UrolophViridisKG.jpg	2025-06-15
Urotrygon_aspidura	http://www.boldsystems.org/pics/SEMAR/SEMAR-051%2B1272476054.jpg	2025-06-15
Urotrygon_aspidura	http://www.boldsystems.org/pics/TCBAT/TI_29May15_Uasp03_A%2B1474670479.jpg	2025-06-15
Urotrygon_aspidura	http://www.boldsystems.org/pics/TCBAT/TI_29May15_Uasp04_A%2B1474670541.jpg	2025-06-15
Urotrygon_aspidura	http://www.boldsystems.org/pics/TCBAT/Ti_7Sep14_Uasp02_A%2B1474670422.jpg	2025-06-15
Urotrygon_aspidura	http://www.boldsystems.org/pics/TCBAT/Ti_7Sep14_Uasp02_A%2B1474670422.jpg	2025-06-15
Urotrygon_aspidura	http://www.fishbase.se/images/species/Urasp_u3.jpg	2025-06-15
Urotrygon_aspidura	http://www.fishbase.se/images/species/Urasp_u4.jpg	2025-06-15
Urotrygon_chilensis	http://shark-references.com/images/species/urotrygon_chilensis_01.jpg	2025-06-15
Urotrygon_chilensis	http://shark-references.com/images/species/Urotrygon_chilensis_02.jpg	2025-06-15
Urotrygon_chilensis	http://shark-references.com/images/species/Urotrygon_chilensis_03.jpg	2025-06-15
Urotrygon_chilensis	http://shark-references.com/images/species/Urotrygon_chilensis.jpg	2025-06-15
Urotrygon_chilensis	http://www.boldsystems.org/pics/BPMII/IB-PACIFICO026%2B1355428710.jpg	2025-06-15
Urotrygon_chilensis	http://www.boldsystems.org/pics/MBMAP/PR2016A61-2_%5Ba%5D%2B1474123840.JPG	2025-06-15
Urotrygon_chilensis	http://www.boldsystems.org/pics/MBMAP/PR2016A61-2_%5Bb%5D%2B1474123838.JPG	2025-06-15
Urotrygon_chilensis	http://www.fishbase.se/images/species/Urchi_u1.jpg	2025-06-15
Urotrygon_chilensis	http://www.fishbase.se/images/species/Urchi_u2.jpg	2025-06-15
Urotrygon_chilensis	http://www.fishbase.se/images/species/Urchi_u4.jpg	2025-06-15
Urotrygon_chilensis	http://www.fishbase.se/photos/workimagethumb.php?s=http://www.fishbase.se/tools/UploadPhoto/uploads/1486638431_162.158.59.12.jpg&w=600	2025-06-15
Urotrygon_cimar	https://www.fishbase.se/images/species/Urcim_u0.jpg	2025-06-15
Urotrygon_cimar	https://www.fishbase.se/images/species/Urcim_u1.jpg	2025-06-15
Urotrygon_microphthalmum	http://shark-references.com/images/species/Urotrygon-microphthalmum-main.jpg	2025-06-15
Urotrygon_microphthalmum	http://www.fishbase.se/images/species/Urmic_m0.jpg	2025-06-15
Urotrygon_microphthalmum	http://www.fishbase.se/images/species/Urmic_u1.jpg	2025-06-15
Urotrygon_munda	http://shark-references.com/images/species/Urotrygon_asterias.jpg	2025-06-15
Urotrygon_munda	http://www.fishbase.se/images/species/Urmun_u1.jpg	2025-06-15
Urotrygon_nana	http://www.discoverlife.org/IM/I_RR/0001/640/Urotrygon_nana,I_RR185.jpg	2025-06-15
Urotrygon_nana	http://www.fishbase.se/images/species/Urnan_u0.jpg	2025-06-15
Urotrygon_reticulata	http://www.fishbase.se/images/species/Urret_u0.jpg	2025-06-15
Urotrygon_rogersi	http://shark-references.com/images/species/Urotrygon_rogersi.jpg	2025-06-15
Urotrygon_rogersi	http://www.boldsystems.org/pics/OXF/OXF176%2B1374338378.JPG	2025-06-15
Urotrygon_rogersi	http://www.boldsystems.org/pics/OXF/OXF177%2B1374338360.JPG	2025-06-15
Urotrygon_rogersi	http://www.boldsystems.org/pics/OXF/OXF178%2B1374338348.JPG	2025-06-15
Urotrygon_rogersi	http://www.boldsystems.org/pics/OXF/OXF179%2B1374338314.JPG	2025-06-15
Urotrygon_rogersi	http://www.boldsystems.org/pics/OXF/OXF180%2B1366245772.JPG	2025-06-15
Urotrygon_rogersi	http://www.boldsystems.org/pics/SEMAR/SEMAR-163%2B1274311778.jpg	2025-06-15
Urotrygon_rogersi	http://www.boldsystems.org/pics/TCBAT/TI_28May15_Urog01_A%2B1474670635.jpg	2025-06-15
Urotrygon_rogersi	http://www.boldsystems.org/pics/TCBAT/TI_28May15_Urog02_A%2B1474670700.jpg	2025-06-15

Urotrygon_rogersi	http://www.boldsystems.org/pics/TCBAT/TI_28May15_Urog04_A%2B1474670805.jpg	2025-06-15
Urotrygon_rogersi	http://www.fishbase.se/images/species/Urrog_u0.jpg	2025-06-15
Urotrygon_rogersi	http://www.fishbase.se/images/species/Urrog_u1.jpg	2025-06-15
Urotrygon_rogersi	http://www.fishbase.se/images/species/Urrog_u2.jpg	2025-06-15
Urotrygon_rogersi	https://static.inaturalist.org/photos/7443877/medium.jpg?1493163902	2025-06-15
Urotrygon_simulatrix	http://www.fishbase.se/images/species/Ursim_u0.jpg	2025-06-15
Zanobatus_maculatus	http://shark-references.com/images/species/thumbnail/Zanobatus-maculatus_holotype.jpg	2025-06-15
Zanobatus_schoenleinii	http://www.fishbase.se/images/species/Zasch_u0.jpg	2025-06-15
Zanobatus_schoenleinii	http://www.fishbase.se/images/species/Zasch_u1.jpg	2025-06-15
Zanobatus_schoenleinii	http://www.fishbase.se/tools/UploadPhoto/uploads/1383146287_88.136.141.220.jpg	2025-06-15
Zapteryx_brevirostris	http://shark-references.com/images/species/Zabre_u0.jpg	2025-06-15
Zapteryx_brevirostris	http://www.boldsystems.org/pics/FARAN/UNMDP_DI_2159%2B1345212766.JPG	2025-06-15
Zapteryx_brevirostris	http://www.boldsystems.org/pics/FARAN/UNMDP_DI_2159%2B1345212766.JPG	2025-06-15
Zapteryx_brevirostris	http://www.fishbase.se/images/species/Zabre_f0.jpg	2025-06-15
Zapteryx_brevirostris	http://www.fishbase.se/images/species/Zabre_j0.jpg	2025-06-15
Zapteryx_brevirostris	http://www.fishbase.se/images/species/Zabre_j1.jpg	2025-06-15
Zapteryx_brevirostris	http://www.fishbase.se/images/species/Zabre_u0.jpg	2025-06-15
Zapteryx_brevirostris	http://www.fishbase.se/photos/workimagethumb.php?s=http://www.fishbase.se/tools/UploadPhoto/uploads/1346244747_200.128.60.77.jpg&w=600	2025-06-15
Zapteryx_brevirostris	http://www.fishbase.se/tools/UploadPhoto/uploads/Zapteryx_brevirostris.jpg	2025-06-15
Zapteryx_exasperata	http://www.fishbase.se/images/species/Zaexa_u0.jpg	2025-06-15
Zapteryx_exasperata	http://www.fishbase.se/images/species/Zaexa_u1.jpg	2025-06-15
Zapteryx_xyster	http://shark-references.com/images/species/Zapteryx_xyster_2.jpg	2025-06-15
Zapteryx_xyster	http://www.fishbase.se/images/species/Zaxys_u0.jpg	2025-06-15
Zapteryx_xyster	http://www.fishbase.se/images/species/Zaxys_u1.jpg	2025-06-15
Zearaja_chilensis	https://inaturalist-open-data.s3.amazonaws.com/photos/185841424/square.jpeg	2025-06-15
Zearaja_chilensis	https://inaturalist-open-data.s3.amazonaws.com/photos/11327651/square.jpeg	2025-06-15
Zearaja_chilensis	https://bench.boldsystems.org/pics/FARG/INIDEP-_DI_0593%2B1214409746.jpg	2025-06-15
Zearaja_maugeana	http://fishesofaustralia.net.au/images/image/ZearajMaugeana2.jpg	2025-06-15
Zearaja_maugeana	http://www.fishbase.se/images/species/Zemau_u0.jpg	2025-06-15
Zearaja_nasuta	http://www.fishbase.se/images/species/Dinas_u0.jpg	2025-06-15
Zearaja_nasuta	http://www.fishbase.se/images/species/Zenas_u0.jpg	2025-06-15
Zearaja_nasuta	http://www.fishbase.se/tools/UploadPhoto/uploads/DSC03563copy.JPG	2025-06-15
Zearaja_nasuta	https://static.inaturalist.org/photos/164506/medium.jpg?1444936222	2025-06-15

Supplemental Table 5. Assessing the consistency of scoring the presence/absence of conspicuous markings from Last et al. (2016)* compared to photographs of each species taken from images found on various websites. See Supplemental Table 4 for a list of websites used. For each species, whether conspicuous markings were present (Yes) or absent (No) when drawings were scored from Last et al. (2016)* are compared with whether conspicuous markings were present (Yes) or absent (No) in photographs is noted. For each species the number of photographs assessed is also noted.

* Last, P. R., White, W.T., de Carvalho, M.R., Séret, B., Stehmann, M.F.W., and Naylor, G.J.P. (2016). *Rays of the World*. CSIRO Publishing.

**Species**	**Conspicuous marking in Last et al. (2016)***	**Conspicuous marking in photographs**	**Number of photos**
Amblyraja_hyperborea	No	No	8
Cruriraja_parcomaculata	No	No	8
Leucoraja_wallacei	No	No	7
Raja_clavata	No	No	14
Raja_undulata	No	No	9
Gymnura_crebripunctata	No	No	1
Gymnura_japonica	No	No	2
Malacoraja_senta	No	No	1
Rajella_leopardus	No	No	5
Rhinobatos_rhinobatos	No	No	3
Gurgesiella_dorsalifera	No	No	3
Acroteriobatus_salalah	No	No	1
Acroteriobatus_variegatus	No	No	1
Aetobatus_flagellum	No	No	3
Aetobatus_narinari	No	No	6
Aetomylaeus_nichofii	No	No	8
Aetomylaeus_vespertilio	No	No	5
Amblyraja_doellojuradoi	No	No	9
Amblyraja_frerichsi	No	No	2
Amblyraja_georgiana	No	No	4
Amblyraja_jenseni	No	No	5
Amblyraja_radiata	No	No	8
Anoxypristis_cuspidata	No	No	2
Aptychotrema_rostrata	No	No	6
Aptychotrema_timorensis	No	No	1
Aptychotrema_vincentiana	No	No	1
Atlantoraja_castelnaui	No	No	13
Bathyraja_abyssicola	No	No	2
Bathyraja_brachyurops	No	No	6
Bathyraja_eatonii	No	No	2
Bathyraja_griseocauda	No	No	5

Bathyraja_isotrachys	No	No	1
Bathyraja_leucomelanos	No	No	1
Bathyraja_matsubarai	No	No	1
Bathyraja_meridionalis	No	No	4
Bathyraja_microtrachys	No	No	2
Bathyraja_minispinosa	No	No	1
Bathyraja_pallida	No	No	1
Bathyraja_papilionifera	No	No	1
Bathyraja_peruana	No	No	2
Bathyraja_richardsoni	No	No	4
Bathyraja_scaphiops	No	No	8
Bathyraja_schroederi	No	No	1
Bathyraja_smithii	No	No	3
Bathyraja_spinicauda	No	No	8
Bathyraja_spinosissima	No	No	4
Bathyraja_trachura	No	No	9
Bathyraja_violacea	No	No	5
Benthobatis_kreffti	No	No	2
Benthobatis_marcida	No	No	1
Benthobatis_moresbyi	No	No	3
Benthobatis_yangi	No	No	1
Breviraja_spinosa	No	No	2
Brochiraja_heuresa	No	No	1
Brochiraja_vittacauda	No	No	1
Cruriraja_hulleyi	No	No	1
Dactylobatus_clarkii	No	No	2
Dasyatis_acutirostra	No	No	1
Dasyatis_akajei	No	No	1
Dasyatis_americana	No	No	3
Dasyatis_bennetti	No	No	7
Dasyatis_brevicaudata	No	No	4
Dasyatis_centroura	No	No	5
Dasyatis_chrysonota	No	No	2
Dasyatis_dipterura	No	No	1
Dasyatis_fluviorum	No	No	1
Dasyatis_geijskesi	No	No	1
Dasyatis_guttata	No	No	11
Dasyatis_hypostigma	No	No	9
Dasyatis_izuensis	No	No	2
Dasyatis_laosensis	No	No	2
Dasyatis_lata	No	No	6
Dasyatis_longa	No	No	4
Dasyatis_margarita	No	No	1
Dasyatis_margaritella	No	No	1
Dasyatis_marianae	No	No	4

Dasyatis_marmorata	No	No	3
Dasyatis_microps	No	No	1
Dasyatis_navarrae	No	No	1
Dasyatis_parvonigra	No	No	2
Dasyatis_pastinaca	No	No	10
Dasyatis_sabina	No	No	4
Dasyatis_say	No	No	6
Dasyatis_tortonesei	No	No	5
Dasyatis_zugei	No	No	5
Diplobatis_pictus	No	No	1
Dipturus_acrobelus	No	No	4
Dipturus_apricus	No	No	2
Dipturus_australis	No	No	1
Dipturus_bullisi	No	No	1
Dipturus_canutus	No	No	2
Dipturus_cerva	No	No	1
Dipturus_confusus	No	No	2
Dipturus_endeavouri	No	No	2
Dipturus_grahami	No	No	2
Dipturus_gudgeri	No	No	2
Dipturus_healdi	No	No	2
Dipturus_johannisdavesi	No	No	2
Dipturus_kwangtungensis	No	No	2
Dipturus_macrocauda	No	No	1
Dipturus_melanospilus	No	No	3
Dipturus_nidarosiensis	No	No	5
Dipturus_oregoni	No	No	1
Dipturus_polyommata	No	No	2
Dipturus_queenslandicus	No	No	1
Dipturus_teevani	No	No	2
Dipturus_tengu	No	No	1
Dipturus_trachydermus	No	No	1
Dipturus_wengi	No	No	2
Discopyge_tschudii	No	No	7
Electrolux_addisoni	No	No	1
Fenestraja_ishiyamai	No	No	1
Fenestraja_plutonia	No	No	1
Glaucostegus_cemiculus	No	No	4
Glaucostegus_granulatus	No	No	6
Glaucostegus_halavi	No	No	4
Glaucostegus_obtusus	No	No	6
Glaucostegus_thouin	No	No	3
Glaucostegus_typus	No	No	4
Gurgesiella_furvescens	No	No	2
Gymnura_altavela	No	No	4

Gymnura_marmorata	No	No	5
Gymnura_micrura	No	No	10
Gymnura_natalensis	No	No	3
Gymnura_poecilura	No	No	12
Gymnura_zonura	No	No	2
Hexatrygon_bickelli	No	No	3
Himantura_astra	No	No	3
Himantura_dalyensis	No	No	7
Himantura_fai	No	No	6
Himantura_gerrardi	No	No	6
Himantura_granulata	No	No	4
Himantura_imbricata	No	No	3
Himantura_jenkinsii	No	No	2
Himantura_kittipongi	No	No	1
Himantura_leoparda	No	No	11
Himantura_pacifica	No	No	2
Himantura_pastinacoides	No	No	1
Himantura_randalli	No	No	2
Himantura_schmardae	No	No	2
Himantura_signifer	No	No	3
Himantura_toshi	No	No	1
Himantura_uarnacoides	No	No	1
Himantura_uarnak	No	No	12
Himantura_undulata	No	No	8
Himantura_walga	No	No	4
Hypnos_monopterygius	No	No	5
Insentiraja_laxipella	No	No	1
Insentiraja_subtilispinosa	No	No	2
Leucoraja_circularis	No	No	7
Leucoraja_fullonica	No	No	5
Leucoraja_lentiginosa	No	No	1
Manta_alfredi	No	No	3
Manta_birostris	No	No	5
Mobula_hypostoma	No	No	4
Mobula_kuhlii	No	No	5
Mobula_mobular	No	No	5
Mobula_munkiana	No	No	2
Mobula_tarapacana	No	No	4
Mobula_thurstoni	No	No	5
Myliobatis_aquila	No	No	10
Myliobatis_californicus	No	No	4
Myliobatis_chilensis	No	No	1
Myliobatis_freminvillei	No	No	3
Myliobatis_goodei	No	No	8
Myliobatis_hamlyni	No	No	2

Myliobatis_longirostris	No	No	1
Myliobatis_peruvianus	No	No	1
Myliobatis_ridens	No	No	3
Myliobatis_tenuicaudatus	No	No	4
Narcine_bancroftii	No	No	4
Narcine_brasiliensis	No	No	6
Narcine_brevilabiata	No	No	2
Narcine_entemedor	No	No	6
Narcine_lasti	No	No	1
Narcine_leoparda	No	No	1
Narcine_lingula	No	No	2
Narcine_nelsoni	No	No	1
Narcine_ornata	No	No	1
Narcine_prodorsalis	No	No	2
Narcine_tasmaniensis	No	No	5
Narcine_timlei	No	No	3
Narcine_vermiculatus	No	No	3
Narcine_westraliensis	No	No	2
Narke_capensis	No	No	1
Narke_japonica	No	No	2
Neoraja_iberica	No	No	3
Neotrygon_kuhlii	No	No	10
Neotrygon_picta	No	No	3
Notoraja_alisae	No	No	1
Notoraja_azurea	No	No	2
Notoraja_hirticauda	No	No	1
Notoraja_lira	No	No	1
Notoraja_sapphira	No	No	1
Notoraja_sticta	No	No	2
Okamejei_leptoura	No	No	4
Paratrygon_aiereba	No	No	2
Pastinachus_atrus	No	No	7
Pastinachus_gracilicaudus	No	No	2
Pastinachus_sephen	No	No	7
Pastinachus_solocirostris	No	No	1
Pastinachus_stellurostris	No	No	2
Pavoraja_alleni	No	No	1
Pavoraja_mosaica	No	No	1
Pavoraja_nitida	No	No	1
Pavoraja_pseudonitida	No	No	1
Pavoraja_umbrosa	No	No	1
Platyrhina_hyugaensis	No	No	1
Platyrhina_sinensis	No	No	2
Platyrhina_tangi	No	No	4
Platyrhinoidis_triseriata	No	No	2

Plesiobatis_daviesi	No	No	4
Potamotrygon_brachyura	No	No	4
Potamotrygon_falkneri	No	No	6
Potamotrygon_henlei	No	No	2
Potamotrygon_humerosa	No	No	1
Potamotrygon_hystrix	No	No	5
Potamotrygon_leopoldi	No	No	7
Potamotrygon_magdalenae	No	No	3
Potamotrygon_marinae	No	No	1
Potamotrygon_motoro	No	No	12
Potamotrygon_orbignyi	No	No	7
Potamotrygon_schroederi	No	No	4
Potamotrygon_scobina	No	No	3
Potamotrygon_signata	No	No	1
Potamotrygon_tigrina	No	No	1
Potamotrygon_yepezi	No	No	2
Pristis_clavata	No	No	2
Pristis_pectinata	No	No	2
Pristis_pristis	No	No	4
Pristis_zijsron	No	No	1
Psammobatis_lentiginosa	No	No	9
Psammobatis_normani	No	No	12
Psammobatis_scobina	No	No	5
Pteromylaeus_bovinus	No	No	3
Pteroplatytrygon_violacea	No	No	12
Raja_asterias	No	No	6
Raja_eglanteria	No	No	2
Raja_maderensis	No	No	1
Raja_microocellata	No	No	2
Rajella_barnardi	No	No	4
Rajella_bathyphila	No	No	6
Rajella_bigelowi	No	No	1
Rajella_challengeri	No	No	3
Rajella_dissimilis	No	No	4
Rajella_kukujevi	No	No	2
Rajella_lintea	No	No	1
Rajella_nigerrima	No	No	1
Rajella_purpuriventralis	No	No	1
Rajella_sadowskii	No	No	3
Rhinobatos_albomaculatus	No	No	1
Rhinobatos_annandalei	No	No	1
Rhinobatos_glaucostigma	No	No	3
Rhinobatos_holcorhynchus	No	No	1
Rhinobatos_horkelii	No	No	4
Rhinobatos_hynnicephalus	No	No	1

Rhinobatos_lentiginosus	No	No	4
Rhinobatos_leucorhynchus	No	No	2
Rhinobatos_percellens	No	No	6
Rhinobatos_planiceps	No	No	2
Rhinobatos_prahli	No	No	3
Rhinobatos_productus	No	No	6
Rhinobatos_punctifer	No	No	6
Rhinobatos_sainsburyi	No	No	1
Rhinobatos_schlegelii	No	No	2
Rhinoptera_bonasus	No	No	4
Rhinoptera_brasiliensis	No	No	2
Rhinoptera_javanica	No	No	4
Rhinoptera_jayakari	No	No	3
Rhinoptera_marginata	No	No	1
Rhinoptera_neglecta	No	No	2
Rhinoptera_steindachneri	No	No	2
Rhinoraja_macloviana	No	No	1
Rhinoraja_magellanica	No	No	3
Rhinoraja_murrayi	No	No	5
Sinobatis_borneensis	No	No	1
Sinobatis_bulbicauda	No	No	2
Sinobatis_caerulea	No	No	1
Spiniraja_whitleyi	No	No	6
Sympterygia_acuta	No	No	12
Taeniura_lymma	No	No	14
Taeniurops_grabata	No	No	5
Taeniurops_meyeni	No	No	6
Temera_hardwickii	No	No	2
Torpedo_californica	No	No	4
Torpedo_formosa	No	No	1
Torpedo_fuscomaculata	No	No	7
Torpedo_marmorata	No	No	6
Torpedo_nobiliana	No	No	4
Torpedo_panthera	No	No	9
Torpedo_puelcha	No	No	1
Torpedo_sinuspersici	No	No	9
Torpedo_tokionis	No	No	2
Torpedo_tremens	No	No	4
Trygonoptera_galba	No	No	1
Trygonoptera_imitata	No	No	7
Trygonoptera_mucosa	No	No	2
Trygonoptera_testacea	No	No	7
Trygonorrhina_dumerilii	No	No	2
Trygonorrhina_fasciata	No	No	2
Urobatis_concentricus	No	No	3

Urobatis_halleri	No	No	6
Urobatis_jamaicensis	No	No	11
Urobatis_maculatus	No	No	2
Urobatis_tumbesensis	No	No	2
Urogymnus_asperrimus	No	No	5
Urolophus_aurantiacus	No	No	3
Urolophus_bucculentus	No	No	1
Urolophus_circularis	No	No	1
Urolophus_cruciatus	No	No	5
Urolophus_deforgesi	No	No	2
Urolophus_flavomosaicus	No	No	1
Urolophus_gigas	No	No	7
Urolophus_kapalensis	No	No	4
Urolophus_lobatus	No	No	1
Urolophus_mitosis	No	No	1
Urolophus_papilio	No	No	1
Urolophus_viridis	No	No	2
Urotrygon_aspidura	No	No	7
Urotrygon_chilensis	No	No	11
Urotrygon_cimar	No	No	2
Urotrygon_microphthalmum	No	No	3
Urotrygon_munda	No	No	2
Urotrygon_nana	No	No	2
Urotrygon_reticulata	No	No	1
Urotrygon_rogersi	No	No	14
Urotrygon_simulatrix	No	No	1
Zanobatus_schoenleinii	No	No	3
Zapteryx_brevirostris	No	No	9
Zapteryx_exasperata	No	No	2
Zapteryx_xyster	No	No	3
Bathyraja_aleutica	No	Yes	2
Dipturus_oxyrinchus	No	Yes	8
Leucoraja_garmani	No	Yes	5
Psammobatis_rutrum	No	Yes	5
Rhinoraja_albomaculata	No	Yes	6
Zearaja_maugeana	No	Yes	2
Dipturus_olseni	No	Yes	1
Psammobatis_rudis	No	Yes	13
Irolita_westraliensis	No	Yes	2
Neoraja_caerulea	No	Yes	1
Sympterygia_brevicaudata	No	Yes	3
Sympterygia_lima	No	Yes	3
Leucoraja_erinacea	No	Yes	8
Raja_straeleni	No	Yes	8
Rajella_fyllae	Yes	Yes	12

Bathyraja_cousseauae	Yes	Yes	5
Bathyraja_maccaini	Yes	Yes	2
Dipturus_batis	Yes	Yes	6
Leucoraja_ocellata	Yes	Yes	4
Psammobatis_bergi	Yes	Yes	12
Raja_brachyura	Yes	Yes	6
Raja_rhina	Yes	Yes	2
Rhinoraja_multispinis	Yes	Yes	6
Rostroraja_alba	Yes	Yes	9
Sympterygia_bonapartii	Yes	Yes	7
Zearaja_chilensis	Yes	Yes	3
Zearaja_nasuta	Yes	Yes	4
Atlantoraja_cyclophora	Yes	Yes	12
Bathyraja_maculata	Yes	Yes	1
Bathyraja_mariposa	Yes	Yes	1
Bathyraja_parmifera	Yes	Yes	2
Beringraja_binoculata	Yes	Yes	7
Beringraja_pulchra	Yes	Yes	1
Dentiraja_lemprieri	Yes	Yes	4
Diplobatis_ommata	Yes	Yes	2
Dipturus_falloargus	Yes	Yes	2
Dipturus_laevis	Yes	Yes	6
Dipturus_oculus	Yes	Yes	2
Dipturus_pullopunctata	Yes	Yes	4
Gymnura_australis	Yes	Yes	1
Heteronarce_bentuviai	Yes	Yes	3
Leucoraja_melitensis	Yes	Yes	2
Leucoraja_naevus	Yes	Yes	9
Narke_dipterygia	Yes	Yes	5
Neotrygon_annotata	Yes	Yes	1
Neotrygon_ningalooensis	Yes	Yes	2
Okamejei_boesemani	Yes	Yes	1
Okamejei_cairae	Yes	Yes	1
Okamejei_hollandi	Yes	Yes	1
Okamejei_jensenae	Yes	Yes	8
Okamejei_kenojei	Yes	Yes	1
Raja_equatorialis	Yes	Yes	1
Raja_herwigi	Yes	Yes	1
Raja_inornata	Yes	Yes	1
Raja_miraletus	Yes	Yes	18
Raja_polystigma	Yes	Yes	10
Raja_radula	Yes	Yes	12
Raja_stellulata	Yes	Yes	1
Raja_texana	Yes	Yes	2
Raja_velezi	Yes	Yes	2

Rhinoraja_taranetzi	Yes	Yes	1
Rhynchobatus_laevis	Yes	Yes	7
Rhynchobatus_palpebratus	Yes	Yes	2
Rhynchobatus_springeri	Yes	Yes	2
Rioraja_agassizii	Yes	Yes	6
Torpedo_torpedo	Yes	Yes	11
Trygonoptera_ovalis	Yes	Yes	4
Trygonoptera_personata	Yes	Yes	2
Urolophus_orarius	Yes	Yes	3
Urolophus_paucimaculatus	Yes	Yes	3
Atlantoraja_platana	Yes	Yes	10
Neotrygon_leylandi	Yes	Yes	2
Psammobatis_extenta	Yes	Yes	5
Raja_montagui	Yes	Yes	9
Rhina_ancylostoma	Yes	Yes	7
Rhynchobatus_australiae	Yes	Yes	10
Rhynchobatus_djiddensis	Yes	Yes	2
