# Supplementary material for: The evolution of eyespots in skates and rays
Source: Nat Ecol Evol. 2026 Apr 24;10(6):1184–94. doi: 10.1038/s41559-026-03059-5 (PMC13253327; doi:10.1038/s41559-026-03059-5)
Supplement: Supplementary file 2 — Reporting Summary [file 41559_2026_3059_MOESM2_ESM.pdf]

nature portfolio

Corresponding author(s): John Fitzpatrick

Last updated by author(s): Feb 16, 2026

Reporting Summary

Nature Portfolio wishes to improve the reproducibility of the work that we publish. This form provides structure for consistency and transparency in reporting. For further information on Nature Portfolio policies, see our Editorial Policies and the Editorial Policy Checklist.

Statistics

For all statistical analyses, confirm that the following items are present in the figure legend, table legend, main text, or Methods section.

n/a	Confirmed	
☐	☒	The exact sample size (n) for each experimental group/condition, given as a discrete number and unit of measurement
☐	☒	A statement on whether measurements were taken from distinct samples or whether the same sample was measured repeatedly
☐	☒	The statistical test(s) used AND whether they are one- or two-sided<br>*Only common tests should be described solely by name; describe more complex techniques in the Methods section.*
☐	☒	A description of all covariates tested
☐	☒	A description of any assumptions or corrections, such as tests of normality and adjustment for multiple comparisons
☐	☒	A full description of the statistical parameters including central tendency (e.g. means) or other basic estimates (e.g. regression coefficient) AND variation (e.g. standard deviation) or associated estimates of uncertainty (e.g. confidence intervals)
☐	☒	For null hypothesis testing, the test statistic (e.g. F, t, r) with confidence intervals, effect sizes, degrees of freedom and P value noted<br>*Give P values as exact values whenever suitable.*
☒	☐	For Bayesian analysis, information on the choice of priors and Markov chain Monte Carlo settings
☒	☐	For hierarchical and complex designs, identification of the appropriate level for tests and full reporting of outcomes
☒	☐	Estimates of effect sizes (e.g. Cohen's d, Pearson's r), indicating how they were calculated

Our web collection on statistics for biologists contains articles on many of the points above.

Software and code

Policy information about availability of computer code

Data collection	No code was used to collect data for this study
Data analysis	Analyses were performed in R Studio using R version 4.4.0 (2024-04-24). Code used in the data analysis is available using the following link: https://osf.io/k6cy9/overview?view_only=8d3769f32fbf4a1ba4ffb0f4c26e2c67

For manuscripts utilizing custom algorithms or software that are central to the research but not yet described in published literature, software must be made available to editors and reviewers. We strongly encourage code deposition in a community repository (e.g. GitHub). See the Nature Portfolio guidelines for submitting code & software for further information.

Data

Policy information about availability of data

All manuscripts must include a data availability statement. This statement should provide the following information, where applicable:

- Accession codes, unique identifiers, or web links for publicly available datasets
- A description of any restrictions on data availability
- For clinical datasets or third party data, please ensure that the statement adheres to our policy

Provide your data availability statement here.

Research involving human participants, their data, or biological material

Policy information about studies with human participants or human data. See also policy information about sex, gender (identity/presentation), and sexual orientation and race, ethnicity and racism.

Reporting on sex and gender	Humans were not studied in our work.
Reporting on race, ethnicity, or other socially relevant groupings	Humans were not studied in our work.
Population characteristics	Humans were not studied in our work.
Recruitment	Humans were not studied in our work.
Ethics oversight	Humans were not studied in our work.

Note that full information on the approval of the study protocol must also be provided in the manuscript.

Field-specific reporting

Please select the one below that is the best fit for your research. If you are not sure, read the appropriate sections before making your selection.

☐ Life sciences ☐ Behavioural & social sciences ☒ Ecological, evolutionary & environmental sciences

For a reference copy of the document with all sections, see nature.com/documents/nr-reporting-summary-flat.pdf

Ecological, evolutionary & environmental sciences study design

All studies must disclose on these points even when the disclosure is negative.

Study description	This is a comparative study that assesses evolutionary patterns across skates and rays. Analyses are divided into species with and without conspicuous markings.
Research sample	We scored the presence/absence of conspicuous markings, defined as body markings that diverge from the remaining body pattern, from the dorsal side of 580 skates and rays, representing >90% of extant species from the four Orders of skates and rays, including skates (Order Rajiformes), stingrays and their allies (Order Myliobatiformes), shovelnose rays and their allies (Order Rhinopristiformes) and electric rays (Order Torpediniformes).
Sampling strategy	We assessed all skates and rays that were present in the Chondrichthyan Tree of Life phylogeny (http://sharksrays.org/).
Data collection	Data was collected from publicly available databases (e.g. Fishbase) and from published books and papers. Conspicuous markings were scored from Last et al. 2016's book 'Rays of the World'. MÅ and JLF collected the data.
Timing and spatial scale	Data collection started in August 2023 and continued until September 2025.
Data exclusions	N=52 species of skates and rays that were present in Last et al. 2016's book 'Rays of the World' were excluded from the analyses becuase they were not present in the phylogeny.
Reproducibility	All data and code provided will ensure reproducibility of our findings. Code used in the data analysis is available using the following link: https://osf.io/k6cy9/overview?view_only=8d3769f32fbf4a1ba4ffb0f4c26e2c67
Randomization	No randomization or covariates were used in this study.
Blinding	The comparative analyses presented in this work are not compatible with blinding.

Did the study involve field work? ☐ Yes ☒ No

Reporting for specific materials, systems and methods

We require information from authors about some types of materials, experimental systems and methods used in many studies. Here, indicate whether each material, system or method listed is relevant to your study. If you are not sure if a list item applies to your research, read the appropriate section before selecting a response.

Materials & experimental systems

n/a	Involved in the study
☒	☐ Antibodies
☒	☐ Eukaryotic cell lines
☒	☐ Palaeontology and archaeology
☒	☐ Animals and other organisms
☒	☐ Clinical data
☒	☐ Dual use research of concern
☒	☐ Plants

Methods

n/a	Involved in the study
☒	☐ ChIP-seq
☒	☐ Flow cytometry
☒	☐ MRI-based neuroimaging

Plants

Seed stocks	*Report on the source of all seed stocks or other plant material used. If applicable, state the seed stock centre and catalogue number. If plant specimens were collected from the field, describe the collection location, date and sampling procedures.*
Novel plant genotypes	*Describe the methods by which all novel plant genotypes were produced. This includes those generated by transgenic approaches, gene editing, chemical/radiation-based mutagenesis and hybridization. For transgenic lines, describe the transformation method, the number of independent lines analyzed and the generation upon which experiments were performed. For gene-edited lines, describe the editor used, the endogenous sequence targeted for editing, the targeting guide RNA sequence (if applicable) and how the editor was applied.*
Authentication	*Describe any authentication procedures for each seed stock used or novel genotype generated. Describe any experiments used to assess the effect of a mutation and, where applicable, how potential secondary effects (e.g. second site T-DNA insertions, mosiacism, off-target gene editing) were examined.*
